# The Impact of Nitrogen-Fixing Bacteria-Based Biostimulant Alone or in Combination with Commercial Inoculum on Tomato Native Rhizosphere Microbiota and Production: An Open-Field Trial

**DOI:** 10.3390/biology13060400

**Published:** 2024-05-31

**Authors:** Giorgia Novello, Elisa Bona, Martina Nasuelli, Nadia Massa, Cristina Sudiro, Daniela Cristina Campana, Susanna Gorrasi, Marie Louise Hochart, Adriano Altissimo, Francesco Vuolo, Elisa Gamalero

**Affiliations:** 1 Dipartimento di Scienze e Innovazione Tecnologica (DISIT), Università del Piemonte Orientale, 15121 Alessandria, Italy; giorgia.novello@uniupo.it (G.N.); nadia.massa@uniupo.it (N.M.); daniela.campana@uniupo.it (D.C.C.); elisa.gamalero@uniupo.it (E.G.); 2 Dipartimento per lo Sviluppo Sostenibile e la Transizione Ecologica (DISSTE), Università del Piemonte Orientale, 13100 Vercelli, Italy; martina.nasuelli@uniupo.it; 3 Center on Autoimmune and Allergic Diseases (CAAD), Università del Piemonte Orientale, 28100 Novara, Italy; 4 Landlab S.r.l., 36050 Quinto Vicentino, Italy; c.sudiro@landlab.net (C.S.); marie.louise.hochart@gmail.com (M.L.H.); a.altissimo@landlab.net (A.A.); 5 Dipartimento di Scienze Ecologiche e Biologiche, Università degli Studi della Tuscia, 01100 Viterbo, Italy; gorrasi@unitus.it; 6 Sacco S.r.l., 22071 Cadorago, Italy; f.vuolo@saccosrl.it

**Keywords:** SynCom, rhizosphere, microbiota, soil metabarcoding, tomato, plant-growth promoting bacteria

## Abstract

**Simple Summary:**

The sustainability of the tomato industry is increasingly debatable, necessitating innovative approaches to maximize productivity while minimizing environmental impact. The exploration of Synthetic Communities (SynComs) comprising nitrogen-fixing bacteria and mycorrhizal fungi represents a promising avenue toward achieving this balance. This work demonstrates that SynComs not only support tomato production under reduced fertilizer inputs, but also contribute to enhancing soil microbial biodiversity. This dual benefit underscores the potential of SynComs as a soil management strategy, offering a pathway toward sustainable agriculture and environmental stewardship.

**Abstract:**

The agricultural sector is currently encountering significant challenges due to the effects of climate change, leading to negative consequences for crop productivity and global food security. In this context, traditional agricultural practices have been inadequate in addressing the fast-evolving challenges while maintaining environmental sustainability. A possible alternative to traditional agricultural management is represented by using beneficial micro-organisms that, once applied as bioinoculants, may enhance crop resilience and adaptability, thereby mitigating the adverse effects of environmental stressors and boosting productivity. Tomato is one of the most important crops worldwide, playing a central role in the human diet. The aim of this study was to evaluate the impact of a nitrogen-fixing bacterial-based biostimulant (*Azospirillum* sp., *Azotobacter* sp., and *Rhizobium* sp.) in combination or not with a commercial inoculum Micomix (*Rhizoglomus irregulare*, *Funnelliformis mosseae*, *Funnelliformis caledonium*, *Bacillus licheniformis*, and *Bacillus mucilaginosus*) (MYC) on the native rhizosphere communities and tomato production. Bacterial populations in the different samples were characterized using an environmental metabarcoding approach. The bioinocula effect on the native rhizosphere microbiota resulted in significant variation both in alpha and beta diversity and in a specific signature associated with the presence of biostimulants.

## 1. Introduction

The agricultural sector is currently encountering significant challenges due to the effects of climate change, which could lead to negative consequences for crop productivity and global food security. These impacts can be categorized as direct and indirect. Direct impacts are caused by physical changes such as temperature and precipitation patterns, directly affecting agricultural production systems. On the other hand, indirect effects target other species like pollinators, pests, and invasive species, which further influence crop production (FAO report 2015, Climate change and food security: risks and responses) [1].

A multi-model study conducted by the Intergovernmental Panel on Climate Change (IPCC), using a scenario of unchanging climate, predicted that, by 2050, a mean reduction of 17% in yields for four major crops globally (coarse grains, oilseeds, wheat, and rice), accounting for about 70% of the global crop harvested area, is expected (https://archive.ipcc.ch/ipccreports/tar/wg2/index.php?idp=213, visited 5 April 2024). Obviously, this projection raises concerns, especially considering the predicted global population (9.7 billion by 2050), which will exert an even more significant pressure on agricultural systems. Reports from the World Food Programme (WFP) in 2018 highlighted that crop productivity is increasing slower than demographic rates, leading to almost 800 million people experiencing chronic undernourishment, including 161 million children under the age of five who suffer from stunting (https://www.fao.org/3/i5188e/I5188E.pdf, visited 24 July 2023).

Given the changing climatic conditions, traditional agricultural practices have been inadequate in addressing the fast-evolving challenges while maintaining environmental sustainability. Thus, there is a rising demand for innovative and sustainable solutions. A possible alternative to traditional agricultural managing is represented by beneficial micro-organisms, like Plant-Growth-Promoting Bacteria (PGPB) and mycorrhizal fungi, that, once applied as bioinoculants, may enhance crop resilience and adaptability to changing environmental conditions, thereby mitigating the adverse effects of environmental stressors and boosting productivity [2,3,4,5].

PGPB and mycorrhizal fungi, along with other beneficial micro-organisms, have been extensively studied for their ability to improve nutrient uptake [6,7,8,9,10,11], enhance plant tolerance to environmental stress [2,8,12,13,14,15,16], increase crop yields [17,18,19,20], and improve the nutritional qualities of fruits [21,22] and edible seeds [23]. Numerous studies have indicated that microbial consortia tend to outperform individual strains in various tasks (the breakdown of complex compounds, implementation of multi-step reactions, degradation of plant recalcitrant polymers, etc.) [5,24]. The main advantage of building up and using a synthetic community is the possibility to exploit diverse physiological feautures expressed by the single members of the consortia, and then compensating for the possible deficiencies of individual bacterial strains. Therefore, the use of microbial consortia, combining multiple beneficial micro-organisms, has also gained attention due to their potential to achieve superior plant-growth-promoting outcomes, compared to single-microbe inoculation [5,25]. However, creating an effective consortium presents a significant hurdle. When dealing with a basic consortium consisting of only two bacterial strains, a minimum of six types of relationships, such as commensalism, competition, predation, neutralism, co-operation can be established [26]. Furthermore, as the number of members in a consortium increases, the complexity of potential interactions among its members becomes even greater. In a three or four member consortium about 729 and 531,441 interactions are likely to occur, respectively [26]. Moreover, all these possible interactions are affected by the environmental conditions, and, in turn, the results of these interactions can affect the development of the plants, as well as their associated microbiota [27].

Nonetheless, the scientific community has shown great enthusiasm for the possible use of plant-beneficial micro-organisms, sometimes overlooking concerns about the potential impact of plant inoculation on the native microbial community in the soil and rhizosphere. In fact, there is limited monitoring and information available on the effects of deliberate biofertilizer release on the resident bacterial community [27].

Despite significant progress in understanding the benefits of microbial inoculants in controlled laboratory settings, only limited and contradictory information about their effects on native microbial communities in actual agricultural fields are available. The complexity of the soil microbiota and its interactions with introduced micro-organisms make it challenging to predict the long-term consequences of microbial inoculation on soil biodiversity and ecosystem functioning [26,27]. Rigorous testing and approval procedures for bioinoculants, considering their effects on native soil microbiota and overall ecosystem functioning, are crucial in promoting sustainable and environmentally responsible agricultural practices. Therefore, investigating the interactions between microbial inoculants and native microbial communities in field-grown crops becomes essential in order to comprehensively assess the feasibility and sustainability of these agricultural practices [27].

By understanding how native microbial populations respond to microbial inoculation, valuable insights can be gained regarding the potential risks and benefits associated with these biotechnological approaches in real-world agricultural scenarios. This understanding is a crucial step towards developing efficient and environmentally friendly agricultural strategies that can enhance crop resilience and address the challenges posed by climate change [27,28,29].

In a previous study, the impact of a bacterial-based biostimulant (*Pseudomonas fluorescens* and *Bacillus amyloliquefaciens*—PSBA), alone or in combination with a commercial inoculum (Micomix) containing both Arbuscular Mycorrhizal (AM) fungi (*Rhizoglomus irregulare*, *Funnelliformis mosseae*, *Funnelliformis caledonium*) and PGPB (*Bacillus licheniformis*, *Bacillus mucilaginosus*), on the resident microbiota was assessed before the plant inoculum, at flowering time, and at fruit setting, by using a metabarcoding approach. The bacteria present in the inoculum used in the previous study had, as their main physiological activities, the ability to solubilize phosphate, to produce siderophores and phytohormones. The study reported striking differences in the microbiota composition of each treatment group, with the co-inoculation of PGPBs and AM fungi demonstrating better performance compared to other treatments. Moreover, a subtle yet impactful influence on the soil microbial community suggested a “silent” effect of the bioinocula [28]. However, the current regulations for biostimulants (2019/1009 law by the European Commission) impose restrictions on the choice of plant-beneficial micro-organisms to be used as bioinoculants. Only four microbial types are allowed, including *Azospirillum, Azotobacter, Rhizobium* (bacterial genera involved mainly in nitrogen fixation)*,* and arbuscular mycorrhizal fungi [30]. Therefore, this study specifically focuses on the effects induced by a bioinoculant whose micro-organisms are approved by the European Union (EU) on the tomato resident rhizosphere microbiota. By exploring the potential benefits of this approved bioinoculant, its compatibility with current agricultural practices and its ability to enhance soil health, plant growth, and, ultimately, crop yields can be better understood. The aim of this study is to provide additional insights into the mechanisms underlying the interactions between bioinoculants working mainly in nitrogen fixation and native soil communities, paving the way for more efficient and ecologically friendly agricultural practices. In addition, the second important aim of this work was to assess whether nitrogen-fixing rhizosphere bacteria had a similar or completely different impact compared to rhizosphere PGPB bacteria with a different biochemical profile.

## 2. Materials and Methods

### 2.1. Experimental Design and Field Conditions

The experiment aims to evaluate the effect of the inoculation of nitrogen-fixing bacteria, both alone and in combination with fungal inocula, in real-soil conditions and under a reduced fertilization setup.

The bioinocula were formulated with the collaboration of Sacco s.r.l., which provided the bacterial strains, and Atlantica Agricola (Alicante, Spain), who supplied the Micomix inocula. Micomix is a consortium of three species of mycorrhizal fungi and two strains belonging to the genera *Bacillus* spp., as described above. Specifically, the equivalent concentration of the organisms in the consortium was 12,500 propagules/g for the fungi and 1 × 10^10^ CFU/g for the rhizobacteria. Product formulation was provided by the producer and application doses were applied following the manufacturer’s instructions.

The design of the field plot comprises 5 different treatments: (1) control full NPK (CFD): uninoculated plants fertilized according to conventional practice; (2) control reduced NPK (CRD): uninoculated plants at reduced fertilization conditions; (3) MYC: plants inoculated with the consortium Micomix (Atlantica Agricola, Alicante, Spain), with reduced NPK; (4) NFB: plants inoculated with nitrogen-fixing bacteria belonging to the genera *Azospirillum*, *Azotobacter,* and *Rhizobium*, with reduced NPK; and (5) NFB + MYC: plants inoculated with nitrogen-fixing bacteria *Azospirillum*, *Azotobacter, Rhizobium,* and Micomix consortium, with reduced NPK.

**Figure 1:**
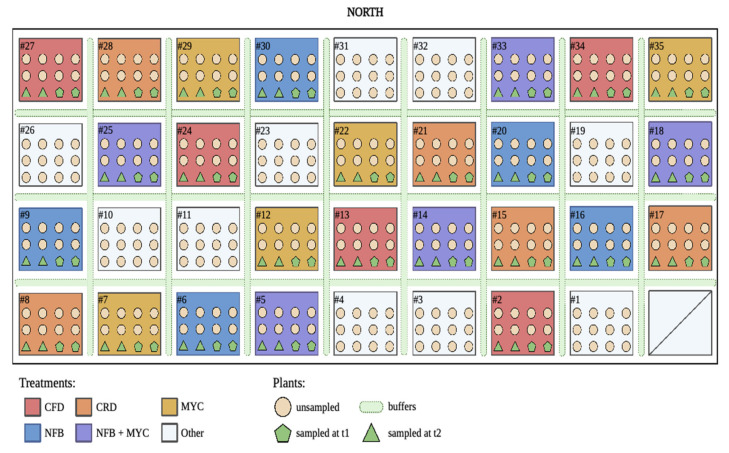
Experimental design. 36 randomized complete blocks were designed to test the 5 different formulated treatments, tested in 5 replicates, each one consisting of 12 plants. A color scheme was assigned to each treatment and described in the legend: red for the uninoculated control plants with a full dose of NPK (CFD); orange for the uninoculated control plants with a reduced dose of NPK (CRD); yellow for plants inoculated with Micomix consortium (MYC), with reduced NPK; blue for plants inoculated with *Azospirillum, Azotobacter,* and *Rhizobium* (NFB), with reduced NPK; and purple for plants inoculated with *Azospirillum, Azotobacter, Rhizobium,* and the Micomix consortium (NFB + MYC), with reduced NPK. Circles indicate unsampled plants, while green symbols represent the tomato plants sampled at t1 (flowering phase) and t2 (fruit setting). Light green dotted lines belong to buffer plants used to separate the treated plots. Only reduced NPK fertigation was applied to these plants (image of the plot created with BioRender.com).

The study was conducted in open fields at Landlab (Landlab srl Società Benefit, Quinto Vicentino (VI), Italy, 45.57° N, 11.62° E, 33 m.a.s.l.). The treatments were applied in 36 randomized complete blocks, with the 5 different treatments replicated 5 times in the plot. For each plot, 12 plants were tested, for a total number of 300 tomato plants (5 treatments × 5 replicates × 12 plants/plot), plus buffer plants between each treated plot (Figure 1). The variety subject of the study is *Solanum lycopersicum* var. Big Rio, usually cultivated for industrial purposes.

**Figure 2:**
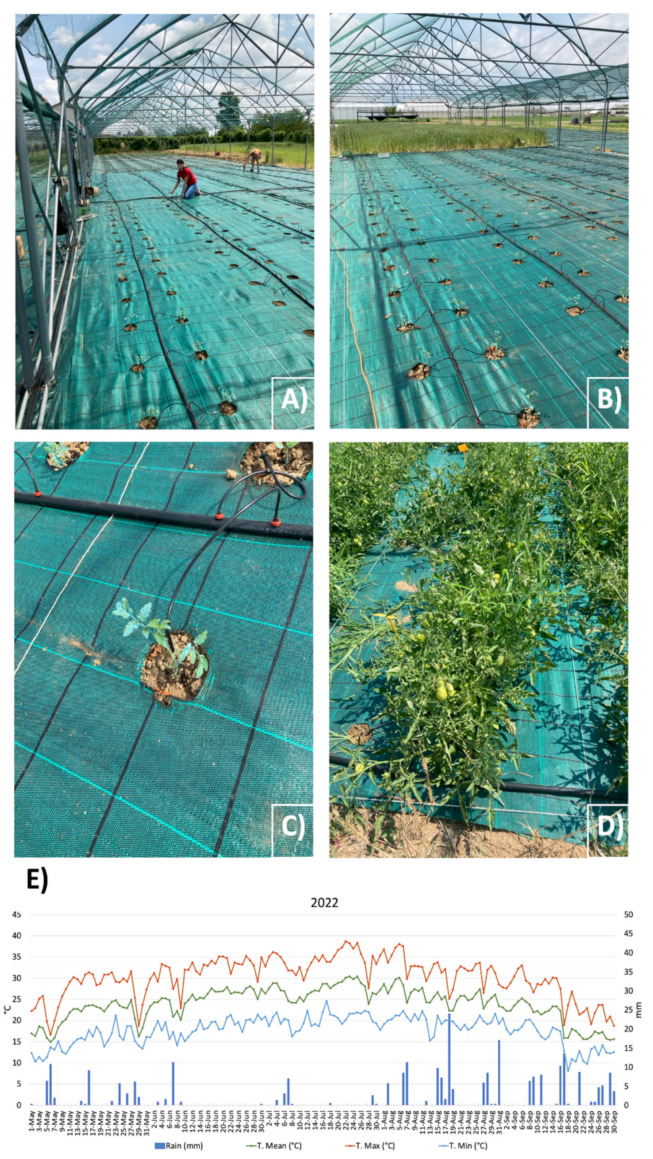
Experimental field and weather conditions: (**A**) experimental setup in Landlab srl; (**B**) drop irrigation system in the field; (**C**) detail of a plant at the time of transplanting in the field and positioning of irrigation; (**D**) detail of plants at second sampling time; and (**E**) weather conditions at Landlab s.r.l. during the experimental period. Green line, mean Temperature (°C), red line, maximum temperature (°C), blue line, minimum temperature (°C).

Field fertilization was dispensed by basal dressing and fertigation. For conventional fertilization (CFD), a basal dressing was made before transplanting, with N (45 kg/ha), P_2_O_5_ (70 kg/ha) and K_2_O (72 kg/ha), while the remaining was given by fertigation: N (105 kg/ha) and K_2_O (168 kg/ha). Reduced NPK supplies consisted of a base dressing with N (31.5 kg/ha), P_2_O_5_ (49 kg/ha), and K_2_O (50.4 kg/ha), while the remaining was given by fertigation: N (105 kg/ha) and K_2_O (168 kg/ha), distributed on all bioinoculant-treated/CRD plots, and buffer plants. Drip irrigation was implemented in the field and supplied at plant’s needs (Figure 2).

Bioinoculants were provided at planting (0 Days After Planting, DAP) and at 20 DAP.

Phyto-chemicals for pest control were applied only at occurrence, summarized as follows: 24.06.22: Pergado, Sivanto (Syngenta); 06.07.22: Ridomil gold R (Syngenta); 15.07.22: Matacar (Sipcam), Vertimec (Syngenta), Movento (Bayer); 22.07.22: Aspor (Isagro spa); 29.07.22: Oikos (Sipcam), Matacar (Sipcam); 05.08.22: Aspor; and 12.08.22: Oikos, Sivanto. For weed control, a permeable mulching was used, which also reduced water loss by evaporation. A hailstorm net was provided to protect the plants from possible adverse weather conditions. Environmental conditions (e.g., air temperature and rains) were collected by Landlab weather station (Figure 2E).

### 2.2. Soil Collection and Analyses

The rhizosphere soil was sampled during different phases of tomato-growing season, in particular: at basal condition before the provision of biostimulants and transplant (t0), at the beginning of flowering (t1), and, lastly, at fruit setting (t2). The timepoints were set to evaluate the dynamics in the native microbiota after the bioinoculant application. A total of 10 samples were collected at t0, while 2 plants per plot were sampled at timepoints t1 and t2, for a total of 50 soil samples/phase (Figure 1). Samples were stored in freezer bags in the field, and, subsequently, they were stored and maintained at −80 °C, until the DNA extraction. Physical–chemical analyses were performed on each soil sample according to D.M. 13/09/99.

### 2.3. Root AM Colonization Assay 

The root system was taken from each plant used for soil sampling to assess the degree of mycorrhizal infection. A sample of 40 randomly chosen 1 cm-long root pieces for each plant was assessed. These root samples were cleared in 10% KOH for 45 min at 60 °C, stained with 1% methyl blue in lactic acid and mounted on a slide. Mycorrhizal colonization was estimated according to Trouvelot and coworkers [31]: frequency of mycorrhization (F%), mycorrhizal degree (M%), frequency of arbuscules (A%), and frequency of vesicles (V%) were calculated. Data were statistically analyzed by one-way ANOVA (using “label” as factor) followed by Tukey HSD post hoc test with Bonferroni-adjusted *p*-values. A two-way ANOVA was also performed using “time” and “treatment” as factors. Differences were considered significant for *p*-values < 0.05. 

### 2.4. Microbial Community Characterization and Bioinformatic and Statistical Analyses

The metagenomic workflow and the bioinformatic and statistical analyses were performed according to Nasuelli et al. [28]. Briefly, total genomic DNA was extracted from 0.25 g of soil using the DNeasy^®^ PowerSoil^®^ Kit (Qiagen, Milan, Italy), quantified through a fluorimetric method and normalized to 10 ng/µL. The procedure followed for the library preparation was according to Illumina 16S Metagenomic Sequencing Library Preparation protocol, amplifying the hypervariable V3–V4 regions. Libraries were sequenced on a NovaSeq instrument (Illumina, San Diego, CA, USA), using 250 bp paired-end mode.

Fastq elaborations were performed with Illumina BCL Convert v3.9.31. Then, trimming of low-quality bases at 3′-tails and primer sequence at 5′-ends were performed, and, in addition, only reads with a minimum length of 200 bp were retained. Following the QIIME pipelines, the USEARCH algorithm (version 8.1.1756, 32-bit) allowed the chimera filtering, grouping of replicate sequences, sorting sequences per decreasing abundance, and OTU identification. Only paired-end reads with an overlap at their 3′-ends were merged. OTUs were built de novo with a clustering threshold set at 97%. OTU sequences not matching any reference sequence of the database constitute a novel OTU and the most abundant and long read in each OTU was selected as the representative sequence. OTUs in “open-reference” analysis were generated with a minimum of 2 sequenced fragments. The RDP classifier and Reference database were used to assign taxonomy while Silva138 database was employed as 16S rDNA sequence reference. Statistical analyses to compare the different microbial communities associated to the different considered conditions were performed using MicrobiomeAnalyst as fully described in Nasuelli et al. [28] using data filtering to identify and remove features having low count and variance, and minimum counts 4, and considering median abundance value. 

### 2.5. Tomato Fruit Sampling and Analyses

Tomato fruits were collected at maturity before harvest time, and divided in two categories (i.e., marketable and unmarketable). Unmarketable tomatoes were further divided into green fruits and tomatoes affected by blossom end-rot (BER). Labeled fruits were then counted and weighed. Analysis of variance (ANOVA) was performed to assess the influence of the treatment on the fruit quality with XLSTAT software 2022.4.1 (Addinsoft, 2021—Paris, France). Comparison of treatment means was carried out using the Duncan test at significant level of *p*-value < 0.05.

## 3. Results

### 3.1. Mycorrhizal Degree

**Figure 3:**
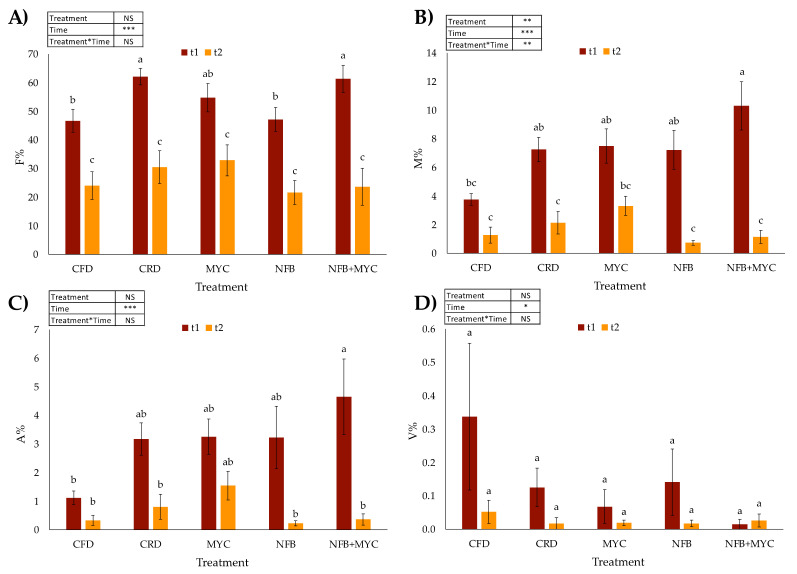
Mycorrhizal colonization in tomato plants. F%: frequency of mycorrhization (**A**), M%: mycorrhizal degree (**B**), A%: frequency of arbuscules (**C**) and V%: frequency of vesicles (**D**). Mean values and relative standard errors are reported. Different letters in the same color bars indicate significantly different values (*p* < 0.05) based on one-way ANOVA followed by Tukey HSD post hoc test with Bonferroni-adjusted *p*-values. *n* = 10. Statistically significant differences based on two-way ANOVA are also reported in the text as follows: (**A**) F% treatment NS (Not Significant), time ***, treatment × time NS; (**B**) M% treatment **, time ***, treatment × time **; (**C**) A% treatment NS, time ***, treatment × time NS; (**D**) V% treatment NS, time *, treatment × time NS (where * *p* < 0.05; ** *p* < 0.01; *** *p* < 0.001).

The mycorrhizal colonization degree is presented in Figure 3.

The mycorrhizal colonization degree was statistically significantly modulated by both treatment and time factors (Figure 3), considering the two-way ANOVA results. Although uninoculated, the plants in CFD and CRD treatments showed a mycorrhization degree (M%) of 3.7 ± 1.4 (t1) and 1.3 ± 0.6 (t2) in CFD and of 7.2 ± 0.9 (t1) and 2.1 ± 0.8 (t2) in CRD, respectively. The other mycorrhizal treatments (MYC and NFB + MYC) showed an M% of 7.5 ± 1.2 (t1) and 3.3 ± 0.7 (t2) in MYC and 10.3 ± 1.7 (t1) and 1.1 ± 0.5 (t2) in NFB + MYC. Considering the other parameters (F%, A%, and V%), they were negatively affected only by the time factor (different phenological strategies of the plants). In fact, both F% and A% were statistically different in CFD and NFB vs. CRD and NFB + MYC at t1. However, MYC was not statistically different from any of the other treatments. Inoculation with mycorrhizae has no effect on mycorrhizal frequency (CRD vs. MYC and NFB + MYC). At time of fruit setting, there was no statistical significance between any of the conditions for any of the parameters. 

Soil physical–chemical characteristics were fully reported in Nasuelli et al. [28]. The soil was classified as alkaline, considering the pH of 8.29. The electrical conductivity (EC) was low. The active lime was in the reference ranges (1.93%), while the available phosphorus was low (19.3). Organic matter and total nitrogen are in the reference ranges (1.69 and 1.038, respectively), while the C/N ratio was 9.44, slightly lower than standard values. Compared to the micronutrient elements, the boron quantity yielded a low result (<0.50), while the values of iron, manganese, and copper were higher than the reference ranges. Only zinc presented values in the standard intervals.

For the exchange complexes, exchangeable sodium and potassium showed very low and low quantities, respectively, while ion calcium was listed as very high. Ion magnesium had a normal value of 2.39. Base saturation and CEC were, respectively, normal and very high compared to the reference ranges.

### 3.2. Tomato Production

**Figure 4:**
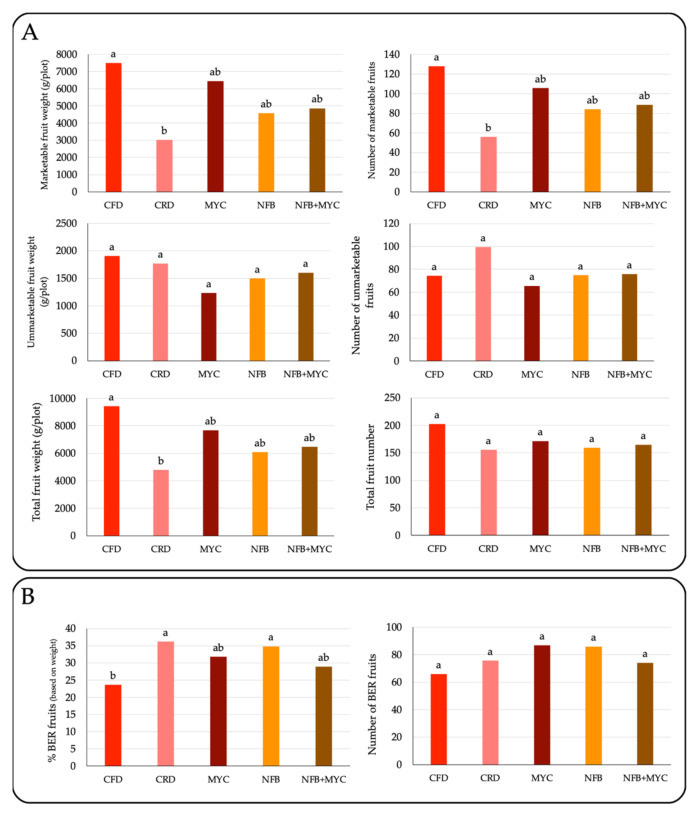
Marketable and unmarketable (green fruits) fruit number and weight/plot (**A**), and % of BER fruits (**B**). Different letters indicate statistically significant differences between the treatments according to the Duncan test (*p* < 0.05).

Figure 4 reports the results concerning the tomato production at the end of the experiment (harvest time). 

The fertilization cut induced a statistically significant reduction in the number and weight of marketable fruit. The use of biostimulants, either alone or in combination, always under reduced fertilization conditions, restored production to levels comparable to the control with full fertilization (CFD).

### 3.3. Soil Microbiota Profiling 

A total of 133,072,948 reads were obtained with a mean value of 1,073,169 reads per sample. The genomic sequences were included in the BioProject PRJNA916628, titled “Impact of PGPB bacteria and AM fungi inocula on resident communities associated with tomato roots”, available in the NCBI database (https://submit.ncbi.nlm.nih.gov/subs/sra/SUB12451409/overview, accessed on 20 December 2022). The BioProject contains 122 BioSamples. 

**Figure 5:**
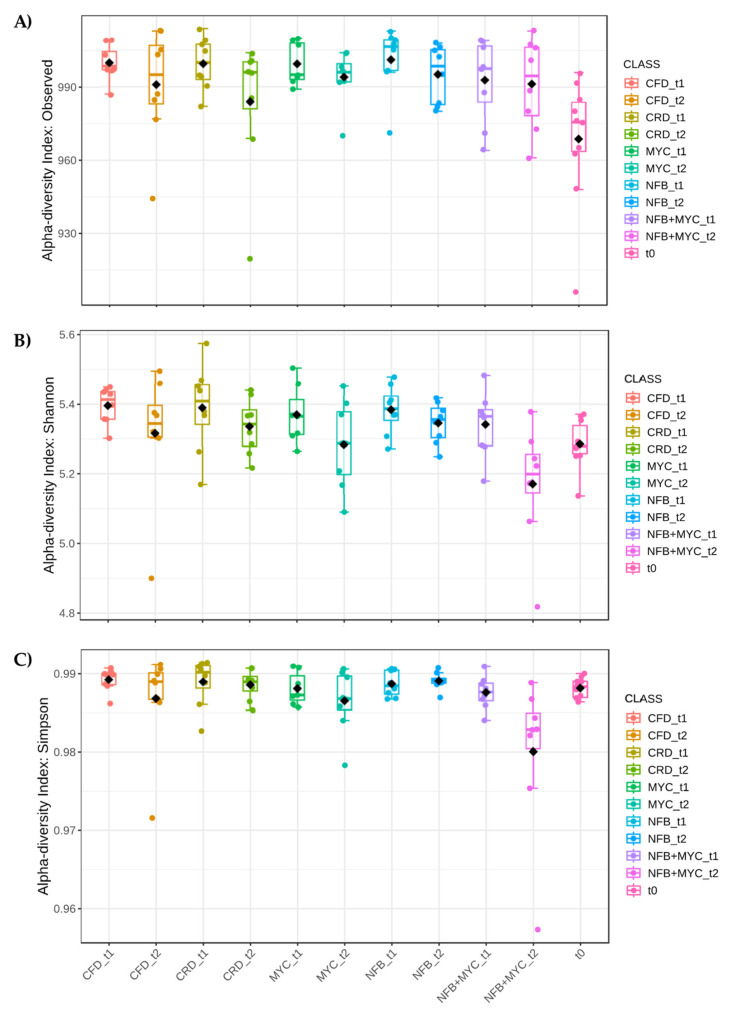
Alpha diversity analysis considering the Class. (**A**) Number of observed species (*p*-value 0.0357), (**B**) Shannon index (*p*-value 0.0134), and (**C**) Simpson’s index (*p*-value 0.0369). In the figure, black diamond indicated the mean value, while the line inside the box represented the median value. Alpha diversity analysis was performed using the phyloseq package of Microbiome Analyst. CFD—uninoculated control plants with a full dose of NPK; CRD—uninoculated control plants with a reduced dose of NPK; MYC—plants inoculated with the commercial inoculum Micomix, with reduced NPK; NFB—plants inoculated with *Azospirillum, Azotobacter,* and *Rhizobium*, with reduced NPK; NFB + MYC—plants inoculated with *Azospirillum, Azotobacter, Rhizobium,* and the commercial inoculum Micomix, with reduced NPK. t0 (condition before the experiment setup), t1 (flowering phase), and t2 (fruit setting).

**Figure 6:**
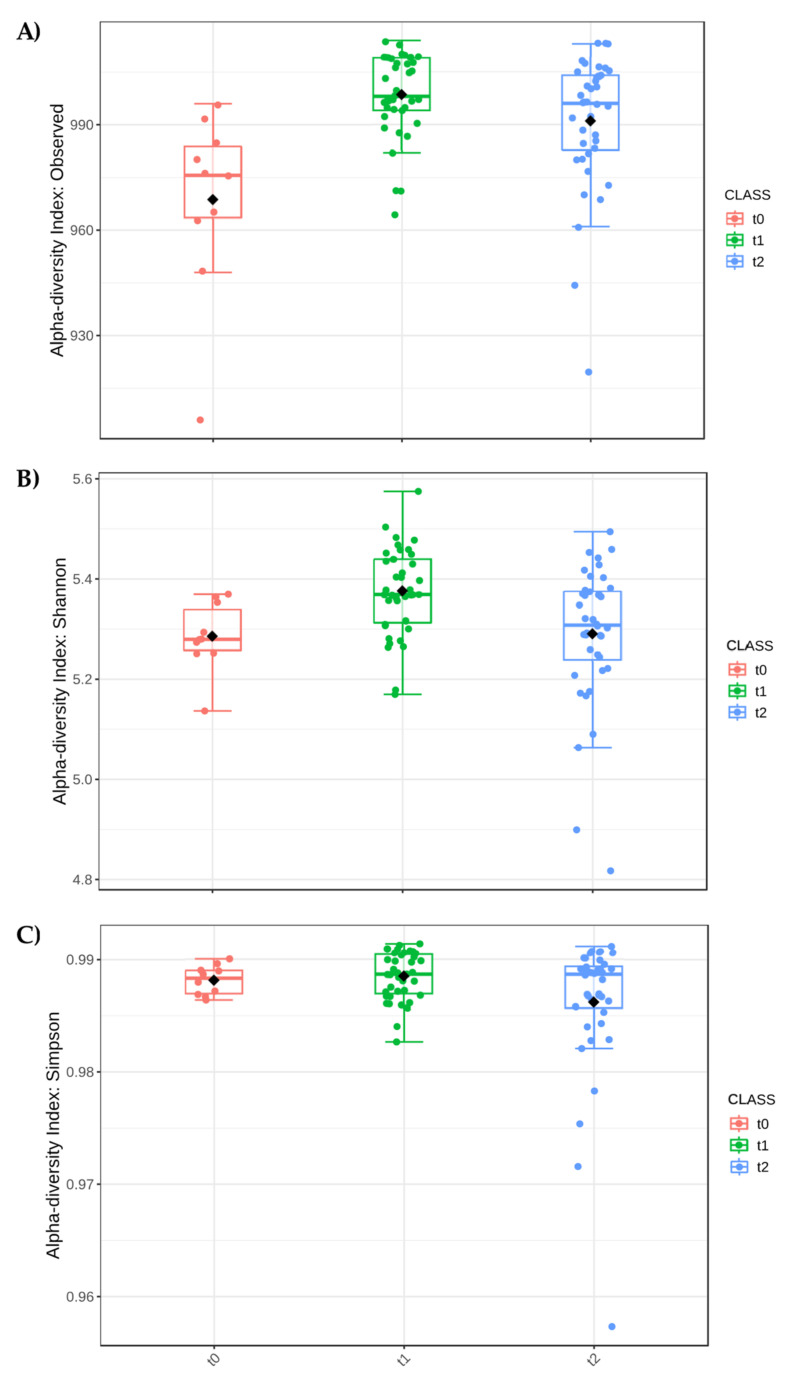
Alpha diversity analysis considering Time (Class). (**A**) Number of observed species (*p*-value 0.00017), (**B**) Shannon index (*p*-value 0.0018), and (**C**) Simpson’s index (*p*-value 0.2888). *p*-value cut-off for significance is 0.05. In the figure, black diamond indicated the mean value while the line inside the box represented the median value. Alpha diversity analysis was performed using the phyloseq package of Microbiome Analyst. t0 (condition before the experiment setup), t1 (flowering phase), and t2 (fruit setting).

**Figure 7:**
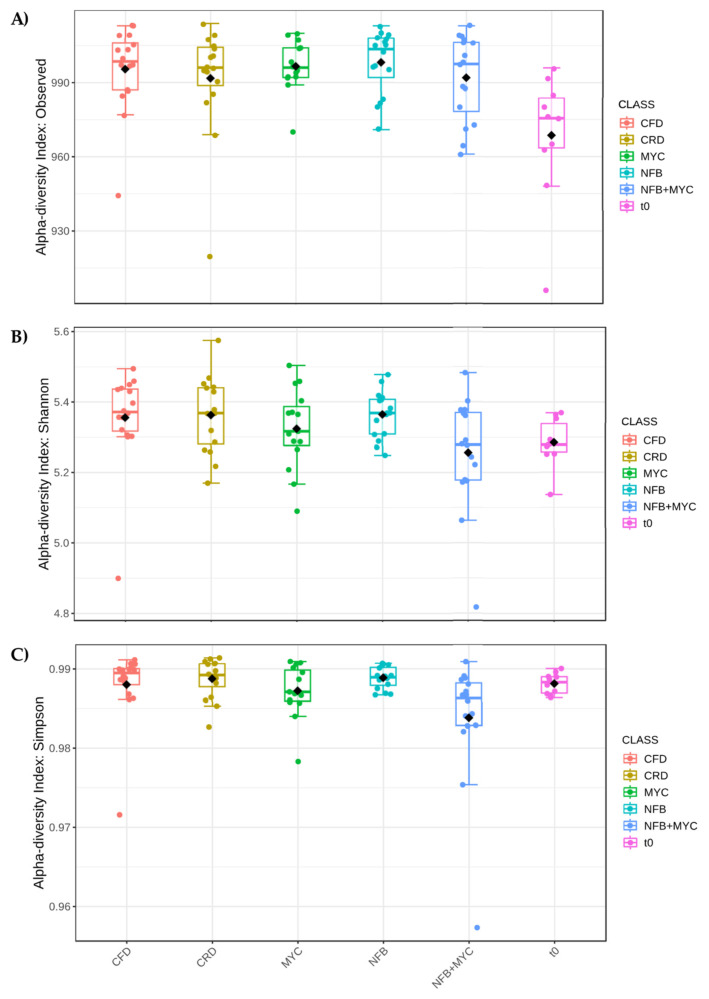
Alpha diversity analysis considering Treatment (Class). (**A**) Number of observed species (*p*-value 0.0112), (**B**) Shannon index (*p*-value 0.024), and (**C**) Simpson’s index (*p*-value 0.0142). *p*-value cut-off for significance is 0.05. In the figure, black diamond indicated the mean value while the line inside the box represented the median value. Alpha diversity analysis was performed using the phyloseq package of Microbiome Analyst. CFD—uninoculated control plants with a full dose of NPK; CRD—uninoculated control plants with a reduced dose of NPK; MYC—plants inoculated with the commercial inoculum Micomix, with reduced NPK; NFB—plants inoculated with *Azospirillum, Azotobacter,* and *Rhizobium*, with reduced NPK; NFB + MYC—plants inoculated with *Azospirillum, Azotobacter, Rhizobium,* and the commercial inoculum Micomix, with reduced NPK. t0 (condition before the experiment setup).

The basal soil, sampled before tomato planting and before the insertion of the biostimulant inocula (synthetic community, SynCom) (t0), presented a rather marked biodiversity (alpha diversity), characterized by a number of 969.7 ± 8.4 (mean ± standard error) observed genera (Shannon index 5.3 and Simpson index 0.988) (Figure 5, Figure 6 and Figure 7), organized mainly into fourteen phyla and 2% in which we included phyla represented less than 0.5%. In particular, the most abundant phylum was *Actinobacteriota* (31.5%) followed by *Pseudomonadota* (14.8%), *Acidobacteriota* (12.9%), *Planctomycetota* (9.8%), *Chloroflexi* (9.4%), *Firmicutes* (5.9%), *Verrucomicrobiota* (4%), *Mixococcota* (2.5%), *Gemmatimonadota* (2.2%), *Bacteroidota* (1.6%), “Candidatus *Methylomirabilota”* (1.1%), “*Entotheonellaeota”* (0.7%), *Nitrospirota* (0.6%), and *Armatimonadota* (0.5%). Considering the genus identification, 46.9% were unassigned and 22.4% were genera present at less than 0.5%. The identified genera were: *Arthrobacter* (5.25%), *Gaiella* (3.41%), *Bacillus* (3.28%), *Sphingomonas* (1.56%), uncultured *Acidobacteriaceae* bacterium (1.41%), *Rubrobacter* (1.3%), “*Candidatus Xiphinematobacter”* (1.21%), *Microvirga* (1.13%), uncultured *Chloroflexi bacterium* (1.11%), *Gemmata* (1.1%), uncultured *Acidobacteria* bacterium (1.09%), *Knoellia* (1.08%), *Nocardioides* (1.03%), *Pirellula* (1%), *Paenibacillus* (0.91%), uncultured *Acidobacteriales* bacterium (0.84%), *Marmoricola* (0.71%), uncultured *Rubrobacterales* bacterium (0.69%), *Nitrospira* (0.62%), uncultured *Actinobacterium* (0.58%), *Skermanella* (0.57%), and *Massilia* (0.5%).

The microbiota associated with the different treatments presented a statistically significant modulation of the biodiversity indices as shown in Figure 5. In particular, the number of observed genera were in: CFD_t1 1000.9 ± 2.5, CFD_t2 991.8 ± 8.0, CRD_t1 1001.0 ± 4.1, CRD_t2 983.6 ± 10.3, MYC_t1 1000.0 ± 3.4, MYC_t2 995.3 ± 4.0, NFB_t1 1001.6 ± 5.0, NFB_t2 995.1 ± 4.3, NFB + MYC_t1 993.8 ± 6.0, and NFB + MYC_t2 991.8 ± 6.8. Shannon and Simpson indices showed two partially different trends, even if in a high biodiversity range (Simpson index around 0.99, considering the maximum at 1). 

As shown in Figure 6, the factor Time was significant in the modulation of the biodiversity indices; particularly, the t1 (flowering time) had the most influence on both the number of the genera and the Shannon index, that considers the number of species living in a habitat (richness) and their relative abundance (evenness). The Simpson index did not show differences in the different times, but was always high with a value around 0.99. Finally, considering only the factor treatment (Figure 7) and not considering time, all the treatments in which the plants were present resulted in higher biodiversity indices.

**Figure 8:**
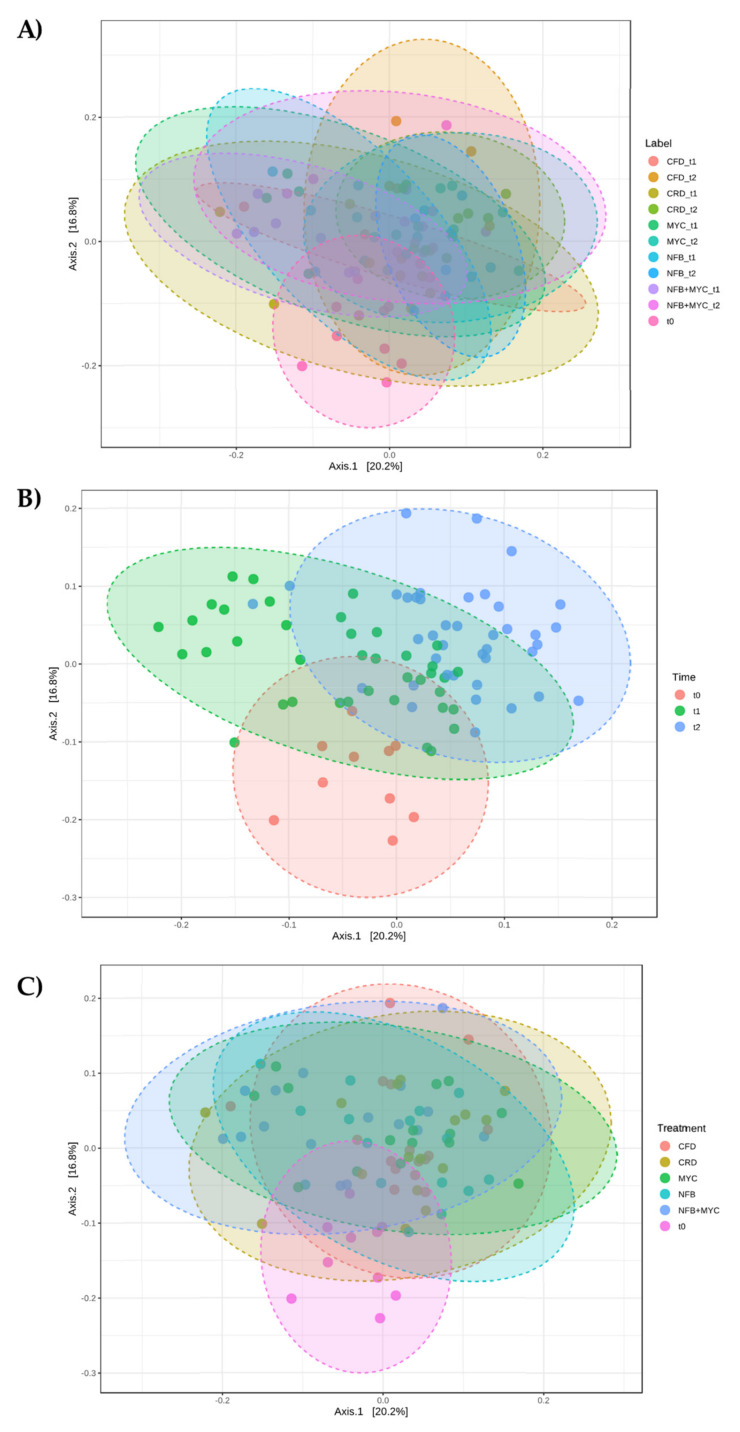
Beta diversity analysis at the genus level. Principal co-ordinate analysis (PCoA) based on Bray–Curtis metrics shows the dissimilarity of bacterial communities in the different soils according to (**A**) Label (*p*-value 0.001), (**B**) Time (*p*-value 0.001), and (**C**) Treatment (*p*-value 0.001). Beta diversity analysis was performed using Microbiome Analyst. CFD—uninoculated control plants with a full dose of NPK; CRD—uninoculated control plants with a reduced dose of NPK; MYC—plants inoculated with the commercial inoculum Micomix, with reduced NPK; NFB—plants inoculated with *Azospirillum, Azotobacter,* and *Rhizobium*, with reduced NPK; NFB + MYC—plants inoculated with *Azospirillum, Azotobacter, Rhizobium,* and the commercial inoculum Micomix, with reduced NPK. t0 (condition before the experiment setup).

The microbial community characterizing the basal soil differed in a statistically significant manner from the community associated with the different treatments (Label (corresponding to the different treatments at each time), Time, and Treatments) (beta diversity, *p*-value 0.001; Figure 8). 

#### 3.3.1. Plant Effect

The significant genera influencing the biodiversity associated with the presence of tomato plants are shown in Table S1. Comparing the treatments at each time to the baseline microbiota (t0), about 58.4% (292 genera) of the genera are significantly influenced by the plant. Positive differences greater than 10% are highlighted in green (46.6% of the significant genera), while negative differences greater than 10% are highlighted in red (16.1% of the significant genera). In particular, among the genera positively affected are the following: *Acidibacter*, *Acidovorax*, *Aeromicrobium*, *Allorhizobium*, *Neorhizobium*, *Pararhizobium*, *Rhizobium*, *Altererythrobacter*, *Amylobacter*, *Aquaspirillum*, *Aquicella*, *Arenimonas*, *Aridibacter*, *Armatimonas*, *Asticaccaulis*, *Azohydromonas*, *Azospira*, *Baullia*, *Bdellovibrio*, *Blastocatella*, *Bosea*, *Bradyrhizobium*, *Caenimonas*, *Chelativorans*, *Chlamydomonas*, *Cloronema*, *Chthoniobacter*, *Chtonomonas*, *Cnuella*, *Comamonas*, *Croceicoccus*, *Cupriavidus*, *Deinococcus*, *Devosia*, *Dogdonella*, *Dyadobacter*, *Ellin*, *Ensifer*, *Erytrobacter*, *Ettlia*, *Ferrovibrio*, *Ferruginibacter*, *Fimbriiglobus*, *Flavihumibacter*, *Flavisolibacter*, *Flavitalea*, *Flavobacterium*, *Gemmatimonas*, *Halobacillus*, *Herpetosiphon*, *Hirschia*, *Ideonella*, *Inquilinus*, *Lacibacter*, *Legionella*, *Leptolyngbya* EcFYyyy00, *Longimicrobium*, *Luteimonas*, *Luteolibacter*, *Lysobacter*, *Meiothermus*, *Mesorhizobium*, *Methylobacillus*, *Methylocella*, *Metylopila*, *Metylotenera*, *Microbacterium*, *Micropepsis*, *Mitsuaria*, *Nannocystis*, *Niastella*, *Nitratireductor*, *Nitrosomonas*, *Nitrospira*, *Nodosilinea* PCC7104, *Noviherbaspirillum*, *Ohtaekwangia*, *Oscillatoria* SAG1459 8, *Oscillochloris*, *Panacagrimonas*, *Parasegetibacter*, *Paucibacter*, *Pelomonas*, *Peredibacter*, *Piscinibacter*, *Planctomycete* WY108, *Planctopirus*, *Planoglabratella*, *Poliangiace*, *Polangium, Pontibacter*, *Prostecomicrobium*, *Pseudoduganella*, *Pseudomuriella*, *Pseudorodoplanes*, *Pseudoxanthomonas*, *Oipengyuania*, *Ramlibacter*, *Reyranella*, *Rhizobacter*, *Rhodanobacter*, *Rhodobacter*, *Rhodocytophaga*, *Rhodopirellula*, *Rhodopseudomonas*, *Roseomonas*, *Rubellimicrobium*, *Schlesneria*, *Shinella*, *Simplicispira*, *Sphingobium*, *Sphingopyxis*, *Streptomyces*, *Synechococcus* IR11, *Tahibacter*, *Tepidisphaera*, *Terrimicrobium*, *Terrimonas*, *Truepera*, *Vampirovibrio*, *Variovorax*, *Verrucomicrobia*, *Vischeria* CAUP Q 202, and *Yonghaparkia*. On the contrary, some of the genera negatively affected were as follows: *Acidimicrobia* bacterium, *Acidothermus*, *Actinocorallia*, *Actinopolymorpha*, *Anaerobacterium*, *Anaerolinea*, *Anaeromixobacter*, *Aquisphaera*, *Brevibacillus*, *Brevifollis*, *Burkholderia*, *Caballeronia*, *Parburkolderia*, *Caldicoprobacter*, *Clostridium*, *Desulfosporosinus*, *Dongia*, *Fictibacillus*, *Gaiella*, *Geoalkalibacter*, *Herbinix*, *Kribbella*, *Luedemanella*, *Marmoricola*, *Methyloceanibacter*, *Mucillaginibacter*, *Mycobacterium*, *Nocardioides*, *Pedococcus*, *Phycicoccus*, *Pelosinus*, *Planifilum*, *Rhodococcus*, *Ruminiclostridium*, *Salinispora*, *Sideroxydans*, *Solibacillus*, *Solirubrobacter*, *Spirochaeta*, *Symbiobacterium*, *Syntrophobacter,* and *Thermoactinomyces*.

#### 3.3.2. Effect of Phenological Stage of the Plant 

As previously observed when considering alpha diversity, the phenological phase inducing the greatest stimulation of biodiversity in the rhizosphere was flowering. In particular, this stimulation resulted in the increase in 200 genera, while, at maturity, 185 genera were observed that increased significantly compared to t0 (Tables S2 and S5). In addition, at t1 and t2, 97 and 197 genera, respectively, decreased their frequency. Among the genera with a very marked increase were the following: *Azospira, Deinococcus, Erythrobacter, Halobacillus, Hassalia, Hirschia, Ideonella, Meiothermus, Methylobacillus, Micropepsis, Mitsuaria, Nitrosomonas, Pseudoduganella, Rhodanobacter, Rhodopseudomonas, Shinella, Simplicispira, Sphingobium, Sinechococcus,* and *Vampirovibrio*.

#### 3.3.3. Bioinocula Effect

The used bacterial inoculum induced a significant increase in 63 genera and a decrease in 85 genera compared to the non-inoculated control condition (Tables S3, S4, S6 and S7). In particular, the most significant were *Geoalkalibacter*, *Halobacillus,* and *Meiotermus*. Mycorrhizal inoculation alone induced the significant increase in 64 genera and the decrease in 80 genera. The most significant were *Aminobacter*, *Chelativorans*, *Halobacillus,* and *Mitsuaria*. Finally, considering the inoculum composed of bacteria and fungi, it induced the significant increase in 94 genera and the decrease in 88 genera. Of these, the most significant were *Aminobacter*, *Anaerolinea*, *Chelativorans*, *Ettlia*, *Geoalkalibacter*, *Methylotenera*, and *Terribacillus*. 

## 4. Discussion

Vegetables account for 12% of world agricultural production after cereals and sugar crops. Among vegetables, tomatoes represent the largest production (FAO data 2021) [32], and, over the period 2000–2021, they remained stable at 16%. Tomato is, therefore, among the most important and widespread crops in the world. According to data collected and updated in December 2022 by the FAO, total world tomato production in 2021, both for processing and fresh consumption, was just over 189.1 million metric tons. According to these data, annual tomato production has reached or exceeded the million tons threshold in many countries. The Turkish production (13 million tonnes) was twice that of Italy (6.6 million tons), but accounted for only one-fifth of the Chinese harvest (67 million tons), which alone accounted for almost 36% of the world harvest [32]. Tomato cultivation requires large areas of fertile soil. To increase total world production, the use of bacterial and AM fungal biostimulants could be a solution to make this cultivation more sustainable, reduce the chemical inputs, and preserve/enhance soil microbial biodiversity. Indeed, soil microbial biodiversity sustains and maintains healthy soils, providing the ecosystem services that healthy soils can offer [33].

In this context, on 5 July 2023 the European Commission published the text of the proposed Soil Monitoring and Resilience Directive (Soil Monitoring Law) [34], with the aim of achieving healthy soils throughout the European Union by 2050. The proposal is a key building block in the realization of the European Green Deal (EGD) that aims to transform the economy and society to make the EU the first climate-neutral continent by 2050. The proposed Directive starts from the fact that more than 60% of European soils are currently unhealthy and aims to support actions to improve and maintain soils in a healthy condition, so that they can provide ecosystem services on a scale necessary for environmental, social, and economic needs. The proposed measures consist of actions for: (i) the monitoring and evaluation of soil health; (ii) sustainable soil management; and (iii) the definition, identification, and risk assessment of contaminated sites [34]. 

Based on these ideas, this experimental work had a twofold objective: on the one hand, to evaluate the potential of a Synthetic Community (SynCom) characterized and composed of free-living nitrogen-fixing bacteria (*Azospirillum*, *Azotobacter,* and *Rhizobium*) and Micomix (a consortium of three species of mycorrhizal fungi and two strains belonging to the genus *Bacillus* and *Paenibacillus*) in supporting tomato production by reducing the input of chemical fertilizer; on the other hand, this work aimed to study the impact of the application of this SynCom on soil microbial biodiversity.

The experiment was conducted in an open field on a rather fertile soil with a good degree of microbial biodiversity. For this reason, a basal degree of mycorrhizal colonization, even in controls not inoculated with SynCom, was observed. However, the colonization by the Micomix inoculum was significantly greater in the presence of the nitrogen-fixing bacteria at flowering, whereas, at the time of fruiting, a reduction in the colonization was recorded, as is already widely reported in the literature. The reduction in the degree of mycorrhizal colonization during fruiting is a finding already reported in the literature and could be ascribed to the formation and ripening process of the fruit, which attracts photosynthates that are, therefore, no longer available for the maintenance of mycorrhizal colonization [22,35]. In fact, the authors have observed this trend in mycorrhizal degree with respect to the phenological state of the plant, in other work on open-field tomato cultivation using SynCom formulated with other micro-organisms [21,22]. Furthermore, the bacteria present in SynCom showed a helper effect of mycorrhizal colonization (M %) when combined with the fungal inoculum. The effect of inhibiting colonization at t2 where the bacteria are present was also much more pronounced. This finding, probably due to the nitrogen supply by the bacterial inoculum, is different from the inoculation with non-nitrogen-fixing bacteria observed under the same conditions and in the same field as reported in Nasuelli et al. [28].

The results reporting tomato production, presented in this work, demonstrated that the use of SynComs could support marketable berry production under reduced fertilization conditions. Therefore, this work demonstrates how the use of well-characterized SynComs can be a soil management strategy resulting in the reduction in chemical fertilizer inputs with a possible positive environmental impact by limiting residual pollution deriving from the employment of chemical fertilizers.

Regarding the evaluation of soil microbial biodiversity, as previously introduced, the substrate presented very good values of microbial biodiversity. In this context, however, the addition of the SynCom produced a significant impact on the native microbiota, stimulating an increase in the genera present and improving the biodiversity indices (richness and evenness). The stimulation of biodiversity is also modulated by both plant presence/absence and phenological status. This result had already been reported in Nasuelli et al. [28]’s work in which SynCom with a different bacterial formulation employing non-nitrogen-fixing PGPB bacteria was used.

In particular, considering the modulation induced in the rhizosphere microbiota by the phenological stage of the tomato plants, several genera are of interest: for example, in order to give some details, *Azospira* is a free-living nitrogen-fixing genus of Gram-negative bacteria belonging to the phylum *Pseudomonadota*, described by Reinhold-Hurek and Hurek (2000) [34,35]. *Azospira* is a rhizospheric bacterium specifically present in the rhizosphere of wheat during the wheat–rice rotation [36], also used in the formulation of commercial biostimulants. *Deinococcus* belongs to the *Deinococcaceae* family, and it is a genus of the three in the order *Deinococcales* of the bacterial phylum *Deinococcota,* that is highly resistant to environmental hazards. Currently, 89 species are reported in this genus, and they have a peculiar cell structure that makes them particularly resistant to environmental stresses. *Erythrobacter* is a Gram-negative and rod-shaped bacteria genus from the family *Erythrobacteraceae*, that is one of the four families belonging to the order *Sphingomonadales*, and it is affiliated with the class *Alphaproteobacteria*. This family includes twenty genera for a total of 194 species. Members of the family are Gram-negative, aerobic, rod-shaped, or pleomorphic coccoid bacteria. *Erythrobacter*, similarly to *Azospira*, is a rhizospheric bacterium reported to be present in the rhizosphere of wheat during the wheat–rice rotation [36] and in tomato rhizosphere [37]. *Sphingobium* is a genus commonly isolated from soil; it is reported to degrade a variety of chemicals in the environment, such as aromatic and chloroaromatic compounds [38,39] and it is reported as associated with the tomato rhizosphere [37]. The modulation of bacterial biodiversity observed in the treatments especially during flowering can be associated not only with the stimulating effect of the inocula but also with an increased presence of root exudates that are produced during this phenological state. These carbon compounds favor bacterial proliferation as they constitute its nutrient. The proliferation of the identified beneficial bacteria, on the other hand, supports the plant by supplying increased amounts of nitrogen and other mineral components, as well as phytohormones, supporting its growth even under reduced fertilization conditions [21,22,29,30,40].

The impact of the introduction of SynCom in the biodiversity of the tomato rhizosphere, under the present experimental conditions, is, as previously introduced, important. However, even though, in this study, the authors obtained a well-characterized resolution of the bacterial genera, it is very difficult to describe in detail against which species there is a modulation induced using SynCom. Species determination is important in understanding the interactions that can occur at the rhizosphere level. In fact, it is known in the literature that the presence of certain species can favor the proliferation of species with possible pathogenic activity towards crops [37]. The classification of OTUs was based on the hypervariable regions (HVR) V3–V4 of the 16S gene, widely used in commercial kits for this type of assessment and applied in many soil biodiversity assessment projects [41], but inefficient in describing the modulation of species in detail [42]. This approach can, however, be considered adequate if the purpose of the soil metagenomic analysis is to monitor biodiversity in association with, for example, the use of SynCom. However, in impact studies on the native microbiota, it does not allow a detailed description of what is happening in the rhizosphere. 

Other studies focused on the description of the rhizosphere microbiota [43], and employed the HVR V3–V6 to obtain a higher resolution depth, obtaining the species characterization. It is well-known that longer amplicons result in an increase in the OTU number and identification, consequently allowing more precise descriptions of microbial composition and diversity [44]. Moreover, the combination of more conserved HVRs (i.e., V4, V5, and V6) with mutation-prone HVRs (i.e., V3) led to a more accurate bacteria identification [41], considering also that HVR V3 and V6 were found as the optimal region to discern highly similar bacterial strains [45]. Future characterization of the rhizosphere microbiota with 16S gene metabarcoding should take into account this aspect, in order to evaluate punctual species modifications in the bacterial roots’ community.

## 5. Conclusions

In conclusion, the significance of tomato cultivation as a staple crop globally cannot be overlooked, particularly given its prominence in world agricultural production. However, the sustainability of this vital industry is increasingly under debate, necessitating innovative approaches to maximize productivity while minimizing environmental impact. The exploration of Synthetic Communities (SynComs) comprising nitrogen-fixing bacteria and mycorrhizal fungi represents a promising avenue toward achieving this balance. Through the experimental evaluation detailed herein, it has been demonstrated that SynComs not only support tomato production under reduced fertilizer inputs, but also contribute to the enhancement of soil microbial biodiversity. This dual benefit underscores the potential of SynComs as a soil management strategy, offering a pathway toward sustainable agriculture and environmental stewardship. Moreover, initiatives such as the proposed Soil Monitoring and Resilience Directive by the European Commission reflect a broader commitment to fostering healthy soils, aligning with global efforts towards achieving a more sustainable future. 

## Data Availability

The genomic sequences were included in the BioProject PRJNA916628, titled “Impact of PGPB bacteria and AM fungi inocula on resident communities associated with tomato roots”, available in NCBI database, https://submit.ncbi.nlm.nih.gov/subs/sra/SUB1245140 9/overview, accessed on 20 December 2022.

## Supplementary Materials

The following supporting information can be downloaded at: https://www.mdpi.com/article/10.3390/biology13060400/s1, Table S1. Signature associated to each treatment (Label); Table S2. Signature associated to the different time; Table S3. Data grouped by label. Percentual standardization of values vs. t0; Table S4. Signature associated to each treatment (Label). Data grouped by label. Percentual standardization of values vs. CRD; Table S5. Signature associated to each treatment. Data grouped by treatment. Percentual standardization of values vs. t0; Table S6. Signature associated to each treatment. Data grouped by treatment. Percentual standardization of values 46 vs. CFD; Table S7. Signature associated to each treatment. Data grouped by treatment. Percentual standardization of values 52 vs. CRD.
