# Supplementary material for: The Impact of Nitrogen-Fixing Bacteria-Based Biostimulant Alone or in Combination with Commercial Inoculum on Tomato Native Rhizosphere Microbiota and Production: An Open-Field Trial
Source: Biology (Basel). 2024 May 31;13(6):400. doi: 10.3390/biology13060400 (PMC11200462; doi:10.3390/biology13060400)
Supplement: Supplementary file 1 [file biology-13-00400-s001.zip › biology-3025379-Supplementary.pdf]

Supplementary material

The Impact of Nitrogen-Fixing Bacteria-Based Biostimulant Alone or in Combination with Commercial Inoculum on Tomato Native Rhizosphere Microbiota and Production: An Open-Field Trial

Giorgia Novello [1,†], Elisa Bona [2,3,*,†], Martina Nasuelli [2], Nadia Massa [1], Cristina Sudiro [4], Daniela Cristina Campana [1], Susanna Gorrasi [5], Marie Louise Hochart [4], Adriano Altissimo [4], Francesco Vuolo [6] and Elisa Gamalero [1]

[1] Dipartimento di Scienze e Innovazione Tecnologica (DISIT), Università del Piemonte Orientale, 15121 Alessandria, Italy; giorgia.novello@uniupo.it (G.N.); nadia.massa@uniupo.it (N.M.); daniela.campana@uniupo.it (D.C.C.); elisa.gamalero@uniupo.it (E.G.)
[2] Dipartimento per lo Sviluppo Sostenibile e la Transizione Ecologica (DISSTE), Università del Piemonte Orientale, 13100 Vercelli, Italy; martina.nasuelli@uniupo.it
[3] Center on Autoimmune and Allergic Diseases (CAAD), Università del Piemonte Orientale, 28100 Novara, Italy
[4] Landlab S.r.l., 36050 Quinto Vicentino, Italy; c.sudiro@landlab.net (C.S.); marie.louise.hochart@gmail.com (M.L.H.); a.altissimo@landlab.net (A.A.)
[5] Dipartimento di Scienze Ecologiche e Biologiche, Università degli Studi della Tuscia, 01100 Viterbo, Italy; gorrasi@unitus.it
[6] Sacco S.r.l., 22071 Cadorago, Italy; f.vuolo@saccosrl.it
* Correspondence: elisa.bona@uniupo.it
† These authors contributed equally to the work.

Table S1. Signature associated to each treatment (Label). Data grouped by label. Percentual standardization of values vs. t0. Negative differences greater than 10% are highlighted in red, while positive differences greater than 10% are highlighted in green.

	P-values	FDR	t0	CFD_t1	CRD_t1	MYC_t1	NFB_t1	NFB+MYC_t1	CFD_t2	CRD_t2	MYC_t2	NFB_t2	NFB+MYC_t2	LDA score	Notes
Abditibacterium	0.00126	0.00440	100.0	178.8	125.0	210.8	167.6	151.4	135.2	105.9	104.4	67.9	114.2	2.58	
Acidibacter	0.00018	0.00101	100.0	137.0	144.8	137.2	156.2	149.7	178.6	232.5	166.2	216.8	174.2	3.92	
Acidimicrobiia bacterium (uncultured)	0.00000	0.00002	100.0	56.5	67.6	54.0	59.6	60.9	51.1	43.4	46.3	45.5	46.6	4.07	
Acidobacteria bacterium (uncultured)	0.01339	0.02642	100.0	95.8	81.5	74.8	81.6	73.9	91.0	98.7	109.8	113.4	89.5	4.35	
Acidobacteriaceae bacterium (uncultured)	0.00227	0.00674	100.0	95.5	84.2	79.8	87.0	78.8	101.0	114.2	119.9	124.6	98.2	4.5	
Acidobacteriales bacterium (uncultured)	0.00013	0.00081	100.0	70.2	65.2	59.1	64.5	61.4	70.4	85.3	93.6	105.5	69.2	4.3	
Acidothermus	0.00004	0.00032	100.0	12.8	16.6	8.9	16.0	15.7	12.6	7.6	8.1	7.8	10.2	2.84	
Acidovorax	0.00000	0.00002	100.0	693.4	981.1	1022.7	797.0	303.4	358.1	158.8	250.2	178.0	277.6	3.44	
Actinobacterium (uncultured)	0.00002	0.00020	100.0	78.8	92.9	79.9	76.2	62.1	66.5	63.9	61.1	62.7	51.6	4.16	
Actinocorallia	0.00038	0.00182	100.0	61.9	91.4	82.1	77.4	43.1	44.3	62.6	38.7	72.0	28.3	3.03	
Actinomadura	0.00081	0.00303	100.0	77.6	75.5	84.7	81.7	101.9	64.6	67.7	61.2	74.6	55.4	2.83	
Actinomycetales bacterium (uncultured)	0.00000	0.00000	100.0	95.5	108.0	95.1	96.3	93.3	69.2	54.6	57.5	65.6	62.4	3.45	
Actinomycetospora	0.01895	0.03391	100.0	60.7	83.1	42.1	95.5	136.7	50.6	47.8	46.2	72.4	42.0	2.4	
Actinopolymorpha	0.03957	0.06041	100.0	43.8	51.5	43.5	44.3	72.8	45.3	23.3	43.5	40.4	34.5	2.44	
Actinotalea	0.03516	0.05463	100.0	90.5	87.4	91.8	77.2	112.3	66.6	53.2	59.9	52.8	79.5	2.53	
Adhaeribacter	0.00203	0.00625	100.0	122.4	108.8	169.4	156.3	171.5	106.9	105.7	110.9	100.3	142.4	3.72	
	**P-values**	**FDR**	**t0**	**CFD_t1**	**CRD_t1**	**MYC_t1**	**NFB_t1**	**NFB+MYC_t1**	**CFD_t2**	**CRD_t2**	**MYC_t2**	**NFB_t2**	**NFB+MYC_t2**	**LDA score**	**Notes**
Aeromicrobium	0.00395	0.01016	100.0	214.5	196.6	264.6	231.1	217.0	320.2	269.1	200.5	162.3	188.4	4.53	

Allorhizobium, Neorhizobium, Pararhizobium, Rhizobium	0.00127	0.00440	100.0	168.5	182.8	180.5	234.5	238.6	255.0	225.2	218.4	182.1	195.8	4.46	
Alsobacter	0.01812	0.03278	100.0	103.3	105.7	167.5	156.9	167.9	116.8	141.7	134.3	125.1	169.1	2.87	
Altererythrobacter	0.00068	0.00265	100.0	207.3	327.6	223.2	230.9	174.2	232.9	217.0	161.0	166.4	208.1	3.63	
Amaricoccus	0.00886	0.01862	100.0	109.9	127.4	144.3	158.4	227.3	124.6	113.0	114.7	124.9	144.7	3.44	
Aminobacter	0.00000	0.00001	100.0	200.0	151.7	788.1	242.0	654.6	186.4	277.3	308.3	245.4	282.2	3.47	
Ammoniphilus	0.00219	0.00665	100.0	71.5	98.9	82.5	77.2	134.0	80.6	89.6	61.5	66.4	74.0	3.11	
Amycolatopsis	0.00000	0.00000	100.0	367.8	253.9	261.5	107.3	70.3	2512.7	762.5	1085.4	1264.9	1574.9	3.63	
Anaerobacterium	0.01310	0.02594	100.0	34.2	35.6	43.3	30.0	42.2	21.7	28.8	26.3	27.3	29.8	2.75	
Anaerolinea	0.00142	0.00468	100.0	27.5	24.6	32.2	31.3	64.6	20.0	14.8	21.3	23.4	19.6	3.78	
Anaeromyxobacter	0.00344	0.00929	100.0	58.9	61.2	54.0	67.6	86.0	49.4	49.2	59.6	54.5	52.0	3.58	
Anaeromyxobacter dehalogenans	0.03063	0.04861	100.0	102.8	128.2	259.1	185.1	315.6	133.2	295.5	229.2	138.1	186.6	2.08	
Aquaspirillum arcticum group	0.02385	0.04018	100.0	179.2	201.9	393.1	438.0	371.9	291.2	280.0	184.8	217.1	232.8	2.15	
Aquicella	0.01029	0.02095	100.0	457.8	307.6	268.9	267.6	201.4	342.7	377.6	324.4	380.6	244.4	2.67	
Aquipuribacter	0.00986	0.02031	100.0	206.0	133.9	207.1	188.4	210.6	79.7	96.3	88.0	82.1	90.7	2.28	
Aquisphaera	0.02632	0.04322	100.0	50.6	60.9	47.2	50.3	66.6	46.5	38.2	56.9	52.2	37.1	2.82	
Arenimonas	0.00034	0.00170	100.0	268.3	309.2	256.7	240.9	231.8	297.9	224.4	219.3	125.2	243.1	3.45	
Aridibacter	0.00691	0.01510	100.0	172.1	132.0	166.0	176.1	159.9	147.8	144.5	123.0	139.8	169.0	3.18	
Armatimonadetes bacterium (uncultured)	0.00480	0.01163	100.0	107.3	105.8	135.5	119.3	121.0	79.1	94.2	91.8	93.9	87.2	3.16	
Armatimonas	0.00197	0.00614	100.0	608.8	1078.4	964.9	873.1	620.1	670.7	343.5	665.1	353.9	759.7	2.02	
Arthrobacter	0.00735	0.01597	100.0	109.8	89.0	130.5	119.5	128.7	126.8	108.9	132.8	108.5	195.3	5.42	

	**P-values**	**FDR**	**t0**	**CFD_t1**	**CRD_t1**	**MYC_t1**	**NFB_t1**	**NFB+MYC_t1**	**CFD_t2**	**CRD_t2**	**MYC_t2**	**NFB_t2**	**NFB+MYC_t2**	**LDA score**	**Notes**
Asanoa	0.02186	0.03769	100.0	47.9	119.6	63.0	68.6	121.1	78.1	57.2	100.1	86.1	71.0	2.25	
Asticcacaulis	0.00003	0.00027	100.0	392.7	1009.3	376.7	414.9	318.2	193.4	394.3	286.6	151.1	108.9	3.19	

Azohydromonas	0.00000	0.00000	100.0	155.7	145.4	176.6	161.2	184.5	323.0	324.4	274.8	273.4	322.9	3.46	
Azospira	0.00000	0.00004	100.0	6154.7	13346.5	11533.5	10667.7	4749.2	3313.8	2775.0	968.8	363.0	286.6	2.8	
Azospirillum	0.00428	0.01084	100.0	204.6	250.2	183.5	630.6	423.1	94.9	198.1	92.8	144.6	112.9	2.63	
Bacteroidetes bacterium (uncultured)	0.00342	0.00929	100.0	234.4	210.2	239.5	255.5	209.5	193.9	180.0	137.9	124.8	121.8	2.75	
Bauldia	0.01958	0.03465	100.0	150.8	220.2	135.8	212.4	148.8	145.4	260.3	140.9	133.9	103.0	3.22	
Bdellovibrio	0.00000	0.00001	100.0	267.2	291.0	304.6	300.0	307.4	171.9	212.5	151.1	151.6	219.1	3.02	
Blastocatella	0.00278	0.00802	100.0	164.9	140.4	181.0	168.3	139.5	139.0	129.3	134.1	147.5	145.5	3.49	
Blastococcus	0.00575	0.01326	100.0	108.5	105.0	136.8	132.9	152.9	96.2	105.0	91.9	98.2	115.4	4.14	
Blastopirellula	0.00005	0.00041	100.0	103.0	95.3	95.9	92.2	80.9	133.6	155.6	177.2	195.8	132.1	3.12	
Bosea	0.00137	0.00465	100.0	184.0	192.2	231.6	250.3	308.5	230.2	247.0	218.0	168.5	211.0	3.65	
Bradyrhizobium	0.00245	0.00714	100.0	129.6	153.5	118.7	147.0	133.1	154.0	162.8	163.4	178.8	147.7	3.94	
Brevibacillus	0.02128	0.03693	100.0	53.5	73.9	63.7	62.4	89.5	45.0	61.6	43.6	43.2	40.2	2.85	
Brevifollis	0.00001	0.00008	100.0	50.6	65.0	44.0	28.6	80.2	11.5	19.2	14.9	9.5	11.1	2.3	
Brevundimonas	0.00001	0.00016	100.0	115.8	145.3	158.7	113.3	121.5	84.4	52.2	35.5	31.1	78.2	2.78	
Burkholderia Caballeronia Paraburkholderia	0.02886	0.04623	100.0	51.7	74.0	58.5	56.4	90.4	73.5	71.0	34.3	31.2	40.2	3.43	
Burkholderiales bacterium Beta 02	0.00162	0.00509	100.0	600.6	374.3	347.3	357.0	295.6	499.1	385.6	290.0	244.6	308.9	2.27	
Caenimonas	0.00039	0.00184	100.0	164.2	146.4	209.1	205.1	219.8	210.1	198.1	159.3	150.5	213.5	4.02	
Caldicoprobacter	0.00498	0.01197	100.0	65.5	65.6	77.7	54.4	94.9	42.3	47.1	39.8	42.6	47.8	2.75	
Caldilineaceae bacterium (uncultured)	0.03295	0.05183	100.0	101.8	130.7	95.4	101.9	115.4	88.3	84.3	83.0	91.7	79.3	2.92	
	**P-values**	**FDR**	**t0**	**CFD_t1**	**CRD_t1**	**MYC_t1**	**NFB_t1**	**NFB+MYC_t1**	**CFD_t2**	**CRD_t2**	**MYC_t2**	**NFB_t2**	**NFB+MYC_t2**	**LDA score**	**Notes**
Candidatus Alysiosphaera	0.02903	0.04636	100.0	98.5	99.0	116.8	127.4	165.3	103.3	86.6	100.0	111.5	103.2	3.87	
Candidatus Chloroploca	0.02749	0.04487	100.0	139.2	185.2	172.2	226.8	267.9	230.3	134.9	252.4	212.5	205.1	2.86	

Candidatus Nomurabacteria bacterium GW2011 GWE1 32 28	0.02283	0.03888	100.0	275.3	211.7	305.4	267.1	304.0	126.6	230.7	76.4	76.7	70.6	2.08	
Candidatus Udaeobacter	0.00406	0.01038	100.0	97.4	91.3	60.0	67.5	43.1	84.6	97.5	98.2	117.0	75.4	4.18	
Candidatus Xiphinematobacter	0.00222	0.00669	100.0	90.6	72.0	72.0	78.8	65.3	97.9	116.2	130.2	150.2	96.1	4.71	
Caulobacter	0.00000	0.00001	100.0	162.1	439.1	176.8	176.5	138.8	84.6	79.1	60.1	46.9	63.6	3.63	
Cellulomonas	0.00006	0.00045	100.0	123.4	331.8	174.2	291.3	217.6	92.6	105.1	104.2	75.8	107.7	3.38	
Cellvibrio	0.00008	0.00055	100.0	252.2	588.6	270.6	217.7	260.3	103.9	92.6	71.9	50.4	86.9	3.64	
Chelativorans	0.00000	0.00000	100.0	116.7	191.7	5841.7	260.3	5895.1	159.4	133.0	1388.6	119.5	1191.2	3.57	
Chlamydomonas reinhardtii	0.01368	0.02679	100.0	813.0	695.1	1164.7	410.1	1612.6	384.9	770.1	391.9	415.2	788.6	2.27	
Chloroflexi bacterium (uncultured)	0.00000	0.00000	100.0	86.2	92.8	97.0	93.8	109.3	72.4	64.7	66.9	68.4	69.3	4.4	
Chloronema	0.00068	0.00265	100.0	246.9	539.4	639.2	578.3	509.7	311.8	368.5	758.5	591.9	553.9	3.05	
Chryseobacterium	0.02952	0.04699	100.0	71.6	135.2	24.4	26.6	18.7	106.4	57.8	183.0	107.1	196.9	2.42	
Chthoniobacter	0.03489	0.05439	100.0	146.8	121.3	154.1	152.0	138.6	128.5	142.1	134.9	127.1	129.6	4.1	
Chthonobacter	0.00444	0.01097	100.0	192.7	214.9	352.5	346.8	273.0	451.5	583.5	349.9	262.0	390.6	2.68	
Chthonomonas	0.00051	0.00222	100.0	132.3	146.2	150.7	141.0	158.1	107.1	118.1	111.8	129.0	139.4	2.95	
Clostridia bacterium (uncultured)	0.04274	0.06468	100.0	42.4	54.5	58.6	50.4	66.4	43.6	22.8	26.6	47.4	45.8	2.35	
Clostridium sensu stricto 1	0.00618	0.01394	100.0	61.5	57.5	47.2	54.8	86.3	42.9	28.6	52.0	48.6	40.5	3.27	
Clostridium sensu stricto 12	0.01778	0.03250	100.0	76.9	80.3	70.6	86.4	145.9	51.0	70.0	69.7	78.5	58.4	2.83	
Clostridium sensu stricto 13	0.01969	0.03465	100.0	92.8	87.7	70.9	77.7	124.2	55.6	63.8	58.7	75.1	65.2	3.06	
	**P-values**	**FDR**	**t0**	**CFD_t1**	**CRD_t1**	**MYC_t1**	**NFB_t1**	**NFB+MYC_t1**	**CFD_t2**	**CRD_t2**	**MYC_t2**	**NFB_t2**	**NFB+MYC_t2**	**LDA score**	**Notes**
Clostridium sensu stricto 8	0.00443	0.01097	100.0	64.5	66.8	64.5	62.2	100.9	48.5	49.2	53.3	57.5	52.2	2.94	
Cnuella	0.00159	0.00502	100.0	310.9	265.9	389.6	251.2	368.3	232.4	282.7	194.8	226.2	289.0	2.65	
Cohnella	0.00057	0.00237	100.0	60.8	76.4	73.5	75.7	110.4	44.9	52.5	53.4	56.7	53.3	3.61	

Comamonas	0.00001	0.00011	100.0	198.2	194.5	200.6	204.7	205.0	419.4	362.1	327.6	315.0	331.5	3.1	
Conexibacter	0.00036	0.00177	100.0	94.9	108.6	109.0	102.1	100.7	85.3	61.1	60.9	64.6	75.9	3.23	
Conexibacteraceae bacterium (uncultured)	0.00043	0.00196	100.0	63.3	70.9	72.8	71.8	41.0	51.2	29.9	38.9	51.6	40.5	2.49	
Croceicoccus	0.02866	0.04605	100.0	376.9	674.9	623.6	564.0	486.8	472.7	539.3	398.4	360.0	496.0	2.5	
Cupriavidus	0.00001	0.00011	100.0	141.9	151.0	115.8	146.7	301.9	238.3	477.1	274.4	274.2	306.7	3.59	
Dactylosporangium	0.00047	0.00210	100.0	60.5	77.0	60.9	65.6	117.4	61.9	44.5	49.1	66.8	49.3	2.82	
Deinococcus	0.00000	0.00001	100.0	4685.1	3189.4	4167.6	2377.2	5324.9	94418.0	58495.0	25477.5	13930.1	28719.4	3.53	
delta proteobacterium WX81	0.03074	0.04864	100.0	60.3	41.5	48.4	42.7	53.3	54.7	55.1	62.5	68.0	42.9	2.3	
Deltaproteobacteria bacterium GWC2 55 46	0.00375	0.00988	100.0	28.6	25.7	32.6	32.5	71.2	25.7	16.7	20.1	26.7	20.0	2.5	
Desmochloris halophila	0.00047	0.00210	100.0	4741.0	2550.6	2899.1	2417.0	2990.7	2030.3	2086.0	1820.0	2133.4	2118.6	3.36	
Desulfohalotomaculum	0.01215	0.02424	100.0	57.6	75.8	64.7	91.3	146.0	44.3	49.0	42.5	63.2	42.3	2.11	
Desulfosporosinus	0.00586	0.01341	100.0	38.0	45.5	42.6	51.0	74.8	27.2	37.0	31.4	28.3	33.7	2.35	
Devosia	0.00000	0.00007	100.0	221.3	300.8	221.0	249.9	204.5	244.0	251.8	174.7	157.0	158.3	4.27	
Dokdonella	0.00003	0.00029	100.0	303260.0	773930.0	409510.0	866500.0	159150.0	214740.0	590340.0	203650.0	213420.0	321000.0	2.64	
Domibacillus	0.00066	0.00260	100.0	92.1	75.5	73.1	73.7	85.5	203.6	132.7	180.8	156.2	290.2	3.49	
Dongia	0.00993	0.02038	100.0	79.0	72.6	58.2	64.2	61.6	68.5	86.4	77.7	89.5	71.6	3.64	
Dyadobacter	0.00011	0.00069	100.0	214.2	270.5	236.8	179.6	105.7	559.8	208.9	635.9	177.2	268.8	3.9	
Ellin517	0.00066	0.00261	100.0	313.4	225.3	218.3	210.4	144.4	221.7	270.5	216.3	219.2	212.3	3.87	
Ellin6055	0.00009	0.00063	100.0	195.9	167.0	197.4	185.6	163.3	147.2	131.7	112.8	110.1	151.0	4.05	
Ellin6067	0.01801	0.03278	100.0	160.2	151.0	177.2	187.3	136.8	223.0	247.3	181.9	145.3	184.1	3.86	
	**P-values**	**FDR**	**t0**	**CFD_t1**	**CRD_t1**	**MYC_t1**	**NFB_t1**	**NFB+MYC_t1**	**CFD_t2**	**CRD_t2**	**MYC_t2**	**NFB_t2**	**NFB+MYC_t2**	**LDA score**	**Notes**
Ensifer	0.00000	0.00002	100.0	153.9	142.2	146.8	137.6	146.3	202.3	210.6	233.1	216.4	211.0	3.85	
Enterobacter	0.00443	0.01097	100.0	503.5	65.4	68.1	53.0	71.1	2278.7	393.4	443.8	401.8	2091.9	4.18	
Erythrobacter	0.00076	0.00286	100.0	2774.2	5218.2	11342.6	2716.1	1854.9	4715.5	3074.8	2308.0	3126.7	5781.0	3.1	
Ettlia pseudoalveolaris	0.01813	0.03278	100.0	596.9	508.4	930.0	726.4	1170.0	753.8	510.0	406.8	319.3	3129.0	3.23	

Ferrovibrio	0.02576	0.04266	100.0	327.2	284.6	339.3	361.9	321.3	303.8	318.5	181.0	279.5	206.7	2.47	
Ferruginibacter	0.00557	0.01292	100.0	142.8	120.3	160.0	165.0	157.9	117.1	117.2	118.7	112.5	129.7	3.37	
Fervidicella	0.00681	0.01505	100.0	69.2	42.1	65.0	30.1	161.2	12.3	34.6	23.9	49.4	38.5	2.44	
Fictibacillus	0.00004	0.00035	100.0	56.7	60.8	60.3	58.3	79.9	37.2	72.1	37.2	46.9	50.5	3.77	
Fimbriiglobus	0.00031	0.00162	100.0	134.4	129.1	119.7	130.3	109.8	145.6	161.3	172.1	176.8	146.2	3.86	
Flavihumibacter	0.00853	0.01806	100.0	154.4	135.6	203.9	202.3	206.5	218.3	244.5	239.7	254.2	285.1	2.42	
Flavisolibacter	0.00140	0.00468	100.0	159.2	134.7	184.7	172.9	164.3	143.1	158.5	130.7	126.9	146.9	3.86	
Flavitalea	0.00000	0.00001	100.0	193.6	174.1	240.6	232.1	188.8	238.0	309.3	246.9	239.7	269.0	3.4	
Flavobacterium	0.01213	0.02424	100.0	313.8	398.1	277.1	170.8	279.4	383.7	223.6	218.6	126.4	176.4	3.35	
Frankineae bacterium (uncultured)	0.00831	0.01775	100.0	70.6	93.0	126.9	86.7	148.6	70.5	62.7	56.7	65.4	56.8	2.25	
Gaiella	0.00000	0.00000	100.0	68.4	80.3	75.0	71.3	79.4	51.6	42.6	45.8	48.5	46.2	5	
Galbitalea	0.01487	0.02869	100.0	99.9	127.0	95.2	115.2	105.5	96.2	93.2	57.7	55.8	66.8	3.34	
Gemmata	0.00026	0.00142	100.0	118.0	109.3	90.2	93.2	78.0	114.2	127.3	128.9	136.1	109.9	4.5	
Gemmatimonadales bacterium (uncultured)	0.03593	0.05566	100.0	82.2	70.1	51.3	56.8	48.0	65.4	74.9	78.0	90.1	69.9	3.34	
Gemmatimonas	0.00026	0.00143	100.0	175.0	144.8	213.9	212.7	201.5	186.9	211.8	168.2	150.6	183.1	3.92	
Gemmatirosa	0.01892	0.03391	100.0	165.6	160.0	184.2	149.0	185.1	112.7	78.9	88.3	59.2	118.8	2.06	
Geoalkalibacter	0.00200	0.00618	100.0	11.9	12.7	25.7	22.0	31.5	10.8	7.7	12.2	17.8	13.7	2.99	
Glycomyces	0.00596	0.01356	100.0	174.8	170.0	77.5	116.6	85.1	530.7	137.3	204.8	171.9	208.4	2.57	
Halobacillus	0.00000	0.00000	100.0	14622.7	5850.0	23569.2	27249.9	2667.5	375.0	668.8	407.6	754.2	594.3	3.51	

	P-values	FDR	t0	CFD_t1	CRD_t1	MYC_t1	NFB_t1	NFB+MYC_t1	CFD_t2	CRD_t2	MYC_t2	NFB_t2	NFB+MYC_t2	LDA score	Notes
Hassallia	0.00000	0.00004	100.0	419.0	862.2	492.8	951.8	585.5	158.8	234.4	127.0	110.1	77.8	3.61	
Herbinix	0.00233	0.00684	100.0	59.3	68.2	59.6	68.8	93.7	45.2	45.0	40.4	48.1	53.8	2.85	
Herpetosiphon	0.01505	0.02893	100.0	161.9	174.7	213.3	167.2	149.3	205.6	144.3	140.4	127.4	171.7	3.86	
Hirschia	0.00367	0.00982	100.0	312.6	452.8	357.6	592.3	377.4	349.2	1050.6	387.8	287.1	310.1	3.72	
Hungateiclostridium	0.03272	0.05161	100.0	51.2	56.2	86.2	68.6	102.2	41.5	53.6	32.2	42.8	99.1	2.01	

Hyaloperonospora arabidopsidis	0.00001	0.00008	100.0	495.2	407.7	406.0	322.0	354.8	1151.2	900.2	791.5	955.4	708.3	2.4	
Hymenobacter	0.00542	0.01270	100.0	81.9	123.0	253.8	175.5	227.2	78.8	94.3	103.3	75.7	158.8	2.34	
Iamia	0.01779	0.03250	100.0	96.3	106.6	95.9	96.9	79.5	105.6	81.2	70.1	72.0	70.8	3.64	
Ideonella	0.00911	0.01899	100.0	354.7	876.4	387.9	485.0	279.2	478.7	571.8	565.5	418.5	447.3	3.06	
Ilumatobacter	0.01706	0.03174	100.0	80.0	99.5	73.2	75.6	70.6	78.5	51.9	64.3	62.4	62.5	3.44	
Inquilinus	0.00425	0.01080	100.0	228.4	272.2	153.9	168.0	364.8	133.6	503.8	233.9	178.1	190.8	2.71	
Jahnella	0.00789	0.01691	100.0	48.5	119.9	86.3	187.3	98.8	116.0	1147.7	1081.6	98.2	277.4	2.87	
Knoellia	0.00028	0.00150	100.0	90.3	86.2	96.4	100.6	109.2	81.8	70.8	71.1	70.9	86.9	4.3	
Kribbella	0.01562	0.02987	100.0	70.8	82.3	70.5	73.1	74.4	84.6	70.7	75.5	74.0	70.3	2.91	
Lacibacter	0.01648	0.03088	100.0	237.9	181.0	225.7	160.2	134.4	261.7	190.9	179.1	155.0	219.6	3.28	
Lacunisphaera	0.00008	0.00054	100.0	208.7	353.3	251.6	258.7	193.8	131.3	206.3	77.1	88.8	60.3	3.27	
Lapillicoccus	0.01963	0.03465	100.0	96.9	120.1	138.0	134.0	151.8	100.5	68.8	87.0	79.3	126.5	2.47	
Lechevalieria	0.00000	0.00002	100.0	171.9	187.1	119.5	127.4	97.0	332.7	211.5	181.8	219.9	208.3	3.91	
Legionella	0.00008	0.00054	100.0	261.4	346.2	263.5	290.1	284.3	204.7	231.0	223.3	193.0	181.2	3.07	
Leptolyngbya EcFYyyy 00	0.00009	0.00063	100.0	2778.2	5318.0	9853.4	10869.5	8977.7	2012.7	2617.7	5671.3	5941.9	3923.0	4.61	
Longimicrobium	0.00692	0.01510	100.0	291.7	252.3	410.9	365.7	404.6	252.8	536.6	584.2	413.7	385.0	2.98	
Luedemannella	0.00003	0.00027	100.0	44.6	62.0	48.8	49.7	81.7	44.7	36.6	42.3	45.5	37.0	3.47	
Luteimonas	0.00146	0.00479	100.0	261.2	351.3	143.0	217.1	127.0	407.8	269.4	197.5	174.4	167.5	2.88	
Luteolibacter	0.00907	0.01899	100.0	213.7	272.1	213.2	221.9	176.7	572.5	479.1	347.3	367.4	232.5	4.23	
	**P-values**	**FDR**	**t0**	**CFD_t1**	**CRD_t1**	**MYC_t1**	**NFB_t1**	**NFB+MYC_t1**	**CFD_t2**	**CRD_t2**	**MYC_t2**	**NFB_t2**	**NFB+MYC_t2**	**LDA score**	**Notes**
Lysinimonas	0.00915	0.01900	100.0	158.4	183.5	182.1	138.8	159.9	157.8	106.8	114.1	87.2	116.0	2.71	
Lysobacter	0.00463	0.01133	100.0	140.9	129.5	161.6	138.9	136.8	158.9	136.5	111.0	101.3	135.3	3.9	
Marisediminicola	0.01599	0.03026	100.0	70.5	124.0	161.5	137.1	212.7	68.7	69.6	81.8	50.6	101.8	2.18	
Marmoricola	0.00516	0.01234	100.0	79.8	73.2	82.8	81.4	85.3	76.1	83.2	66.5	64.4	77.5	4.1	
Massilia	0.01029	0.02095	100.0	99.8	95.0	132.7	126.8	151.3	106.6	111.0	96.9	74.9	134.2	4.26	
Meiothermus	0.01882	0.03391	100.0	33673.0	13085.2	9512.7	31131.6	14948.1	8563.4	10204.6	12261.9	20712.2	8951.0	2.62	

Mesorhizobium	0.00033	0.00168	100.0	189.2	287.6	217.8	261.6	225.2	217.7	274.8	208.3	208.4	217.3	4.14	
Methylobacillus	0.00000	0.00000	100.0	1728.5	1857.7	1914.4	525.7	766.5	10952.0	3194.7	5461.8	2528.7	5780.6	3.42	
Methyloceanibacter	0.02824	0.04566	100.0	68.6	67.8	60.3	59.1	47.4	56.8	66.9	80.9	75.8	52.0	2.21	
Methylocella	0.00140	0.00468	100.0	193.4	332.7	292.3	387.0	281.7	109.5	127.7	107.9	109.5	148.9	2.38	
Methylopila	0.01773	0.03250	100.0	129.9	429.1	231.9	200.8	147.8	101.0	534.5	293.5	130.8	330.5	2.66	
Methylorosula	0.00343	0.00929	100.0	133.7	129.3	180.2	139.7	168.2	100.4	113.8	95.0	96.4	96.7	2.58	
Methylotenera	0.00001	0.00008	100.0	335.1	346.3	382.1	292.9	268.7	1367.8	495.0	658.2	455.0	1510.1	3.82	
Microbacterium	0.00156	0.00502	100.0	184.3	157.4	157.2	127.8	107.6	362.8	219.5	196.2	135.6	223.5	3.96	
Micropepsis	0.00002	0.00024	100.0	10269.2	22658.1	8319.3	19952.1	9733.9	5676.5	20068.0	7861.8	5113.0	2433.7	2.86	
Microvirga	0.00292	0.00823	100.0	112.9	97.4	148.3	142.4	147.8	130.9	167.2	131.8	140.2	151.3	4.58	
Mitsuaria	0.00000	0.00002	100.0	2466.2	4513.6	1598.9	2659.9	1282.3	15413.7	3360.5	19314.9	7878.3	4203.8	4.11	
Mucilaginibacter	0.00012	0.00075	100.0	65.8	77.5	82.4	86.2	94.9	33.4	49.4	21.1	31.7	13.1	2.77	
Mycobacterium	0.00000	0.00004	100.0	80.9	96.1	86.7	89.4	89.2	74.5	87.4	68.7	75.1	68.7	3.78	
Nakamurella	0.02125	0.03693	100.0	81.0	92.4	109.6	124.1	158.1	99.2	86.1	98.4	94.3	110.5	2.72	
Nannocystis	0.01458	0.02833	100.0	129.0	162.5	215.5	271.2	171.9	204.1	251.1	158.7	209.4	151.6	2.55	
Niastella	0.00004	0.00035	100.0	187.8	197.6	171.6	229.1	127.8	257.0	243.6	244.1	236.3	205.7	3.24	
Nibribacter	0.00774	0.01672	100.0	121.9	82.9	184.9	152.7	151.4	128.1	81.3	74.2	54.5	89.8	2.72	
Nitratireductor	0.01276	0.02537	100.0	186.4	178.4	246.6	180.2	153.1	172.3	135.1	175.7	178.8	207.5	2.48	
Nitrosomonas	0.00033	0.00168	100.0	533.4	430.2	568.0	711.3	648.7	301.0	402.0	487.1	413.6	384.2	2.94	

	P-values	FDR	t0	CFD_t1	CRD_t1	MYC_t1	NFB_t1	NFB+MYC_t1	CFD_t2	CRD_t2	MYC_t2	NFB_t2	NFB+MYC_t2	LDA score	Notes
Nitrosospira	0.03976	0.06052	100.0	172.5	193.4	201.5	303.0	278.1	107.9	122.8	162.2	139.7	114.9	3.09	
Nocardioides	0.00014	0.00084	100.0	86.9	91.6	89.3	93.3	82.6	79.0	59.0	60.7	61.2	71.6	4.31	
Nodosilinea PCC 7104	0.00011	0.00069	100.0	2166.3	7844.0	3555.7	4166.3	11153.1	6097.6	3849.3	6738.2	5521.4	9557.8	3.85	
Nonomuraea	0.00531	0.01258	100.0	63.4	80.3	83.9	75.8	119.1	60.3	50.7	66.0	68.0	55.9	3.13	
Nordella	0.00009	0.00063	100.0	130.0	112.6	99.7	110.6	87.5	121.3	148.5	141.8	160.8	123.2	3.96	
Nostoc PCC 7524	0.01177	0.02368	100.0	223.1	236.9	160.0	133.7	200.6	72.9	50.2	160.9	77.5	110.8	2.51	
Noviherbaspirillum	0.00380	0.00997	100.0	139.9	119.6	183.1	166.0	166.9	162.5	157.0	152.8	124.5	198.2	3.97	

Novosphingobium	0.00157	0.00502	100.0	84.8	121.8	94.0	84.2	72.7	66.9	51.7	37.6	44.0	64.3	3.14	
Ohtaekwangia	0.02306	0.03899	100.0	210.5	246.8	208.5	181.7	176.8	206.2	210.3	165.1	161.3	178.1	3.68	
Oligoflexus	0.03917	0.05997	100.0	112.7	115.1	189.4	143.0	183.8	102.1	99.6	100.7	103.1	134.1	3.05	
Oscillatoria SAG 1459 8	0.00208	0.00634	100.0	2105.7	3906.4	3585.8	4794.1	2901.7	2745.1	2471.0	4363.6	5013.7	6140.7	2.65	
Oscillochloris	0.01618	0.03042	100.0	325.3	400.2	352.5	238.7	315.7	438.0	303.6	285.5	327.6	341.5	2.35	
Paenarthrobacter	0.00150	0.00491	100.0	128.9	75.6	101.5	87.4	88.9	218.2	107.2	162.9	126.9	214.1	3.75	
Paenisporosarcina	0.01173	0.02368	100.0	82.8	77.0	76.9	69.3	106.3	49.4	71.9	54.4	63.8	66.2	3.5	
Panacagrimonas	0.00363	0.00975	100.0	470.7	464.5	599.9	442.7	320.0	437.9	266.2	397.7	209.4	293.3	2.8	
Parasegetibacter	0.00005	0.00036	100.0	203.7	214.6	289.8	229.5	308.6	196.8	173.1	205.2	153.7	265.7	3.27	
Parviterribacter	0.00672	0.01496	100.0	93.3	96.3	102.8	97.3	98.6	73.8	68.3	61.4	59.8	64.5	3.06	
Paucibacter	0.00958	0.01982	100.0	282.8	202.3	260.8	208.0	237.1	216.8	221.8	188.7	245.1	244.4	2.71	
Pedinomonas minor	0.02292	0.03888	100.0	133.4	135.5	253.3	246.1	256.4	86.7	77.4	88.2	121.8	109.2	2.52	
Pedobacter	0.00003	0.00027	100.0	146.3	128.1	173.0	159.0	188.5	103.6	102.5	93.9	90.1	115.7	3.62	
Pedococcus Phycicoccus	0.00101	0.00368	100.0	88.8	81.0	81.0	83.7	69.4	78.1	48.0	52.4	60.3	73.3	2.93	
Pedomicrobium	0.00016	0.00090	100.0	71.4	74.2	67.3	72.9	70.9	85.2	98.9	101.5	109.1	77.8	3.78	
Pelomonas	0.00023	0.00125	100.0	443.4	706.9	536.4	380.4	435.7	423.4	196.1	172.2	199.1	342.7	2.86	
Pelosinus	0.00030	0.00155	100.0	50.6	50.6	46.8	45.4	73.0	37.9	44.1	36.5	46.3	32.9	2.89	
Peredibacter	0.00041	0.00193	100.0	193.6	165.0	206.5	217.4	205.1	174.1	164.9	150.7	128.6	149.1	3.04	

	**P-values**	**FDR**	**t0**	**CFD_t1**	**CRD_t1**	**MYC_t1**	**NFB_t1**	**NFB+MYC_t1**	**CFD_t2**	**CRD_t2**	**MYC_t2**	**NFB_t2**	**NFB+MYC_t2**	**LDA score**	**Notes**
Phenylobacterium	0.00000	0.00001	100.0	89.2	117.7	110.2	98.2	105.5	67.3	66.7	59.7	53.9	76.4	3.08	
Pirellula	0.00055	0.00235	100.0	134.2	114.1	100.7	104.0	72.4	135.2	143.7	139.9	140.5	115.0	4.57	
Piscinibacter	0.00093	0.00340	100.0	203.6	181.3	198.4	154.2	197.2	323.7	230.7	247.1	268.0	336.4	2.75	
Planctomicrobium	0.00141	0.00468	100.0	187.0	152.9	129.5	115.3	80.1	264.5	214.7	200.0	195.2	153.4	2.9	
Planctomycetaceae bacterium (uncultured)	0.00291	0.00823	100.0	160.9	147.7	144.9	140.5	85.5	41.4	41.9	87.5	75.8	39.9	2.05	
Planctomycetales bacterium (uncultured)	0.00001	0.00015	100.0	151.0	182.3	153.2	156.6	89.8	179.0	212.0	181.3	210.1	145.6	3.11	

	P-values	FDR	t0	CFD_t1	CRD_t1	MYC_t1	NFB_t1	NFB+MYC_t1	CFD_t2	CRD_t2	MYC_t2	NFB_t2	NFB+MYC_t2	LDA score	Notes
Planctomycete (uncultured)	0.00005	0.00041	100.0	186.5	140.6	179.9	178.4	148.1	212.4	231.5	187.4	181.8	176.8	4.31	
Planctomycete WY108	0.00543	0.01270	100.0	356.9	204.3	437.9	294.5	269.6	373.8	310.8	290.5	333.5	369.8	2.16	
Planctopirus	0.00001	0.00013	100.0	433.9	557.9	324.5	584.8	354.2	309.7	487.0	265.9	257.6	259.2	3.4	
Planifilum	0.02763	0.04495	100.0	35.9	72.9	57.6	49.9	82.4	25.9	31.5	35.3	26.8	32.4	2.96	
Planoglabratella opercularis	0.00541	0.01270	100.0	519.7	493.1	674.2	789.3	631.4	382.8	328.4	409.4	456.0	491.4	2.56	
Polyangiaceae bacterium (uncultured)	0.00321	0.00890	100.0	353.9	180.3	273.3	182.8	172.0	465.9	706.6	447.1	478.4	259.6	2.29	
Polyangium brachysporum group	0.00000	0.00000	100.0	295.3	253.4	342.6	257.1	177.0	713.1	829.1	387.3	528.6	521.7	3.66	
Polycyclovorans	0.02839	0.04576	100.0	140.9	180.3	182.2	135.1	180.8	87.0	73.0	92.7	53.7	29.2	3.39	
Pontibacter	0.00072	0.00276	100.0	175.7	150.0	232.9	213.5	258.8	152.8	129.7	131.3	122.3	147.9	3.72	
Promicromonospora	0.01565	0.02987	100.0	52.0	81.1	38.9	51.5	61.7	88.7	102.8	58.8	45.6	48.6	3.19	
Prosthecomicrobium	0.00299	0.00839	100.0	246.5	161.4	249.5	242.9	259.7	330.6	289.9	175.2	216.9	288.0	2.46	
Pseudarthrobacter	0.02186	0.03769	100.0	127.8	97.5	168.4	125.9	139.1	153.4	109.8	143.1	115.5	212.4	3.4	
Pseudenhygromyxa	0.03489	0.05439	100.0	246.2	317.3	331.7	335.2	418.8	91.4	183.1	141.5	162.3	104.0	2.36	
Pseudoduganella	0.00011	0.00069	100.0	697.2	574.8	1151.0	509.4	287.1	848.1	680.5	813.8	535.6	386.6	3.5	
Pseudoflavitalea	0.00776	0.01672	100.0	150.4	168.7	116.8	160.3	202.2	88.1	271.7	161.6	192.3	119.4	3.16	
Pseudolabrys	0.00116	0.00412	100.0	147.1	240.0	107.5	190.3	108.3	94.5	104.3	87.0	81.4	74.2	3.96	
Pseudomuriella schumacherensis	0.00011	0.00069	100.0	1103.7	1005.4	1172.7	1107.3	1058.5	551.6	906.8	1067.5	1722.7	1600.4	3.45	
Pseudonocardia	0.00687	0.01510	100.0	85.4	94.6	100.8	96.5	124.4	80.3	73.3	76.5	96.0	76.9	3.12	
Pseudorhodoplanes	0.00166	0.00519	100.0	144.3	157.5	131.0	181.3	111.9	123.7	186.0	156.1	166.4	135.3	3.02	
Pseudoxanthomonas	0.00079	0.00298	100.0	206.5	337.5	189.8	212.4	163.8	436.4	329.8	317.1	195.7	257.6	3.92	
Qipengyuania	0.00000	0.00001	100.0	295.9	253.6	334.0	250.2	235.4	181.2	116.9	121.6	112.1	182.1	3.74	
Ramlibacter	0.00027	0.00143	100.0	170.4	147.9	220.8	208.4	204.9	243.6	248.5	202.1	176.8	223.4	4.02	
Reyranella	0.00109	0.00391	100.0	146.8	174.9	128.0	139.9	118.1	141.6	182.0	172.4	171.2	128.2	3.52	

	P-values	FDR	t0	CFD_t1	CRD_t1	MYC_t1	NFB_t1	NFB+MYC_t1	CFD_t2	CRD_t2	MYC_t2	NFB_t2	NFB+MYC_t2	LDA score	Notes
Rhizobacter	0.00054	0.00233	100.0	172.6	192.9	191.2	180.3	193.2	220.7	195.7	179.3	169.4	213.3	3.89	
Rhodanobacter	0.00000	0.00000	100.0	9761.9	30706.6	9509.4	21582.3	13454.3	2603.8	7776.6	2537.6	2263.4	1794.9	3.55	
Rhodobacter	0.00003	0.00029	100.0	220.0	331.4	410.8	396.6	389.1	204.2	121.8	138.0	141.4	222.2	3.37	
Rhodococcus	0.02581	0.04266	100.0	64.5	41.3	44.6	57.2	54.4	70.9	72.9	40.9	47.8	65.2	2.88	
Rhodocytophaga	0.01730	0.03195	100.0	194.3	173.3	215.8	209.7	243.1	198.8	195.1	235.6	179.9	229.3	3.17	
Rhodoferax	0.00003	0.00029	100.0	197.8	335.0	207.9	341.9	291.2	108.5	117.8	95.5	78.9	105.0	2.85	
Rhodopirellula	0.00523	0.01246	100.0	193.4	162.7	201.1	197.7	174.6	188.7	215.2	158.6	165.1	167.7	3.51	
Rhodoplanes	0.00246	0.00714	100.0	125.3	116.3	95.4	108.4	82.3	125.7	132.7	146.4	155.0	112.1	3.66	
Rhodopseudomonas	0.00001	0.00011	100.0	914.0	1710.7	1055.1	1891.1	1275.6	620.6	1217.0	487.3	575.8	674.0	2.85	
Roseisolibacter	0.00233	0.00684	100.0	123.1	169.5	173.8	147.5	189.3	123.0	131.4	112.0	82.4	118.3	2.67	
Roseomonas	0.00651	0.01456	100.0	145.9	134.3	196.2	197.2	218.9	154.1	169.9	150.4	143.4	169.6	3.07	
Rubellimicrobium	0.00020	0.00112	100.0	183.4	153.6	264.0	256.0	281.9	192.8	169.4	167.6	146.1	211.1	3.96	
Rubrobacter	0.01717	0.03182	100.0	92.7	97.9	115.1	115.4	125.7	79.0	85.7	84.9	91.1	92.2	4.48	
Rubrobacterales bacterium (uncultured)	0.00000	0.00000	100.0	70.2	78.7	65.2	67.6	62.6	47.9	39.5	39.5	45.3	40.5	4.35	
Rubrobacteria bacterium (uncultured)	0.00000	0.00000	100.0	74.5	91.4	79.5	76.8	80.2	55.6	46.3	49.6	54.0	50.5	3.86	
Ruminiclostridium	0.00036	0.00177	100.0	37.0	46.4	55.4	49.8	84.7	34.1	37.6	39.0	46.3	37.3	3.1	
Saccharothrix	0.01654	0.03088	100.0	174.9	129.1	137.6	124.2	178.2	121.2	80.1	85.5	74.1	101.9	3	
Salinispora	0.00156	0.00502	100.0	60.5	66.8	59.3	71.7	67.1	37.1	43.8	48.5	72.5	39.5	2.55	
Sandaracinus	0.01947	0.03461	100.0	110.8	125.4	135.7	109.5	118.6	79.3	105.0	83.7	87.3	68.8	2.87	
Schlesneria	0.00001	0.00013	100.0	3464.1	5393.5	3999.1	6741.6	1920.2	1751.6	1279.8	858.2	757.1	1006.1	2.53	
Segetibacter	0.00109	0.00391	100.0	125.2	76.1	121.8	125.5	101.7	69.1	54.8	53.4	60.2	79.4	2.76	
Shimazuella	0.00369	0.00983	100.0	59.2	83.4	77.5	78.8	124.0	64.5	54.9	69.8	60.1	58.4	3.02	
Shinella	0.00006	0.00047	100.0	631.8	999.5	475.5	417.4	319.8	760.7	422.1	588.2	416.7	374.1	3.67	
Sideroxydans	0.00040	0.00187	100.0	4.1	6.8	8.9	9.3	12.3	2.4	2.1	5.3	12.4	1.9	3.21	

Simplicispira	0.00000	0.00001	100.0	1723.0	4523.0	1524.9	3452.8	1911.5	326.3	933.5	374.4	196.2	160.8	2.86	
Skermanella	0.02010	0.03512	100.0	93.6	102.4	127.0	149.9	193.7	99.3	98.2	101.5	119.0	124.0	4.42	
Solibacillus	0.02247	0.03851	100.0	47.7	75.2	64.7	59.9	89.8	60.7	58.8	51.1	35.8	70.0	2.25	
Solimonas	0.00006	0.00042	100.0	352.6	659.8	387.0	257.2	237.5	114.3	210.3	63.7	60.6	40.5	2.76	
Solirubrobacter	0.00002	0.00020	100.0	77.6	85.5	84.2	83.1	86.7	60.5	56.6	56.5	57.3	66.1	3.98	
Sorangium	0.00128	0.00440	100.0	49.2	89.5	59.1	111.2	48.0	93.6	125.5	75.1	54.8	76.5	3.1	
Sphingoaurantiacus	0.00002	0.00024	100.0	134.6	132.0	186.0	163.0	195.6	125.5	114.1	90.9	97.9	159.0	3	
Sphingobium	0.00000	0.00004	100.0	1311.4	1537.7	1944.1	1358.1	954.5	3479.8	1751.6	1827.4	1209.7	1978.9	4.63	
Sphingomonas	0.00001	0.00014	100.0	114.1	115.5	130.8	124.6	117.4	101.3	98.1	80.3	80.5	101.2	4.59	
Sphingopyxis	0.00000	0.00000	100.0	400.2	758.2	416.6	415.7	257.0	374.6	165.2	167.0	146.0	167.3	3.72	
Spirochaeta	0.00085	0.00314	100.0	12.0	8.4	28.0	15.9	8.8	8.8	8.4	7.2	8.3	14.2	2.91	
Sporosarcina	0.00060	0.00244	100.0	72.6	75.1	89.1	73.8	110.9	50.5	53.8	55.2	62.1	70.9	3.08	
Stenotrophomonas	0.02444	0.04093	100.0	67.8	50.2	11.8	51.9	9.7	157.2	68.7	47.4	24.5	10.7	2.78	
Steroidobacter	0.00120	0.00425	100.0	78.1	90.0	79.7	87.0	99.4	104.6	124.1	125.5	135.4	112.4	3.82	
	**P-values**	**FDR**	**t0**	**CFD_t1**	**CRD_t1**	**MYC_t1**	**NFB_t1**	**NFB+MYC_t1**	**CFD_t2**	**CRD_t2**	**MYC_t2**	**NFB_t2**	**NFB+MYC_t2**	**LDA score**	**Notes**
Streptomyces	0.00042	0.00194	100.0	136.9	139.0	105.7	110.3	113.2	162.1	141.4	118.4	135.6	143.3	4.12	
Streptosporangium	0.02289	0.03888	100.0	76.0	111.5	71.7	121.8	137.0	45.9	84.3	59.4	61.4	69.6	2.49	
Symbiobacterium	0.00616	0.01394	100.0	54.0	72.5	61.1	57.8	84.2	35.2	30.7	41.3	38.3	51.2	2.77	
Synechococcus IR11	0.00009	0.00063	100.0	2082.0	8533.3	6948.1	7753.9	9440.2	3334.8	9992.6	3578.0	10109.7	11642.5	3.41	
Syntrophobacter	0.02445	0.04093	100.0	40.6	24.5	34.6	30.9	46.7	24.9	25.2	23.9	28.8	27.5	2.77	
Tahibacter	0.01475	0.02857	100.0	322.7	247.1	197.4	684.3	371.7	460.7	498.4	240.6	177.9	220.4	3.16	
Telmatocola	0.04084	0.06197	100.0	156.3	97.6	193.8	124.1	173.2	130.0	126.1	84.8	105.7	98.5	2.39	
Tepidisphaera	0.01603	0.03026	100.0	197.4	149.5	232.1	214.9	160.4	180.1	192.4	171.4	132.7	136.9	3.21	
Terribacillus	0.00621	0.01395	100.0	62.3	86.6	81.1	65.3	112.2	49.6	73.8	65.4	80.6	235.4	3.01	
Terrimicrobium	0.00022	0.00122	100.0	193.8	317.1	194.4	282.2	282.4	439.4	338.2	329.2	323.0	249.1	2.82	
Terrimonas	0.00018	0.00105	100.0	174.7	161.8	157.9	176.5	126.6	204.1	254.7	202.8	205.5	194.9	3.87	
Thermoactinomyces	0.03773	0.05811	100.0	39.4	65.5	55.0	42.8	61.9	29.2	41.7	43.2	33.5	27.1	2.72	

Thermoflavimicrobium	0.00439	0.01097	100.0	38.8	81.3	65.3	50.3	115.4	40.4	46.2	48.8	51.7	43.2	2.73	
Truepera	0.00000	0.00000	100.0	251.6	168.5	263.3	166.7	240.4	726.0	997.2	855.5	569.4	1004.8	3.5	
Tumebacillus	0.00057	0.00238	100.0	45.6	89.5	53.7	61.5	100.6	34.7	27.7	44.4	41.0	33.5	3.76	
Tychonema CCAP 1459 11B	0.01920	0.03424	100.0	367.3	331.3	235.0	490.9	620.1	131.2	88.9	236.0	460.5	303.6	4.18	
Vampirovibrio	0.00033	0.00167	100.0	2076.4	1498.4	1547.3	1533.4	880.9	2394.3	2558.8	2079.1	2516.2	2476.5	2.42	
Variovorax	0.00056	0.00237	100.0	148.0	164.0	112.4	123.8	129.0	245.3	174.1	181.5	167.3	159.6	3.69	
Verrucomicrobia bacterium (uncultured)	0.00010	0.00067	100.0	346.6	268.8	348.6	268.8	188.2	177.1	155.9	118.6	117.8	134.3	2.63	
Verrucomicrobiales bacterium (uncultured)	0.00006	0.00042	100.0	273.6	296.0	282.4	311.5	249.6	712.2	444.9	521.3	590.6	391.4	2.54	
Virgibacillus	0.00003	0.00027	100.0	983.4	419.8	1679.3	812.4	175.7	83.6	69.9	86.5	129.0	74.8	3.39	
Vischeria sp CAUP Q 202	0.00840	0.01788	100.0	321.0	435.6	405.1	231.2	687.4	322.4	252.1	380.5	173.1	466.0	2.54	
Yonghaparkia	0.00000	0.00000	100.0	874.0	831.4	1119.8	739.0	484.3	295.1	185.5	204.1	156.8	286.0	3.61	
	**P-values**	**FDR**	**t0**	**CFD_t1**	**CRD_t1**	**MYC_t1**	**NFB_t1**	**NFB+MYC_t1**	**CFD_t2**	**CRD_t2**	**MYC_t2**	**NFB_t2**	**NFB+MYC_t2**	**LDA score**	**Notes**
Zavarzinella	0.00465	0.01133	100.0	139.4	124.2	123.0	109.5	77.7	128.6	157.1	148.5	146.6	138.7	3.06	

Table S2. Signature associated to the different time. Data grouped by time. Percentual standardization of values vs. t0. Negative differences greater than 10% are highlighted in red, while positive differences greater than 10% are highlighted in green.

	**P-values**	**FDR**	**t0**	**t1**	**t2**	**LDA score**	**Notes**
Abditibacterium	0.00013	0.00037	100.00	165.60	105.51	2.25	
Acidibacter	0.00000	0.00000	100.00	145.18	193.66	3.77	
Acidimicrobiia bacterium (uncultured)	0.00000	0.00000	100.00	59.88	46.59	4.05	
Acidobacteria bacterium (uncultured)	0.00357	0.00598	100.00	81.69	100.48	4.02	
Acidobacteriaceae bacterium (uncultured)	0.00015	0.00041	100.00	85.19	111.59	4.27	
Acidobacteriales bacterium (uncultured)	0.00001	0.00004	100.00	64.22	84.80	4.18	
Acidothermus	0.00000	0.00000	100.00	14.14	9.26	2.83	
Acidovorax	0.00000	0.00000	100.00	752.79	244.53	3.29	
Actinocorallia	0.00025	0.00061	100.00	70.91	49.17	2.88	
Actinomadura	0.00001	0.00004	100.00	84.29	64.68	2.71	
Actinomycetales bacterium (uncultured)	0.00000	0.00000	100.00	97.70	61.87	3.31	
Actinomycetospora	0.00721	0.01110	100.00	84.67	51.77	2.11	
Actinopolymorpha	0.01443	0.02104	100.00	51.37	37.41	2.35	
Actinotalea	0.00118	0.00232	100.00	91.86	62.39	2.33	
Adhaeribacter	0.00313	0.00541	100.00	145.06	113.23	3.52	
Aeromicrobium	0.00016	0.00044	100.00	223.75	228.12	4.3	
Aetherobacter	0.04286	0.05660	100.00	64.30	62.49	2.01	
Allocatelliglobosispora	0.01429	0.02096	100.00	90.52	68.90	1.99	
Allorhizobium, Neorhizobium, Pararhizobium, Rhizobium	0.00001	0.00004	100.00	201.50	215.30	4.33	
Altererythrobacter	0.00001	0.00006	100.00	232.87	197.07	3.39	
Amaricoccus	0.03415	0.04592	100.00	153.69	124.38	3.06	
Aminobacter	0.00005	0.00016	100.00	397.53	259.90	3.1	
Ammoniphilus	0.00597	0.00930	100.00	93.07	74.40	2.66	
Amycolatopsis	0.00000	0.00000	100.00	210.88	1440.07	3.37	
Anaerobacterium	0.00006	0.00018	100.00	36.91	26.78	2.72	
Anaerolinea	0.00001	0.00004	100.00	36.14	19.81	3.76	
Anaeromyxobacter	0.00006	0.00019	100.00	65.84	52.95	3.55	
Anaerosinus	0.02364	0.03271	100.00	41.63	31.15	2.48	
Aquabacterium	0.04542	0.05907	100.00	167.79	91.91	2.2	
Aquaspirillum arcticum group	0.00326	0.00558	100.00	314.85	241.18	1.96	
Aquicella	0.00015	0.00041	100.00	301.45	333.95	2.49	
Aquipuribacter	0.00002	0.00008	100.00	188.73	87.35	2.17	
Aquisphaera	0.00025	0.00061	100.00	55.28	46.19	2.75	
	**P-values**	**FDR**	**t0**	**t1**	**t2**	**LDA score**	**Notes**

Arenimonas	0.00001	0.00004	100.00	261.50	221.95	3.34	
Aridibacter	0.00073	0.00154	100.00	161.08	144.83	3.08	
Armatimonadetes bacterium (uncultured)	0.00006	0.00019	100.00	117.33	89.25	2.86	
Armatimonas	0.00007	0.00020	100.00	825.56	558.57	1.89	
Asticcacaulis	0.00000	0.00000	100.00	505.58	226.86	2.84	
Aurantisolimonas	0.00376	0.00619	100.00	142.49	160.35	2.66	
Azohydromonas	0.00000	0.00000	100.00	164.40	303.69	3.42	
Azospira	0.00000	0.00000	100.00	9232.93	1541.43	2.64	
Azospirillum	0.00005	0.00018	100.00	342.39	128.66	2.28	
Bacteroidetes bacterium (uncultured)	0.00013	0.00037	100.00	229.58	151.67	2.68	
Bauldia	0.00098	0.00197	100.00	174.58	156.71	2.88	
Bdellovibrio	0.00000	0.00000	100.00	293.77	181.25	2.99	
Blastocatella	0.00006	0.00018	100.00	158.23	139.09	3.35	
Blastococcus	0.00162	0.00307	100.00	126.98	101.36	3.78	
Blastopirellula	0.00000	0.00000	100.00	93.38	158.86	2.88	
Bosea	0.00004	0.00014	100.00	233.38	214.94	3.46	
Bradyrhizobium	0.00003	0.00011	100.00	136.84	161.35	3.83	
Brevibacillus	0.00015	0.00041	100.00	68.74	46.72	2.8	
Brevifollis	0.00000	0.00000	100.00	53.96	13.22	2.28	
Brevundimonas	0.00000	0.00000	100.00	130.22	56.29	2.55	
Bryobacter	0.04495	0.05876	100.00	100.32	116.34	3.54	
Burkholderia Caballeronia Paraburkholderia	0.00352	0.00592	100.00	66.39	50.03	3.29	
Burkholderiales bacterium Beta 02	0.00034	0.00078	100.00	396.16	345.66	2.05	
Caenimonas	0.00007	0.00021	100.00	188.43	186.31	3.89	
Caldicoprobacter	0.00003	0.00012	100.00	71.44	43.91	2.72	
Caldilineaceae bacterium (uncultured)	0.00161	0.00306	100.00	109.40	85.31	2.6	
Candidatus Accumulibacter	0.00479	0.00771	100.00	36.51	34.07	2.3	
Candidatus Chloroploca	0.00339	0.00575	100.00	198.95	207.06	2.66	
Candidatus Entotheonella	0.01782	0.02531	100.00	148.61	155.27	2.58	
Candidatus Koribacter	0.03791	0.05071	100.00	55.28	59.95	2.37	
Candidatus Nomurabacteria bacterium GW2011 GWE1 32 28	0.00259	0.00465	100.00	271.84	116.19	1.94	
Candidatus Solibacter	0.00486	0.00781	100.00	61.98	71.87	3.34	
Candidatus Udaeobacter	0.01443	0.02104	100.00	72.19	94.55	3.76	
Candidatus Xiphinematobacter	0.00007	0.00020	100.00	75.84	118.11	4.4	
Catelliglobosispora	0.00590	0.00926	100.00	103.21	142.43	1.57	
Caulobacter	0.00000	0.00000	100.00	219.74	66.89	3.23	
Cellulomonas	0.00000	0.00000	100.00	229.04	97.09	3.09	
Cellulosimicrobium	0.02053	0.02864	100.00	58.88	64.18	2.38	
	**P-values**	**FDR**	**t0**	**t1**	**t2**	**LDA score**	**Notes**
Cellvibrio	0.00000	0.00000	100.00	319.10	81.15	3.28	

Chelativorans	0.00114	0.00227	100.00	2374.45	598.33	3.17	
Chlamydomonas reinhardtii	0.00051	0.00111	100.00	933.30	550.15	2.01	
Chlorobi bacterium (uncultured)	0.00872	0.01331	100.00	38.14	37.88	3.04	
Chloroflexi bacterium (uncultured)	0.00000	0.00000	100.00	95.82	68.34	4.25	
Chloronema	0.00004	0.00012	100.00	499.18	516.91	2.85	
Chryseobacterium	0.02113	0.02933	100.00	56.08	130.26	2.05	
Chthoniobacter	0.00247	0.00447	100.00	142.27	132.44	3.99	
Chthonobacter	0.00008	0.00025	100.00	274.02	407.49	2.49	
Chthonomonas	0.00001	0.00006	100.00	145.52	121.07	2.84	
Clostridia bacterium (uncultured)	0.00377	0.00619	100.00	54.33	37.24	2.26	
Clostridium sensu stricto 1	0.00019	0.00049	100.00	61.83	42.53	3.18	
Clostridium sensu stricto 10	0.01485	0.02159	100.00	67.71	53.16	2.18	
Clostridium sensu stricto 12	0.02953	0.04023	100.00	92.58	65.52	2.39	
Clostridium sensu stricto 13	0.00338	0.00575	100.00	91.15	63.67	2.78	
Clostridium sensu stricto 8	0.00017	0.00046	100.00	71.94	52.11	2.9	
Cnuella	0.00004	0.00013	100.00	315.32	245.01	2.52	
Cohnella	0.00001	0.00005	100.00	79.50	52.17	3.47	
Comamonas	0.00000	0.00000	100.00	200.60	351.11	2.99	
Conexibacter	0.00000	0.00001	100.00	102.91	69.55	3.08	
Conexibacteraceae bacterium (uncultured)	0.00001	0.00004	100.00	63.74	42.42	2.41	
Coxiella	0.04539	0.05907	100.00	63.83	53.90	1.94	
Croceicoccus	0.00021	0.00053	100.00	543.21	453.29	2.39	
Cupriavidus	0.00000	0.00000	100.00	172.88	314.14	3.35	
Dactylosporangium	0.00033	0.00077	100.00	76.67	54.31	2.62	
Dechloromonas	0.01665	0.02381	100.00	42.79	34.85	2.36	
Deinococcus	0.00000	0.00000	100.00	3943.24	44207.72	3.2	
delta proteobacterium WX81	0.00083	0.00171	100.00	49.23	56.63	2.24	
Deltaproteobacteria bacterium GWC2 55 46	0.00002	0.00008	100.00	38.29	21.86	2.47	
Desmochloris halophila	0.00000	0.00001	100.00	3125.30	2037.64	3.18	
Desulfohalotomaculum	0.00431	0.00701	100.00	87.62	48.25	1.81	
Desulfosporosinus	0.00024	0.00058	100.00	50.59	31.53	2.32	
Desulfuromonadaceae bacterium (uncultured)	0.00292	0.00512	100.00	46.00	39.81	2.1	
Desulfuromonadales bacterium (uncultured)	0.00968	0.01473	100.00	69.10	79.73	3.02	
Devosia	0.00000	0.00000	100.00	239.97	197.15	4.11	
Dokdonella	0.00000	0.00001	100.00	504850.00	308630.00	2.4	
Domibacillus	0.00001	0.00003	100.00	80.17	192.70	3.21	
Dongia	0.00112	0.00224	100.00	67.36	78.72	3.53	

	**P-values**	**FDR**	**t0**	**t1**	**t2**	**LDA score**	**Notes**
Dyadobacter	0.00020	0.00050	100.00	200.43	370.11	3.6	

Ellin517	0.00035	0.00080	100.00	222.45	227.99	3.64	
Ellin6055	0.00000	0.00000	100.00	181.44	130.53	3.97	
Ellin6067	0.00069	0.00147	100.00	162.11	196.32	3.68	
Ensifer	0.00000	0.00000	100.00	145.32	214.69	3.79	
Enterobacter	0.00006	0.00019	100.00	154.37	1121.94	3.85	
Erythrobacter	0.00000	0.00002	100.00	4612.97	3801.18	2.7	
Ettlia pseudoalveolaris	0.00028	0.00067	100.00	782.65	1023.74	2.71	
Ferrovibrio	0.00086	0.00176	100.00	326.54	257.91	2.41	
Ferruginibacter	0.00021	0.00054	100.00	148.93	119.06	3.24	
Fervidicella	0.00262	0.00468	100.00	73.75	31.71	2.1	
Fictibacillus	0.00000	0.00000	100.00	63.27	48.77	3.68	
Fimbriiglobus	0.00000	0.00001	100.00	124.80	160.39	3.75	
Flavihumibacter	0.00012	0.00033	100.00	179.93	248.37	2.33	
Flavisolibacter	0.00008	0.00023	100.00	162.62	141.22	3.73	
Flavitalea	0.00000	0.00000	100.00	204.95	260.58	3.28	
Flavobacterium	0.00583	0.00916	100.00	288.11	225.75	3.15	
Frankineae bacterium (uncultured)	0.00117	0.00231	100.00	104.61	62.42	1.91	
Gaiella	0.00000	0.00000	100.00	74.88	46.95	4.96	
Galbitalea	0.00364	0.00604	100.00	108.92	73.94	3.03	
Gemmata	0.00019	0.00049	100.00	97.94	123.29	4.14	
Gemmatimonadales bacterium (uncultured)	0.00211	0.00383	100.00	61.96	75.66	3.21	
Gemmatimonadetes bacterium (uncultured)	0.00119	0.00233	100.00	100.32	130.54	3.14	
Gemmatimonas	0.00001	0.00004	100.00	188.96	180.10	3.81	
Gemmatirosa	0.00022	0.00055	100.00	168.40	91.58	1.84	
Geoalkalibacter	0.00006	0.00019	100.00	20.63	12.42	2.97	
Glycomyces	0.00165	0.00307	100.00	126.03	250.64	2.1	
Green Bay ferromanganous micronodule bacterium MND4 (uncultured)	0.00339	0.00575	100.00	52.88	47.96	1.99	
Halobacillus	0.00000	0.00000	100.00	14566.94	559.98	3.24	
Halocella	0.00596	0.00930	100.00	69.75	44.71	2.4	
Hassallia	0.00000	0.00000	100.00	666.62	141.63	3.42	
Herbidospora	0.01561	0.02250	100.00	132.82	98.46	2.38	
Herbinix	0.00003	0.00010	100.00	70.21	46.52	2.8	
Herpetosiphon	0.00138	0.00264	100.00	172.25	157.87	3.66	
Hirschia	0.00001	0.00006	100.00	420.09	476.96	3.32	
Hungateiclostridium	0.01329	0.01960	100.00	72.52	53.82	1.83	
Hyaloperonospora arabidopsidis	0.00000	0.00000	100.00	396.92	901.30	2.28	
Hydrogenophaga	0.01816	0.02569	100.00	263.04	201.51	2.11	
	**P-values**	**FDR**	**t0**	**t1**	**t2**	**LDA score**	**Notes**
Hymenobacter	0.03346	0.04523	100.00	170.18	102.19	1.93	
Hyphomicrobium	0.00508	0.00811	100.00	87.58	106.80	2.84	

Iamia	0.01229	0.01827	100.00	95.02	79.95	3.38	
Ideonella	0.00003	0.00010	100.00	478.93	496.36	2.77	
Ilumatobacter	0.00011	0.00030	100.00	79.95	63.91	3.31	
Inquilinus	0.02108	0.02933	100.00	239.59	248.03	2.28	
Intrasporangium	0.00163	0.00307	100.00	96.15	69.00	2.79	
Jahnella	0.02471	0.03410	100.00	108.72	544.18	2.48	
Kibdelosporangium	0.00614	0.00955	100.00	49.50	52.95	1.95	
Knoellia	0.00002	0.00007	100.00	96.54	76.33	4.09	
Kribbella	0.00039	0.00087	100.00	74.30	75.02	2.84	
Lachnoclostridium	0.02677	0.03666	100.00	78.68	59.80	2.14	
Lacibacter	0.00566	0.00896	100.00	186.86	201.28	3.07	
Lacunisphaera	0.00000	0.00001	100.00	253.24	112.76	2.99	
Lapillicoccus	0.02032	0.02843	100.00	127.91	92.43	2.1	
Lautropia	0.00341	0.00576	100.00	193.04	200.68	2.37	
Lechevalieria	0.00000	0.00000	100.00	141.13	230.82	3.65	
Legionella	0.00000	0.00000	100.00	289.76	206.63	2.96	
Leptolyngbya EcFYyyy 00	0.00000	0.00000	100.00	7500.50	4033.34	4.45	
Litorilinea	0.01886	0.02660	100.00	139.76	127.06	3.27	
Longimicrobium	0.00028	0.00066	100.00	343.34	434.46	2.82	
Luedemannella	0.00000	0.00001	100.00	57.56	41.22	3.44	
Luteimonas	0.01347	0.01980	100.00	221.91	243.29	2.55	
Luteitalea	0.03366	0.04538	100.00	128.21	135.69	3.07	
Luteolibacter	0.00004	0.00013	100.00	219.70	399.73	4.04	
Lysinimonas	0.00123	0.00237	100.00	164.08	116.39	2.53	
Lysobacter	0.00098	0.00197	100.00	141.02	128.59	3.72	
Marisediminicola	0.00373	0.00616	100.00	140.63	74.51	1.8	
Marmoricola	0.00005	0.00016	100.00	80.46	73.53	3.97	
Meiothermus	0.00015	0.00040	100.00	20751.27	12138.70	2.41	
Mesorhizobium	0.00000	0.00001	100.00	236.76	225.31	4	
Methylobacillus	0.00000	0.00000	100.00	1344.30	5583.53	3.12	
Methyloceanibacter	0.01650	0.02365	100.00	60.65	66.49	2.09	
Methylocella	0.00000	0.00001	100.00	297.57	120.70	2.22	
Methylorosula	0.00010	0.00029	100.00	149.44	100.47	2.34	
Methylotenera	0.00000	0.00000	100.00	323.56	897.23	3.58	
Microbacterium	0.00050	0.00110	100.00	146.58	227.50	3.64	
Micropepsis	0.00000	0.00000	100.00	14337.25	8230.56	2.66	
Microterricola	0.01785	0.02531	100.00	90.44	72.49	2.03	
	**P-values**	**FDR**	**t0**	**t1**	**t2**	**LDA score**	**Notes**
Microvirga	0.00446	0.00723	100.00	129.29	144.28	4.38	
Mitsuaria	0.00000	0.00000	100.00	2527.43	10034.29	3.82	
Mobilitalea	0.03109	0.04214	100.00	57.44	47.91	2.08	

Mucilaginibacter	0.00000	0.00000	100.00	81.34	29.73	2.68	
Mycobacterium	0.00000	0.00000	100.00	88.52	74.89	3.69	
Myxococcus	0.01221	0.01820	100.00	71.95	46.98	2.85	
Nannocystis	0.01516	0.02198	100.00	189.35	194.98	2.29	
Niastella	0.00000	0.00000	100.00	183.07	237.36	3.19	
Nibribacter	0.00198	0.00364	100.00	137.57	85.57	2.33	
Nitratireductor	0.00249	0.00450	100.00	187.46	173.88	2.25	
Nitrolancea	0.01245	0.01846	100.00	99.14	69.70	2.34	
Nitrosomonadaceae bacterium (uncultured)	0.04402	0.05792	100.00	85.89	59.28	1.7	
Nitrosomonas	0.00000	0.00001	100.00	578.57	397.54	2.84	
Nitrosospira	0.00115	0.00228	100.00	230.42	129.48	2.9	
Nitrospira	0.04041	0.05391	100.00	116.31	123.78	3.88	
Nocardia	0.01903	0.02676	100.00	131.73	125.36	2.57	
Nocardioides	0.00000	0.00000	100.00	88.74	66.28	4.23	
Nodosilinea PCC 7104	0.00000	0.00001	100.00	5834.12	6352.75	3.61	
Nonomuraea	0.00058	0.00127	100.00	84.53	60.18	2.9	
Nordella	0.00003	0.00010	100.00	108.28	139.13	3.69	
Nostoc PCC 7524	0.00047	0.00104	100.00	191.66	94.46	2.23	
Noviherbaspirillum	0.01179	0.01768	100.00	154.38	159.00	3.75	
Novosphingobium	0.00001	0.00006	100.00	91.43	52.89	2.89	
Ohtaekwangia	0.00024	0.00059	100.00	204.79	184.18	3.53	
Oligoflexus	0.02479	0.03412	100.00	147.76	107.93	2.78	
Oscillatoria SAG 1459 8	0.00004	0.00013	100.00	3455.45	4146.83	2.47	
Oscillochloris	0.00015	0.00042	100.00	325.79	339.23	2.2	
Paenarthrobacter	0.00204	0.00373	100.00	96.30	165.86	3.44	
Paenisporosarcina	0.00093	0.00190	100.00	82.60	61.13	3.34	
Pajaroellobacter	0.04823	0.06226	100.00	95.93	82.97	2.8	
Panacagrimonas	0.00008	0.00023	100.00	455.95	320.89	2.65	
Parasegetibacter	0.00001	0.00003	100.00	248.18	198.88	3.12	
Parviterribacter	0.00001	0.00005	100.00	97.54	65.57	2.96	
Paucibacter	0.00146	0.00278	100.00	237.62	223.34	2.59	
Pedinomonas minor	0.00206	0.00376	100.00	203.71	96.66	2.3	
Pedobacter	0.00000	0.00000	100.00	158.64	101.15	3.4	
Pedococcus Phycicoccus	0.00007	0.00021	100.00	80.77	62.43	2.79	
Pedomicrobium	0.00001	0.00003	100.00	71.47	94.50	3.61	
Pelomonas	0.00002	0.00008	100.00	499.64	266.69	2.68	

	P-values	FDR	t0	t1	t2	LDA score	Notes
Pelosinus	0.00001	0.00005	100.00	53.46	39.56	2.85	
Peredibacter	0.00000	0.00002	100.00	197.30	153.48	2.96	
Phaselicystis	0.00164	0.00307	100.00	175.22	136.49	2.71	
Phenylobacterium	0.00000	0.00000	100.00	104.02	64.79	2.87	

	P-values	FDR	t0	t1	t2	LDA score	Notes
Phyllobacterium	0.00360	0.00601	100.00	133.78	161.27	3.15	
Pirellula	0.00036	0.00082	100.00	105.19	134.86	4.26	
Piscinibacter	0.00000	0.00001	100.00	186.67	281.18	2.63	
Planctomicrobium	0.00018	0.00047	100.00	133.04	205.55	2.66	
Planctomycetaceae bacterium (uncultured)	0.00022	0.00055	100.00	135.66	57.30	1.87	
Planctomycetales bacterium (uncultured)	0.00000	0.00002	100.00	146.42	185.56	2.96	
planctomycete (uncultured)	0.00000	0.00001	100.00	166.37	197.99	4.18	
Planctomycete WY108	0.00056	0.00122	100.00	309.44	335.69	2.01	
Planctopirus	0.00000	0.00000	100.00	454.31	315.89	3.27	
Planifilum	0.00132	0.00253	100.00	59.81	30.38	2.93	
Planoglabratella opercularis	0.00003	0.00010	100.00	620.20	413.58	2.43	
Polaromonas	0.03102	0.04214	100.00	134.28	119.49	2.48	
Polyangiaceae bacterium (uncultured)	0.00023	0.00056	100.00	231.40	471.54	2.08	
Polyangium brachysporum group	0.00000	0.00000	100.00	263.08	595.97	3.49	
Polycyclovorans	0.00032	0.00074	100.00	163.39	67.11	3.19	
Pontibacter	0.00002	0.00007	100.00	205.48	136.82	3.54	
Promicromonospora	0.01558	0.02250	100.00	57.50	68.90	3.02	
Prosthecomicrobium	0.00077	0.00161	100.00	231.56	260.12	2.3	
Proteiniborus	0.00452	0.00731	100.00	74.77	53.17	2.2	
Pseudenhygromyxa	0.00065	0.00140	100.00	329.77	136.47	2.21	
Pseudoduganella	0.00002	0.00006	100.00	630.91	652.93	3.22	
Pseudolabrys	0.00003	0.00010	100.00	159.94	88.27	3.6	
Pseudomuriella schumacherensis	0.00000	0.00001	100.00	1087.39	1169.81	3.27	
Pseudonocardia	0.00306	0.00531	100.00	100.33	80.59	2.71	
Pseudorhodoplanes	0.00123	0.00237	100.00	145.55	153.49	2.82	
Pseudoxanthomonas	0.00028	0.00067	100.00	222.82	307.31	3.71	
Qipengyuania	0.00000	0.00000	100.00	272.28	142.79	3.6	
Ramlibacter	0.00001	0.00003	100.00	189.70	218.90	3.92	
Reyranella	0.00025	0.00060	100.00	141.88	159.08	3.38	
Rhizobacter	0.00000	0.00003	100.00	185.91	195.67	3.79	
Rhizocola	0.04902	0.06282	100.00	71.86	57.09	1.96	
Rhizorhapis	0.00683	0.01058	100.00	214.39	591.18	2.59	
Rhodanobacter	0.00000	0.00000	100.00	17194.97	3395.27	3.29	
Rhodobacter	0.00000	0.00001	100.00	347.98	165.53	3.27	
Rhodococcus	0.00080	0.00167	100.00	52.60	59.52	2.78	
Rhodocytophaga	0.00027	0.00064	100.00	207.03	207.73	3.05	
Rhodoferax	0.00000	0.00000	100.00	276.46	101.13	2.67	
Rhodopirellula	0.00011	0.00030	100.00	185.48	179.04	3.38	
Rhodoplanes	0.00091	0.00186	100.00	105.80	134.39	3.33	
Rhodopseudomonas	0.00000	0.00000	100.00	1377.41	714.93	2.71	

Risungbinella	0.02686	0.03668	100.00	91.01	83.40	2.14	
Roseisolibacter	0.00014	0.00040	100.00	160.31	113.42	2.42	
Roseomonas	0.00105	0.00210	100.00	178.02	157.48	2.88	
Rubellimicrobium	0.00004	0.00015	100.00	226.86	177.40	3.8	
Rubrobacter	0.00303	0.00529	100.00	109.22	86.59	4.16	
Rubrobacterales bacterium (uncultured)	0.00000	0.00000	100.00	68.94	42.53	4.33	
Rubrobacteria bacterium (uncultured)	0.00000	0.00000	100.00	80.48	51.20	3.82	
Ruminiclostridium	0.00004	0.00014	100.00	54.66	38.85	3.07	
Saccharothrix	0.00034	0.00079	100.00	149.08	92.58	2.73	
Salinispora	0.00003	0.00012	100.00	65.22	48.29	2.46	
Sandaracinus	0.00023	0.00056	100.00	119.59	84.82	2.59	
Schlesneria	0.00000	0.00000	100.00	4311.53	1130.52	2.33	
Segetibacter	0.00002	0.00009	100.00	109.75	63.36	2.57	
Shimazuella	0.00121	0.00235	100.00	84.78	61.53	2.77	
Shinella	0.00000	0.00002	100.00	571.21	512.36	3.39	
Sideroxydans	0.00000	0.00002	100.00	8.26	4.83	3.2	
Simplicispira	0.00000	0.00000	100.00	2655.33	398.23	2.63	
Smaragdicoccus	0.00364	0.00604	100.00	138.15	271.17	2.57	
Solibacillus	0.00527	0.00839	100.00	67.53	55.30	2.1	
Solimonas	0.00000	0.00000	100.00	378.62	97.88	2.41	
Solirubrobacter	0.00000	0.00000	100.00	83.40	59.42	3.95	
Sorangium	0.00064	0.00138	100.00	71.70	85.08	2.66	
Sphingoaurantiacus	0.00002	0.00009	100.00	161.62	117.48	2.77	
Sphingobium	0.00000	0.00000	100.00	1407.74	2049.47	4.39	
Sphingomonas	0.00000	0.00000	100.00	120.23	92.25	4.33	
Sphingopyxis	0.00000	0.00000	100.00	450.38	204.00	3.44	
Spirochaeta	0.00001	0.00004	100.00	14.27	9.37	2.9	
Sporacetigenium	0.00268	0.00476	100.00	104.86	65.45	2.67	
Sporichthya	0.00400	0.00652	100.00	246.52	232.02	2.3	
Sporosarcina	0.00003	0.00010	100.00	84.17	58.50	2.91	
Stenotrophobacter	0.04622	0.05983	100.00	123.96	100.60	2.55	
Steroidobacter	0.00001	0.00004	100.00	87.04	120.41	3.59	
Streptomyces	0.00021	0.00053	100.00	121.38	140.17	3.93	
Streptosporangium	0.00738	0.01129	100.00	104.43	64.11	2.14	

	P-values	FDR	t0	t1	t2	LDA score	Notes
Sumerlaea	0.04075	0.05423	100.00	132.17	132.65	2.91	
Symbiobacterium	0.00007	0.00020	100.00	66.02	39.35	2.71	
Synechococcus IR11	0.00000	0.00002	100.00	6951.69	7731.46	3.23	
Syntrophobacter	0.00017	0.00045	100.00	35.49	26.04	2.76	
Syntrophobacterales bacterium (uncultured)	0.04161	0.05509	100.00	86.95	111.80	2.53	
Tahibacter	0.00261	0.00468	100.00	368.95	319.61	2.82	

Telmatocola	0.01928	0.02705	100.00	147.86	109.04	2.03
Tepidisphaera	0.00737	0.01129	100.00	189.81	162.71	3.05
Terrimicrobium	0.00001	0.00004	100.00	255.50	335.78	2.66
Terrimonas	0.00000	0.00001	100.00	159.54	212.41	3.73
Tetradesmus obliquus	0.00035	0.00080	100.00	506.88	494.17	2.19
Thermincola	0.04555	0.05910	100.00	52.92	42.58	2.2
Thermoactinomyces	0.00286	0.00507	100.00	52.86	34.93	2.67
Thermoflavimicrobium	0.00500	0.00801	100.00	70.33	46.06	2.58
Thermomicrobia bacterium (uncultured)	0.00146	0.00278	100.00	99.24	74.24	2.72
Thermopolyspora	0.01018	0.01540	100.00	63.57	38.05	2
Truepera	0.00000	0.00000	100.00	216.96	830.59	3.41
Tumebacillus	0.00000	0.00002	100.00	70.60	36.28	3.7
Tychonema CCAP 1459 11B	0.00019	0.00049	100.00	413.40	244.05	3.96
Vampirovibrio	0.00000	0.00000	100.00	1506.29	2404.98	2.39
Variovorax	0.00001	0.00003	100.00	136.00	185.53	3.46
Verrucomicrobia bacterium (uncultured)	0.00000	0.00001	100.00	282.54	140.74	2.5
Verrucomicrobiales bacterium (uncultured)	0.00000	0.00000	100.00	282.63	532.08	2.39
Virgibacillus	0.00000	0.00000	100.00	791.94	88.75	3.03
Vischeria sp CAUP Q 202	0.01043	0.01573	100.00	416.33	318.81	2.27
Vulgatibacter	0.01062	0.01597	100.00	99.54	78.66	2.08
Yonghaparkia	0.00000	0.00000	100.00	801.75	225.49	3.45
Zavarzinella	0.00039	0.00087	100.00	114.55	143.90	2.8

Table S3. Signature associated to each treatment (Label). Data grouped by label. Percentual standardization of values vs. CFD. Negative differences greater than 10% are highlighted in red, while positive differences greater than 10% are highlighted in green.

	P-values	FDR	t0	CFD_t1	CRD_t1	MYC_t1	NFB_t1	NFB+MYC_t1	CFD_t2	CRD_t2	MYC_t2	NFB_t2	NFB+MYC_t2	LDA score
Abditibacterium	0.00126	0.00440	55.93	100.00	69.93	117.91	93.72	84.69	100.00	78.34	77.21	50.20	84.44	2.58
Acidibacter	0.00018	0.00101	73.00	100.00	105.70	100.15	114.01	109.28	100.00	130.17	93.06	121.39	97.55	3.92
Acidimicrobiia bacterium (uncultured)	0.00000	0.00002	176.86	100.00	119.52	95.47	105.49	107.74	100.00	84.81	90.55	89.10	91.21	4.07
Acidobacteria bacterium (uncultured)	0.01339	0.02642	104.40	100.00	85.10	78.10	85.19	77.13	100.00	108.37	120.60	124.52	98.33	4.35
Acidobacteriaceae bacterium (uncultured)	0.00227	0.00674	104.66	100.00	88.07	83.49	91.02	82.52	100.00	113.10	118.73	123.41	97.20	4.5
Acidobacteriales bacterium (uncultured)	0.00013	0.00081	142.47	100.00	92.88	84.19	91.96	87.51	100.00	121.18	132.99	149.88	98.30	4.3
Acidothermus	0.00004	0.00032	778.76	100.00	129.37	69.44	124.76	122.03	100.00	60.19	64.63	62.35	81.33	2.84
Acidovorax	0.00000	0.00002	14.42	100.00	141.50	147.50	114.94	43.76	100.00	44.33	69.85	49.69	77.52	3.44
Actinobacterium (uncultured)	0.00002	0.00020	126.94	100.00	117.87	101.47	96.74	78.89	100.00	96.05	91.83	94.26	77.53	4.16
Actinocorallia	0.00038	0.00182	161.49	100.00	147.63	132.61	125.01	69.57	100.00	141.22	87.46	162.47	63.83	3.03
Actinomadura	0.00081	0.00303	128.94	100.00	97.42	109.25	105.38	131.44	100.00	104.78	94.70	115.42	85.68	2.83
Actinomycetales bacterium (uncultured)	0.00000	0.00000	104.69	100.00	113.05	99.53	100.80	97.73	100.00	78.85	83.02	94.77	90.20	3.45
Actinomycetospora	0.01895	0.03391	164.63	100.00	136.74	69.27	157.14	225.00	100.00	94.44	91.20	142.97	82.94	2.4
Actinopolymorpha	0.03957	0.06041	228.30	100.00	117.58	99.20	101.10	166.24	100.00	51.46	95.98	89.10	75.98	2.44
Actinotalea	0.03516	0.05463	110.46	100.00	96.59	101.40	85.32	124.01	100.00	79.86	89.91	79.32	119.38	2.53
Adhaeribacter	0.00203	0.00625	81.69	100.00	88.88	138.35	127.66	140.08	100.00	98.92	103.80	93.82	133.24	3.72
Aeromicrobium	0.00395	0.01016	46.61	100.00	91.66	123.35	107.71	101.16	100.00	84.06	62.62	50.70	58.85	4.53
Allorhizobium, Neorhizobium, Pararhizobium, Rhizobium	0.00127	0.00440	59.34	100.00	108.46	107.09	139.16	141.60	100.00	88.29	85.64	71.39	76.79	4.46
Alsobacter	0.01812	0.03278	96.80	100.00	102.28	162.17	151.84	162.54	100.00	121.32	114.96	107.13	144.77	2.87
Altererythrobacter	0.00068	0.00265	48.25	100.00	158.04	107.69	111.41	84.05	100.00	93.17	69.12	71.47	89.37	3.63
	**P-values**	**FDR**	**t0**	**CFD_t1**	**CRD_t1**	**MYC_t1**	**NFB_t1**	**NFB+MYC_t1**	**CFD_t2**	**CRD_t2**	**MYC_t2**	**NFB_t2**	**NFB+MYC_t2**	**LDA score**
Amaricoccus	0.00886	0.01862	90.95	100.00	115.85	131.23	144.04	206.74	100.00	90.67	92.01	100.28	116.15	3.44
Aminobacter	0.00000	0.00001	49.99	100.00	75.86	394.00	120.99	327.24	100.00	148.78	165.41	131.66	151.39	3.47

Ammoniphilus	0.00219	0.00665	139.93	100.00	138.35	115.38	108.09	187.50	100.00	111.22	76.27	82.41	91.82	3.11
Amycolatopsis	0.00000	0.00000	27.19	100.00	69.04	71.10	29.16	19.12	100.00	30.35	43.20	50.34	62.68	3.63
Anaerobacterium	0.01310	0.02594	292.00	100.00	103.93	126.57	87.67	123.08	100.00	132.75	121.23	125.86	137.06	2.75
Anaerolinea	0.00142	0.00468	363.59	100.00	89.33	117.15	113.81	234.88	100.00	73.87	106.44	117.11	98.14	3.78
Anaeromyxobacter	0.00344	0.00929	169.92	100.00	103.97	91.81	114.94	146.16	100.00	99.56	120.64	110.37	105.27	3.58
Anaeromyxobacter dehalogenans	0.03063	0.04861	97.30	100.00	124.76	252.08	180.05	307.10	100.00	221.86	172.02	103.63	140.09	2.08
Aquaspirillum arcticum group	0.02385	0.04018	55.82	100.00	112.68	219.43	244.48	207.59	100.00	96.18	63.48	74.55	79.96	2.15
Aquicella	0.01029	0.02095	21.85	100.00	67.19	58.74	58.45	43.99	100.00	110.20	94.65	111.06	71.33	2.67
Aquipuribacter	0.00986	0.02031	48.55	100.00	64.99	100.55	91.44	102.26	100.00	120.81	110.51	103.04	113.81	2.28
Aquisphaera	0.02632	0.04322	197.76	100.00	120.35	93.28	99.44	131.61	100.00	82.16	122.29	112.14	79.81	2.82
Arenimonas	0.00034	0.00170	37.27	100.00	115.24	95.69	89.80	86.41	100.00	75.32	73.62	42.03	81.60	3.45
Aridibacter	0.00691	0.01510	58.11	100.00	76.69	96.44	102.33	92.89	100.00	97.79	83.23	94.62	114.31	3.18
Armatimonadetes bacterium (uncultured)	0.00480	0.01163	93.19	100.00	98.61	126.28	111.14	112.76	100.00	119.07	116.07	118.76	110.22	3.16
Armatimonas	0.00197	0.00614	16.43	100.00	177.13	158.49	143.40	101.85	100.00	51.22	99.18	52.77	113.27	2.02
Arthrobacter	0.00735	0.01597	91.09	100.00	81.03	118.89	108.86	117.21	100.00	85.89	104.76	85.61	154.02	5.42
Asanoa	0.02186	0.03769	208.59	100.00	249.46	131.40	143.17	252.70	100.00	73.24	128.12	110.22	90.98	2.25
Asticcacaulis	0.00003	0.00027	25.47	100.00	257.03	95.93	105.66	81.04	100.00	203.88	148.19	78.10	56.28	3.19
Azohydromonas	0.00000	0.00000	64.21	100.00	93.38	113.44	103.52	118.50	100.00	100.43	85.07	84.65	99.96	3.46
Azospira	0.00000	0.00004	1.62	100.00	216.85	187.39	173.33	77.16	100.00	83.74	29.23	10.95	8.65	2.8
Azospirillum	0.00428	0.01084	48.87	100.00	122.29	89.68	308.18	206.79	100.00	208.76	97.78	152.34	118.97	2.63
Bacteroidetes bacterium (uncultured)	0.00342	0.00929	42.66	100.00	89.66	102.19	109.00	89.40	100.00	92.84	71.10	64.36	62.84	2.75
Bauldia	0.01958	0.03465	66.33	100.00	146.05	90.10	140.86	98.72	100.00	178.98	96.91	92.07	70.84	3.22
Bdellovibrio	0.00000	0.00001	37.43	100.00	108.90	114.00	112.28	115.04	100.00	123.64	87.92	88.21	127.48	3.02
	**P-values**	**FDR**	**t0**	**CFD_t1**	**CRD_t1**	**MYC_t1**	**NFB_t1**	**NFB+MYC_t1**	**CFD_t2**	**CRD_t2**	**MYC_t2**	**NFB_t2**	**NFB+MYC_t2**	**LDA score**
Blastocatella	0.00278	0.00802	60.65	100.00	85.13	109.74	102.06	84.61	100.00	93.02	96.46	106.09	104.65	3.49
Blastococcus	0.00575	0.01326	92.14	100.00	96.80	126.02	122.42	140.89	100.00	109.09	95.49	102.07	119.94	4.14
Blastopirellula	0.00005	0.00041	97.10	100.00	92.48	93.07	89.53	78.53	100.00	116.50	132.67	146.59	98.92	3.12

Bosea	0.00137	0.00465	54.34	100.00	104.45	125.82	136.00	167.63	100.00	107.31	94.71	73.22	91.67	3.65
Bradyrhizobium	0.00245	0.00714	77.15	100.00	118.46	91.57	113.45	102.65	100.00	105.72	106.07	116.06	95.90	3.94
Brevibacillus	0.02128	0.03693	186.83	100.00	138.11	119.09	116.55	167.25	100.00	136.79	96.89	95.92	89.26	2.85
Brevifollis	0.00001	0.00008	197.65	100.00	128.52	87.06	56.58	158.61	100.00	167.29	129.64	83.05	96.73	2.3
Brevundimonas	0.00001	0.00016	86.33	100.00	125.43	136.99	97.85	104.91	100.00	61.79	42.09	36.84	92.64	2.78
Burkholderia Caballeronia Paraburkholderia	0.02886	0.04623	193.33	100.00	143.06	113.10	108.97	174.69	100.00	96.56	46.62	42.48	54.63	3.43
Burkholderiales bacterium Beta 02	0.00162	0.00509	16.65	100.00	62.31	57.83	59.44	49.21	100.00	77.25	58.10	49.01	61.90	2.27
Caenimonas	0.00039	0.00184	60.89	100.00	89.15	127.32	124.90	133.84	100.00	94.26	75.82	71.63	101.61	4.02
Caldicoprobacter	0.00498	0.01197	152.70	100.00	100.13	118.59	83.08	144.85	100.00	111.36	94.20	100.78	113.02	2.75
Caldilineaceae bacterium (uncultured)	0.03295	0.05183	98.24	100.00	128.36	93.72	100.14	113.39	100.00	95.48	93.97	103.81	89.87	2.92
Candidatus Alysiosphaera	0.02903	0.04636	101.52	100.00	100.56	118.61	129.33	167.81	100.00	83.79	96.72	107.90	99.86	3.87
Candidatus Chloroploca	0.02749	0.04487	71.83	100.00	133.06	123.67	162.94	192.44	100.00	58.59	109.60	92.29	89.04	2.86
Candidatus Nomurabacteria bacterium GW2011 GWE1 32 28	0.02283	0.03888	36.33	100.00	76.90	110.97	97.03	110.44	100.00	182.25	60.33	60.55	55.77	2.08
Candidatus Udaeobacter	0.00406	0.01038	102.64	100.00	93.71	61.61	69.33	44.27	100.00	115.31	116.03	138.27	89.17	4.18
Candidatus Xiphinematobacter	0.00222	0.00669	110.40	100.00	79.44	79.51	87.01	72.14	100.00	118.69	133.01	153.43	98.23	4.71
Caulobacter	0.00000	0.00001	61.69	100.00	270.89	109.04	108.88	85.60	100.00	93.55	71.05	55.47	75.23	3.63
Cellulomonas	0.00006	0.00045	81.06	100.00	268.98	141.20	236.14	176.42	100.00	113.53	112.53	81.87	116.28	3.38
Cellvibrio	0.00008	0.00055	39.65	100.00	233.39	107.29	86.33	103.20	100.00	89.13	69.22	48.56	83.65	3.64
Chelativorans	0.00000	0.00000	85.71	100.00	164.31	5007.14	223.10	5052.89	100.00	83.46	871.34	74.97	747.46	3.57
Chlamydomonas reinhardtii	0.01368	0.02679	12.30	100.00	85.49	143.26	50.44	198.35	100.00	200.10	101.83	107.87	204.92	2.27
Chloroflexi bacterium (uncultured)	0.00000	0.00000	115.99	100.00	107.66	112.55	108.84	126.81	100.00	89.33	92.32	94.47	95.61	4.4
	**P-values**	**FDR**	**t0**	**CFD_t1**	**CRD_t1**	**MYC_t1**	**NFB_t1**	**NFB+MYC_t1**	**CFD_t2**	**CRD_t2**	**MYC_t2**	**NFB_t2**	**NFB+MYC_t2**	**LDA score**
Chloronema	0.00068	0.00265	40.51	100.00	218.51	258.93	234.27	206.48	100.00	118.18	243.24	189.80	177.62	3.05
Chryseobacterium	0.02952	0.04699	139.71	100.00	188.87	34.11	37.11	26.17	100.00	54.32	171.92	100.67	185.00	2.42
Chthoniobacter	0.03489	0.05439	68.12	100.00	82.64	104.96	103.54	94.39	100.00	110.60	104.97	98.87	100.86	4.1
Chthonobacter	0.00444	0.01097	51.89	100.00	111.52	182.94	179.96	141.67	100.00	129.22	77.49	58.03	86.50	2.68

	P-values	FDR	t0	CFD_t1	CRD_t1	MYC_t1	NFB_t1	NFB+MYC_t1	CFD_t2	CRD_t2	MYC_t2	NFB_t2	NFB+MYC_t2	LDA score
Chthonomonas	0.00051	0.00222	75.59	100.00	110.53	113.90	106.55	119.49	100.00	110.33	104.39	120.51	130.22	2.95
Clostridia bacterium (uncultured)	0.04274	0.06468	235.80	100.00	128.46	138.13	118.77	156.48	100.00	52.28	61.09	108.60	104.96	2.35
Clostridium sensu stricto 1	0.00618	0.01394	162.72	100.00	93.61	76.84	89.13	140.48	100.00	66.74	121.28	113.39	94.33	3.27
Clostridium sensu stricto 12	0.01778	0.03250	130.07	100.00	104.40	91.85	112.43	189.79	100.00	137.35	136.69	153.91	114.52	2.83
Clostridium sensu stricto 13	0.01969	0.03465	107.81	100.00	94.50	76.41	83.72	133.95	100.00	114.81	105.70	135.20	117.30	3.06
Clostridium sensu stricto 8	0.00443	0.01097	155.08	100.00	103.54	100.00	96.46	156.43	100.00	101.45	109.94	118.54	107.72	2.94
Cnuella	0.00159	0.00502	32.16	100.00	85.52	125.30	80.81	118.46	100.00	121.64	83.80	97.31	124.33	2.65
Cohnella	0.00057	0.00237	164.50	100.00	125.66	120.91	124.45	181.64	100.00	117.12	119.09	126.38	118.81	3.61
Comamonas	0.00001	0.00011	50.44	100.00	98.11	101.20	103.27	103.41	100.00	86.34	78.12	75.12	79.04	3.1
Conexibacter	0.00036	0.00177	105.36	100.00	114.41	114.87	107.53	106.14	100.00	71.66	71.47	75.74	89.01	3.23
Conexibacteraceae bacterium (uncultured)	0.00043	0.00196	157.92	100.00	112.03	114.90	113.45	64.72	100.00	58.48	75.96	100.89	79.18	2.49
Croceicoccus	0.02866	0.04605	26.54	100.00	179.08	165.47	149.67	129.17	100.00	114.10	84.28	76.17	104.95	2.5
Cupriavidus	0.00001	0.00011	70.50	100.00	106.43	81.65	103.43	212.84	100.00	200.21	115.14	115.04	128.68	3.59
Dactylosporangium	0.00047	0.00210	165.36	100.00	127.38	100.77	108.46	194.11	100.00	71.85	79.31	107.93	79.63	2.82
Deinococcus	0.00000	0.00001	2.13	100.00	68.07	88.95	50.74	113.66	100.00	61.95	26.98	14.75	30.42	3.53
delta proteobacterium WX81	0.03074	0.04864	165.96	100.00	68.80	80.36	70.83	88.38	100.00	100.71	114.23	124.43	78.37	2.3
Deltaproteobacteria bacterium GWC2 55 46	0.00375	0.00988	349.11	100.00	89.82	113.75	113.54	248.71	100.00	65.02	78.21	103.76	77.75	2.5
Desmochloris halophila	0.00047	0.00210	2.11	100.00	53.80	61.15	50.98	63.08	100.00	102.74	89.64	105.08	104.35	3.36
Desulfohalotomaculum	0.01215	0.02424	173.74	100.00	131.61	112.34	158.56	253.67	100.00	110.58	96.03	142.70	95.49	2.11
Desulfosporosinus	0.00586	0.01341	263.23	100.00	119.85	112.18	134.25	196.94	100.00	135.93	115.23	104.03	123.68	2.35
Devosia	0.00000	0.00007	45.20	100.00	135.96	99.88	112.95	92.44	100.00	103.21	71.58	64.34	64.87	4.27
Dokdonella	0.00003	0.00029	0.03	100.00	255.20	135.04	285.73	52.48	100.00	274.91	94.84	99.39	149.48	2.64
Domibacillus	0.00066	0.00260	108.56	100.00	82.00	79.34	80.06	92.79	100.00	65.19	88.82	76.71	142.56	3.49
Dongia	0.00993	0.02038	126.56	100.00	91.88	73.72	81.21	77.98	100.00	126.12	113.42	130.60	104.56	3.64
Dyadobacter	0.00011	0.00069	46.70	100.00	126.31	110.56	83.85	49.37	100.00	37.31	113.58	31.65	48.01	3.9
Ellin517	0.00066	0.00261	31.91	100.00	71.88	69.64	67.12	46.06	100.00	122.00	97.57	98.84	95.73	3.87

Ellin6055	0.00009	0.00063	51.04	100.00	85.24	100.76	94.74	83.35	100.00	89.45	76.61	74.77	102.58	4.05
Ellin6067	0.01801	0.03278	62.41	100.00	94.22	110.62	116.87	85.37	100.00	110.88	81.58	65.16	82.53	3.86
Ensifer	0.00000	0.00002	65.00	100.00	92.42	95.42	89.45	95.10	100.00	104.14	115.23	107.01	104.34	3.85
Enterobacter	0.00443	0.01097	19.86	100.00	12.98	13.53	10.53	14.12	100.00	17.27	19.47	17.63	91.80	4.18
Erythrobacter	0.00076	0.00286	3.60	100.00	188.10	408.86	97.90	66.86	100.00	65.21	48.95	66.31	122.60	3.1
Ettlia pseudoalveolaris	0.01813	0.03278	16.75	100.00	85.17	155.79	121.69	196.01	100.00	67.66	53.96	42.36	415.12	3.23
Ferrovibrio	0.02576	0.04266	30.56	100.00	87.00	103.70	110.62	98.19	100.00	104.83	59.56	92.00	68.03	2.47
Ferruginibacter	0.00557	0.01292	70.05	100.00	84.27	112.08	115.58	110.62	100.00	100.06	101.38	96.07	110.77	3.37
Fervidicella	0.00681	0.01505	144.43	100.00	60.85	93.84	43.49	232.84	100.00	281.19	194.44	401.73	313.11	2.44
Fictibacillus	0.00004	0.00035	176.42	100.00	107.33	106.42	102.78	140.96	100.00	193.83	100.00	126.02	135.64	3.77
Fimbriiglobus	0.00031	0.00162	74.42	100.00	96.11	89.11	96.99	81.73	100.00	110.83	118.25	121.45	100.45	3.86
Flavihumibacter	0.00853	0.01806	64.77	100.00	87.81	132.08	131.01	133.74	100.00	112.02	109.83	116.46	130.62	2.42
Flavisolibacter	0.00140	0.00468	62.82	100.00	84.60	116.03	108.65	103.24	100.00	110.78	91.39	88.72	102.69	3.86
Flavitalea	0.00000	0.00001	51.64	100.00	89.90	124.26	119.84	97.52	100.00	129.96	103.72	100.71	113.04	3.4
Flavobacterium	0.01213	0.02424	31.87	100.00	126.89	88.32	54.42	89.07	100.00	58.27	56.98	32.95	45.97	3.35
Frankineae bacterium (uncultured)	0.00831	0.01775	141.58	100.00	131.65	179.62	122.77	210.45	100.00	88.92	80.41	92.81	80.65	2.25
Gaiella	0.00000	0.00000	146.17	100.00	117.44	109.58	104.22	116.02	100.00	82.60	88.93	94.14	89.67	5
Galbitalea	0.01487	0.02869	100.06	100.00	127.08	95.27	115.29	105.58	100.00	96.88	59.94	57.97	69.38	3.34
Gemmata	0.00026	0.00142	84.75	100.00	92.65	76.46	79.03	66.07	100.00	111.46	112.90	119.21	96.25	4.5

	**P-values**	**FDR**	**t0**	**CFD_t1**	**CRD_t1**	**MYC_t1**	**NFB_t1**	**NFB+MYC_t1**	**CFD_t2**	**CRD_t2**	**MYC_t2**	**NFB_t2**	**NFB+MYC_t2**	**LDA score**
Gemmatimonadales bacterium (uncultured)	0.03593	0.05566	121.64	100.00	85.32	62.41	69.15	58.37	100.00	114.62	119.24	137.75	106.91	3.34
Gemmatimonas	0.00026	0.00143	57.15	100.00	82.77	122.26	121.57	115.15	100.00	113.32	89.98	80.59	97.97	3.92
Gemmatirosa	0.01892	0.03391	60.38	100.00	96.61	111.24	89.95	111.77	100.00	70.06	78.37	52.55	105.50	2.06
Geoalkalibacter	0.00200	0.00618	842.28	100.00	106.57	216.80	185.47	265.31	100.00	71.21	112.69	164.59	126.51	2.99
Glycomyces	0.00596	0.01356	57.21	100.00	97.28	44.33	66.71	48.69	100.00	25.88	38.60	32.39	39.27	2.57
Halobacillus	0.00000	0.00000	0.68	100.00	40.01	161.18	186.35	18.24	100.00	178.33	108.67	201.10	158.48	3.51
Hassallia	0.00000	0.00004	23.86	100.00	205.75	117.60	227.14	139.72	100.00	147.64	79.98	69.33	49.03	3.61

Herbinix	0.00233	0.00684	168.54	100.00	115.00	100.53	116.03	157.90	100.00	99.67	89.41	106.53	119.17	2.85
Herpetosiphon	0.01505	0.02893	61.77	100.00	107.90	131.76	103.31	92.22	100.00	70.19	68.31	61.96	83.54	3.86
Hirschia	0.00367	0.00982	31.99	100.00	144.85	114.40	189.50	120.75	100.00	300.83	111.05	82.21	88.80	3.72
Hungateiclostridium	0.03272	0.05161	195.28	100.00	109.75	168.26	133.97	199.49	100.00	129.12	77.54	103.10	238.87	2.01
Hyaloperonospora arabidopsidis	0.00001	0.00008	20.19	100.00	82.32	81.99	65.01	71.64	100.00	78.20	68.75	82.99	61.53	2.4
Hymenobacter	0.00542	0.01270	122.09	100.00	150.17	309.84	214.28	277.36	100.00	119.60	131.05	96.00	201.46	2.34
Iamia	0.01779	0.03250	103.85	100.00	110.70	99.64	100.66	82.56	100.00	76.88	66.41	68.22	67.08	3.64
Ideonella	0.00911	0.01899	28.19	100.00	247.07	109.35	136.73	78.72	100.00	119.45	118.14	87.42	93.44	3.06
Ilumatobacter	0.01706	0.03174	124.99	100.00	124.36	91.50	94.54	88.19	100.00	66.13	81.99	79.55	79.63	3.44
Inquilinus	0.00425	0.01080	43.79	100.00	119.19	67.39	73.56	159.74	100.00	377.13	175.06	133.32	142.83	2.71
Jahnella	0.00789	0.01691	206.20	100.00	247.29	177.94	386.14	203.78	100.00	989.05	932.05	84.63	239.04	2.87
Knoellia	0.00028	0.00150	110.71	100.00	95.39	106.75	111.40	120.87	100.00	86.57	86.93	86.62	106.19	4.3
Kribbella	0.01562	0.02987	141.16	100.00	116.14	99.47	103.13	105.02	100.00	83.50	89.27	87.40	83.03	2.91
Lacibacter	0.01648	0.03088	42.04	100.00	76.09	94.90	67.36	56.50	100.00	72.93	68.45	59.21	83.92	3.28
Lacunisphaera	0.00008	0.00054	47.92	100.00	169.27	120.55	123.97	92.84	100.00	157.16	58.75	67.62	45.95	3.27
Lapillicoccus	0.01963	0.03465	103.25	100.00	124.04	142.44	138.40	156.76	100.00	68.48	86.53	78.87	125.82	2.47
Lechevalieria	0.00000	0.00002	58.17	100.00	108.83	69.54	74.10	56.45	100.00	63.58	54.63	66.08	62.60	3.91

	P-values	FDR	t0	CFD_t1	CRD_t1	MYC_t1	NFB_t1	NFB+MYC_t1	CFD_t2	CRD_t2	MYC_t2	NFB_t2	NFB+MYC_t2	LDA score
Legionella	0.00008	0.00054	38.26	100.00	132.48	100.82	111.00	108.80	100.00	112.86	109.09	94.27	88.53	3.07
Leptolyngbya EcFYyyy 00	0.00009	0.00063	3.60	100.00	191.42	354.67	391.25	323.15	100.00	130.06	281.78	295.22	194.91	4.61
Longimicrobium	0.00692	0.01510	34.28	100.00	86.48	140.86	125.36	138.68	100.00	212.28	231.13	163.67	152.32	2.98
Luedemannella	0.00003	0.00027	224.31	100.00	139.02	109.39	111.46	183.16	100.00	81.86	94.79	101.95	82.85	3.47
Luteimonas	0.00146	0.00479	38.29	100.00	134.53	54.76	83.14	48.63	100.00	66.05	48.42	42.76	41.07	2.88
Luteolibacter	0.00907	0.01899	46.79	100.00	127.34	99.74	103.85	82.68	100.00	83.68	60.67	64.18	40.61	4.23
Lysinimonas	0.00915	0.01900	63.15	100.00	115.90	114.98	87.63	101.01	100.00	67.68	72.31	55.27	73.50	2.71
Lysobacter	0.00463	0.01133	70.99	100.00	91.90	114.75	98.60	97.13	100.00	85.89	69.87	63.73	85.12	3.9
Marisediminicola	0.01599	0.03026	141.87	100.00	175.95	229.09	194.55	301.69	100.00	101.25	119.06	73.68	148.19	2.18

Marmoricola	0.00516	0.01234	125.25	100.00	91.70	103.75	101.98	106.83	100.00	109.27	87.36	84.62	101.76	4.1
Massilia	0.01029	0.02095	100.16	100.00	95.11	132.90	127.02	151.54	100.00	104.12	90.88	70.22	125.84	4.26
Meiothermus	0.01882	0.03391	0.30	100.00	38.86	28.25	92.45	44.39	100.00	119.17	143.19	241.87	104.53	2.62
Mesorhizobium	0.00033	0.00168	52.85	100.00	151.99	115.13	138.25	119.05	100.00	126.20	95.67	95.71	99.80	4.14
Methylobacillus	0.00000	0.00000	5.79	100.00	107.47	110.75	30.41	44.34	100.00	29.17	49.87	23.09	52.78	3.42
Methyloceanibacter	0.02824	0.04566	145.79	100.00	98.82	87.89	86.21	69.16	100.00	117.82	142.43	133.41	91.59	2.21
Methylocella	0.00140	0.00468	51.70	100.00	172.00	151.11	200.09	145.65	100.00	116.65	98.56	100.05	136.05	2.38
Methylopila	0.01773	0.03250	77.00	100.00	330.40	178.52	154.59	113.78	100.00	529.03	290.52	129.42	327.15	2.66
Methylorosula	0.00343	0.00929	74.80	100.00	96.72	134.78	104.48	125.82	100.00	113.38	94.58	96.00	96.34	2.58
Methylotenera	0.00001	0.00008	29.84	100.00	103.32	114.02	87.40	80.17	100.00	36.19	48.12	33.27	110.41	3.82
Microbacterium	0.00156	0.00502	54.26	100.00	85.42	85.29	69.33	58.39	100.00	60.49	54.07	37.36	61.59	3.96
Micropepsis	0.00002	0.00024	0.97	100.00	220.64	81.01	194.29	94.79	100.00	353.53	138.50	90.07	42.87	2.86
Microvirga	0.00292	0.00823	88.56	100.00	86.27	131.31	126.10	130.93	100.00	127.78	100.71	107.14	115.58	4.58
Mitsuaria	0.00000	0.00002	4.05	100.00	183.02	64.83	107.85	52.00	100.00	21.80	125.31	51.11	27.27	4.11
Mucilaginibacter	0.00012	0.00075	151.87	100.00	117.77	125.08	130.84	144.16	100.00	147.74	63.00	94.71	39.10	2.77
	**P-values**	**FDR**	**t0**	**CFD_t1**	**CRD_t1**	**MYC_t1**	**NFB_t1**	**NFB+MYC_t1**	**CFD_t2**	**CRD_t2**	**MYC_t2**	**NFB_t2**	**NFB+MYC_t2**	**LDA score**
Mycobacterium	0.00000	0.00004	123.61	100.00	118.80	107.17	110.55	110.31	100.00	117.40	92.26	100.90	92.28	3.78
Nakamurella	0.02125	0.03693	123.49	100.00	114.11	135.28	153.23	195.26	100.00	86.75	99.14	95.11	111.40	2.72
Nannocystis	0.01458	0.02833	77.54	100.00	125.97	167.13	210.29	133.26	100.00	123.03	77.76	102.63	74.29	2.55
Niastella	0.00004	0.00035	53.25	100.00	105.22	91.35	122.00	68.05	100.00	94.79	94.98	91.94	80.03	3.24
Nibribacter	0.00774	0.01672	82.02	100.00	67.95	151.68	125.26	124.15	100.00	63.50	57.90	42.58	70.11	2.72
Nitratireductor	0.01276	0.02537	53.65	100.00	95.69	132.29	96.69	82.12	100.00	78.44	102.02	103.80	120.43	2.48
Nitrosomonas	0.00033	0.00168	18.75	100.00	80.65	106.48	133.36	121.63	100.00	133.55	161.85	137.42	127.64	2.94
Nitrosospira	0.03976	0.06052	57.97	100.00	112.10	116.84	175.66	161.21	100.00	113.83	150.39	129.48	106.55	3.09
Nocardioides	0.00014	0.00084	115.02	100.00	105.36	102.77	107.27	95.02	100.00	74.76	76.82	77.51	90.66	4.31
Nodosilinea PCC 7104	0.00011	0.00069	4.62	100.00	362.09	164.13	192.32	514.84	100.00	63.13	110.51	90.55	156.75	3.85
Nonomuraea	0.00531	0.01258	157.65	100.00	126.64	132.27	119.57	187.74	100.00	83.99	109.41	112.79	92.67	3.13

Nordella	0.00009	0.00063	76.93	100.00	86.59	76.71	85.10	67.29	100.00	122.40	116.88	132.49	101.50	3.96
Nostoc PCC 7524	0.01177	0.02368	44.82	100.00	106.18	71.73	59.95	89.90	100.00	68.91	220.67	106.38	151.94	2.51
Noviherbaspirillum	0.00380	0.00997	71.46	100.00	85.47	130.86	118.60	119.26	100.00	96.61	94.03	76.61	121.94	3.97
Novosphingobium	0.00157	0.00502	117.88	100.00	143.60	110.77	99.20	85.69	100.00	77.35	56.16	65.77	96.16	3.14
Ohtaekwangia	0.02306	0.03899	47.50	100.00	117.23	99.02	86.29	83.99	100.00	101.99	80.05	78.22	86.38	3.68
Oligoflexus	0.03917	0.05997	88.73	100.00	102.14	168.07	126.88	163.11	100.00	97.54	98.66	100.99	131.31	3.05
Oscillatoria SAG 1459 8	0.00208	0.00634	4.75	100.00	185.52	170.29	227.67	137.80	100.00	90.01	158.96	182.64	223.70	2.65
Oscillochloris	0.01618	0.03042	30.74	100.00	123.01	108.36	73.38	97.06	100.00	69.32	65.19	74.79	77.98	2.35
Paenarthrobacter	0.00150	0.00491	77.60	100.00	58.64	78.73	67.79	69.00	100.00	49.11	74.66	58.16	98.09	3.75
Paenisporosarcina	0.01173	0.02368	120.77	100.00	92.98	92.87	83.67	128.36	100.00	145.45	110.13	129.22	133.97	3.5
Panacagrimonas	0.00363	0.00975	21.25	100.00	98.70	127.46	94.06	67.99	100.00	60.78	90.81	47.81	66.98	2.8
Parasegetibacter	0.00005	0.00036	49.09	100.00	105.37	142.25	112.65	151.48	100.00	87.96	104.30	78.10	135.02	3.27
Parviterribacter	0.00672	0.01496	107.23	100.00	103.23	110.28	104.37	105.77	100.00	92.62	83.27	81.12	87.45	3.06
Paucibacter	0.00958	0.01982	35.37	100.00	71.54	92.25	73.55	83.85	100.00	102.32	87.04	113.07	112.75	2.71
	**P-values**	**FDR**	**t0**	**CFD_t1**	**CRD_t1**	**MYC_t1**	**NFB_t1**	**NFB+MYC_t1**	**CFD_t2**	**CRD_t2**	**MYC_t2**	**NFB_t2**	**NFB+MYC_t2**	**LDA score**
Pedinomonas minor	0.02292	0.03888	74.96	100.00	101.61	189.89	184.45	192.23	100.00	89.31	101.76	140.58	126.07	2.52
Pedobacter	0.00003	0.00027	68.34	100.00	87.52	118.23	108.68	128.85	100.00	98.99	90.63	86.97	111.70	3.62
Pedococcus Phycicoccus	0.00101	0.00368	112.66	100.00	91.30	91.22	94.26	78.23	100.00	61.43	67.18	77.29	93.92	2.93
Pedomicrobium	0.00016	0.00090	139.97	100.00	103.89	94.25	102.06	99.28	100.00	116.07	119.05	127.99	91.25	3.78
Pelomonas	0.00023	0.00125	22.55	100.00	159.44	120.99	85.80	98.27	100.00	46.33	40.67	47.02	80.94	2.86
Pelosinus	0.00030	0.00155	197.65	100.00	100.04	92.55	89.78	144.36	100.00	116.40	96.31	122.13	86.79	2.89
Peredibacter	0.00041	0.00193	51.66	100.00	85.27	106.68	112.31	105.97	100.00	94.72	86.59	73.89	85.68	3.04
Phenylobacterium	0.00000	0.00001	112.05	100.00	131.90	123.51	110.05	118.19	100.00	99.06	88.67	80.02	113.41	3.08
Pirellula	0.00055	0.00235	74.49	100.00	85.00	74.99	77.49	53.91	100.00	106.27	103.42	103.88	85.06	4.57
Piscinibacter	0.00093	0.00340	49.11	100.00	89.06	97.45	75.74	96.85	100.00	71.27	76.32	82.77	103.92	2.75
Planctomicrobium	0.00141	0.00468	53.48	100.00	81.78	69.25	61.67	42.83	100.00	81.15	75.60	73.80	57.99	2.9
Planctomycetaceae bacterium (uncultured)	0.00291	0.00823	62.17	100.00	91.81	90.08	87.37	53.17	100.00	101.20	211.54	183.31	96.37	2.05

	P-values	FDR	t0	CFD_t1	CRD_t1	MYC_t1	NFB_t1	NFB+MYC_t1	CFD_t2	CRD_t2	MYC_t2	NFB_t2	NFB+MYC_t2	LDA score
Planctomycetales bacterium (uncultured)	0.00001	0.00015	66.24	100.00	120.79	101.48	103.77	59.50	100.00	118.44	101.28	117.37	81.33	3.11
Planctomycete (uncultured)	0.00005	0.00041	53.62	100.00	75.36	96.46	95.67	79.42	100.00	108.99	88.24	85.58	83.22	4.31
Planctomycete WY108	0.00543	0.01270	28.02	100.00	57.24	122.71	82.53	75.53	100.00	83.16	77.73	89.21	98.94	2.16
Planctopirus	0.00001	0.00013	23.05	100.00	128.58	74.80	134.79	81.64	100.00	157.22	85.86	83.17	83.70	3.4
Planifilum	0.02763	0.04495	278.65	100.00	203.12	160.59	139.17	229.71	100.00	121.74	136.26	103.40	124.96	2.96
Planoglabratella opercularis	0.00541	0.01270	19.24	100.00	94.89	129.73	151.86	121.49	100.00	85.80	106.94	119.12	128.36	2.56
Polyangiaceae bacterium (uncultured)	0.00321	0.00890	28.26	100.00	50.94	77.22	51.64	48.59	100.00	151.66	95.95	102.68	55.73	2.29
Polyangium brachysporum group	0.00000	0.00000	33.86	100.00	85.79	115.99	87.04	59.93	100.00	116.26	54.32	74.12	73.16	3.66
Polycyclovorans	0.02839	0.04576	70.96	100.00	127.94	129.32	95.84	128.28	100.00	83.91	106.58	61.76	33.62	3.39
Pontibacter	0.00072	0.00276	56.91	100.00	85.35	132.54	121.50	147.26	100.00	84.84	85.91	80.03	96.78	3.72
Promicromonospora	0.01565	0.02987	192.40	100.00	156.00	74.89	99.11	118.69	100.00	115.87	66.31	51.40	54.83	3.19
Prosthecomicrobium	0.00299	0.00839	40.56	100.00	65.46	101.19	98.54	105.34	100.00	87.70	53.01	65.61	87.11	2.46
Pseudarthrobacter	0.02186	0.03769	78.22	100.00	76.26	131.71	98.48	108.84	100.00	71.56	93.26	75.33	138.47	3.4
Pseudenhygromyxa	0.03489	0.05439	40.62	100.00	128.89	134.75	136.17	170.12	100.00	200.38	154.89	177.66	113.84	2.36
Pseudoduganella	0.00011	0.00069	14.34	100.00	82.45	165.08	73.07	41.17	100.00	80.24	95.95	63.16	45.58	3.5
Pseudoflavitalea	0.00776	0.01672	66.48	100.00	112.18	77.67	106.57	134.46	100.00	308.41	183.39	218.32	135.56	3.16
Pseudolabrys	0.00116	0.00412	67.98	100.00	163.18	73.05	129.34	73.61	100.00	110.37	92.09	86.17	78.56	3.96
Pseudomuriella schumacherensis	0.00011	0.00069	9.06	100.00	91.09	106.25	100.33	95.91	100.00	164.37	193.50	312.29	290.11	3.45
Pseudonocardia	0.00687	0.01510	117.06	100.00	110.78	118.00	112.99	145.57	100.00	91.26	95.18	119.51	95.69	3.12
Pseudorhodoplanes	0.00166	0.00519	69.31	100.00	109.16	90.78	125.62	77.55	100.00	150.37	126.23	134.51	109.38	3.02
Pseudoxanthomonas	0.00079	0.00298	48.42	100.00	163.41	91.91	102.87	79.30	100.00	75.59	72.67	44.86	59.03	3.92
Qipengyuania	0.00000	0.00001	33.79	100.00	85.69	112.84	84.54	79.55	100.00	64.50	67.11	61.85	100.46	3.74
Ramlibacter	0.00027	0.00143	58.69	100.00	86.82	129.60	122.29	120.25	100.00	101.99	82.97	72.59	91.70	4.02
Reyranella	0.00109	0.00391	68.10	100.00	119.11	87.18	95.30	80.40	100.00	128.59	121.77	120.93	90.54	3.52
Rhizobacter	0.00054	0.00233	57.93	100.00	111.73	110.78	104.41	111.90	100.00	88.65	81.22	76.77	96.62	3.89
Rhodanobacter	0.00000	0.00000	1.02	100.00	314.56	97.41	221.09	137.83	100.00	298.67	97.46	86.93	68.93	3.55

Rhodobacter	0.00003	0.00029	45.46	100.00	150.64	186.75	180.28	176.86	100.00	59.64	67.56	69.21	108.80	3.37
Rhodococcus	0.02581	0.04266	155.13	100.00	64.09	69.24	88.66	84.43	100.00	102.86	57.65	67.36	91.95	2.88
Rhodocytophaga	0.01730	0.03195	51.46	100.00	89.16	111.02	107.92	125.10	100.00	98.13	118.50	90.48	115.32	3.17
Rhodoferax	0.00003	0.00029	50.55	100.00	169.32	105.07	172.84	147.20	100.00	108.58	88.02	72.70	96.84	2.85
Rhodopirellula	0.00523	0.01246	51.71	100.00	84.12	103.97	102.21	90.27	100.00	114.02	84.06	87.48	88.85	3.51
Rhodoplanes	0.00246	0.00714	79.79	100.00	92.83	76.09	86.50	65.65	100.00	105.57	116.40	123.29	89.17	3.66
Rhodopseudomonas	0.00001	0.00011	10.94	100.00	187.17	115.45	206.91	139.57	100.00	196.09	78.53	92.78	108.60	2.85
Roseisolibacter	0.00233	0.00684	81.26	100.00	137.77	141.25	119.87	153.86	100.00	106.89	91.08	67.00	96.22	2.67
Roseomonas	0.00651	0.01456	68.56	100.00	92.05	134.49	135.17	150.06	100.00	110.24	97.59	93.02	110.06	3.07
Rubellimicrobium	0.00020	0.00112	54.53	100.00	83.76	143.95	139.60	153.73	100.00	87.86	86.94	75.76	109.51	3.96
Rubrobacter	0.01717	0.03182	107.85	100.00	105.55	124.13	124.51	135.61	100.00	108.38	107.40	115.26	116.67	4.48
Rubrobacterales bacterium (uncultured)	0.00000	0.00000	142.41	100.00	112.07	92.87	96.22	89.09	100.00	82.56	82.42	94.51	84.49	4.35

	**P-values**	**FDR**	**t0**	**CFD_t1**	**CRD_t1**	**MYC_t1**	**NFB_t1**	**NFB+MYC_t1**	**CFD_t2**	**CRD_t2**	**MYC_t2**	**NFB_t2**	**NFB+MYC_t2**	**LDA score**
Rubrobacteria bacterium (uncultured)	0.00000	0.00000	134.25	100.00	122.64	106.69	103.07	107.67	100.00	83.17	89.26	97.02	90.80	3.86
Ruminiclostridium	0.00036	0.00177	269.96	100.00	125.16	149.52	134.56	228.75	100.00	110.41	114.36	135.95	109.35	3.1
Saccharothrix	0.01654	0.03088	57.17	100.00	73.83	78.65	70.99	101.86	100.00	66.12	70.52	61.16	84.10	3
Salinispora	0.00156	0.00502	165.30	100.00	110.44	97.97	118.58	110.86	100.00	118.27	130.88	195.50	106.60	2.55
Sandaracinus	0.01947	0.03461	90.27	100.00	113.18	122.47	98.88	107.06	100.00	132.32	105.50	110.06	86.74	2.87
Schlesneria	0.00001	0.00013	2.89	100.00	155.70	115.44	194.61	55.43	100.00	73.06	49.00	43.23	57.44	2.53
Segetibacter	0.00109	0.00391	79.90	100.00	60.79	97.32	100.30	81.24	100.00	79.32	77.26	87.22	114.92	2.76
Shimazuella	0.00369	0.00983	168.86	100.00	140.91	130.88	133.12	209.36	100.00	85.16	108.24	93.28	90.62	3.02
Shinella	0.00006	0.00047	15.83	100.00	158.21	75.27	66.07	50.63	100.00	55.49	77.33	54.78	49.18	3.67
Sideroxydans	0.00040	0.00187	2434.65	100.00	165.76	216.94	225.28	299.77	100.00	85.18	216.62	508.02	77.20	3.21
Simplicispira	0.00000	0.00001	5.80	100.00	262.50	88.50	200.39	110.94	100.00	286.09	114.73	60.13	49.29	2.86
Skermanella	0.02010	0.03512	106.83	100.00	109.38	135.70	160.14	206.92	100.00	98.83	102.19	119.86	124.88	4.42
Solibacillus	0.02247	0.03851	209.79	100.00	157.84	135.71	125.72	188.34	100.00	96.74	84.17	59.00	115.33	2.25
Solimonas	0.00006	0.00042	28.36	100.00	187.09	109.74	72.95	67.35	100.00	183.91	55.71	53.00	35.40	2.76

Solirubrobacter	0.00002	0.00020	128.82	100.00	110.10	108.46	107.11	111.63	100.00	93.48	93.45	94.78	109.31	3.98
Sorangium	0.00128	0.00440	203.32	100.00	181.93	120.12	226.11	97.54	100.00	134.06	80.20	58.56	81.75	3.1
Sphingoaurantiacus	0.00002	0.00024	74.29	100.00	98.04	138.19	121.09	145.29	100.00	90.94	72.41	77.96	126.68	3
Sphingobium	0.00000	0.00004	7.63	100.00	117.25	148.24	103.56	72.78	100.00	50.33	52.51	34.76	56.87	4.63
Sphingomonas	0.00001	0.00014	87.63	100.00	101.17	114.62	109.22	102.87	100.00	96.81	79.26	79.47	99.88	4.59
Sphingopyxis	0.00000	0.00000	24.99	100.00	189.45	104.10	103.86	64.23	100.00	44.09	44.57	38.97	44.66	3.72
Spirochaeta	0.00085	0.00314	833.59	100.00	70.11	233.44	132.33	73.13	100.00	95.57	81.40	93.70	160.60	2.91
Sporosarcina	0.00060	0.00244	137.80	100.00	103.47	122.83	101.70	152.79	100.00	106.51	109.22	122.83	140.21	3.08
Stenotrophomonas	0.02444	0.04093	147.46	100.00	74.10	17.42	76.55	14.35	100.00	43.74	30.17	15.59	6.81	2.78
Steroidobacter	0.00120	0.00425	128.01	100.00	115.25	102.05	111.38	127.25	100.00	118.63	119.93	129.38	107.46	3.82
Streptomyces	0.00042	0.00194	73.06	100.00	101.53	77.19	80.55	82.68	100.00	87.18	73.06	83.63	88.38	4.12
	**P-values**	**FDR**	**t0**	**CFD_t1**	**CRD_t1**	**MYC_t1**	**NFB_t1**	**NFB+MYC_t1**	**CFD_t2**	**CRD_t2**	**MYC_t2**	**NFB_t2**	**NFB+MYC_t2**	**LDA score**
Streptosporangium	0.02289	0.03888	131.57	100.00	146.67	94.38	160.29	180.28	100.00	183.74	129.49	133.72	151.67	2.49
Symbiobacterium	0.00616	0.01394	185.28	100.00	134.25	113.14	107.03	156.05	100.00	87.43	117.40	109.03	145.63	2.77
Synechococcus IR11	0.00009	0.00063	4.80	100.00	409.85	333.72	372.42	453.42	100.00	299.64	107.29	303.15	349.12	3.41
Syntrophobacter	0.02445	0.04093	246.20	100.00	60.26	85.20	76.13	115.06	100.00	101.19	95.98	115.96	110.48	2.77
Tahibacter	0.01475	0.02857	30.99	100.00	76.58	61.16	212.04	115.18	100.00	108.17	52.22	38.62	47.83	3.16
Telmatocola	0.04084	0.06197	63.97	100.00	62.45	123.96	79.37	110.79	100.00	96.99	65.25	81.31	75.76	2.39
Tepidisphaera	0.01603	0.03026	50.66	100.00	75.71	117.60	108.85	81.27	100.00	106.85	95.20	73.69	76.01	3.21
Terribacillus	0.00621	0.01395	160.42	100.00	138.95	130.11	104.80	179.93	100.00	148.92	132.00	162.73	475.11	3.01
Terrimicrobium	0.00022	0.00122	51.60	100.00	163.61	100.30	145.58	145.70	100.00	76.97	74.91	73.52	56.70	2.82
Terrimonas	0.00018	0.00105	57.23	100.00	92.58	90.36	101.02	72.46	100.00	124.76	99.33	100.66	95.49	3.87
Thermoactinomyces	0.03773	0.05811	253.59	100.00	165.99	139.46	108.50	156.91	100.00	142.78	148.09	114.89	92.94	2.72
Thermoflavimicrobium	0.00439	0.01097	257.93	100.00	209.60	168.42	129.66	297.75	100.00	114.43	120.73	127.91	106.83	2.73
Truepera	0.00000	0.00000	39.74	100.00	66.98	104.66	66.23	95.54	100.00	137.36	117.83	78.43	138.40	3.5
Tumebacillus	0.00057	0.00238	219.44	100.00	196.33	117.84	135.04	220.85	100.00	79.84	127.90	118.06	96.51	3.76
Tychonema CCAP 1459 11B	0.01920	0.03424	27.22	100.00	90.19	63.97	133.65	168.82	100.00	67.76	179.90	351.08	231.45	4.18

Vampirovibrio	0.00033	0.00167	4.82	100.00	72.16	74.52	73.85	42.43	100.00	106.87	86.84	105.09	103.43	2.42
Variovorax	0.00056	0.00237	67.59	100.00	110.82	75.98	83.65	87.16	100.00	70.99	74.00	68.20	65.06	3.69
Verrucomicrobia bacterium (uncultured)	0.00010	0.00067	28.85	100.00	77.53	100.56	77.53	54.30	100.00	88.00	66.96	66.54	75.82	2.63
Verrucomicrobiales bacterium (uncultured)	0.00006	0.00042	36.55	100.00	108.20	103.23	113.86	91.24	100.00	62.47	73.19	82.92	54.95	2.54
Virgibacillus	0.00003	0.00027	10.17	100.00	42.69	170.77	82.62	17.87	100.00	83.64	103.50	154.34	89.50	3.39
Vischeria sp CAUP Q 202	0.00840	0.01788	31.15	100.00	135.70	126.19	72.01	214.15	100.00	78.19	118.02	53.70	144.57	2.54
Yonghaparkia	0.00000	0.00000	11.44	100.00	95.12	128.12	84.55	55.40	100.00	62.85	69.17	53.12	96.94	3.61
Zavarzinella	0.00465	0.01133	71.74	100.00	89.12	88.21	78.58	55.73	100.00	122.17	115.43	114.00	107.80	3.06

Table S4. Signature associated to each treatment (Label). Data grouped by label. Percentual standardization of values vs. CRD. Negative differences greater than 10% are highlighted in red, while positive differences greater than 10% are highlighted in green.

	P-values	FDR	t0	CFD_t1	CRD_t1	MYC_t1	NFB_t1	NFB+MYC_t1	CFD_t2	CRD_t2	MYC_t2	NFB_t2	NFB+MYC_t2	LDA score
Abditibacterium	0.00126	0.00440	79.98	142.99	100.00	168.61	134.02	121.10	127.64	100.00	98.55	64.08	107.78	2.58
Acidibacter	0.00018	0.00101	69.06	94.61	100.00	94.75	107.86	103.39	76.83	100.00	71.49	93.26	74.95	3.92
Acidimicrobiia bacterium (uncultured)	0.00000	0.00002	147.98	83.67	100.00	79.88	88.27	90.15	117.91	100.00	106.77	105.06	107.55	4.07
Acidobacteria bacterium (uncultured)	0.01339	0.02642	122.68	117.51	100.00	91.78	100.11	90.64	92.28	100.00	111.29	114.91	90.74	4.35
Acidobacteriaceae bacterium (uncultured)	0.00227	0.00674	118.83	113.54	100.00	94.79	103.35	93.70	88.42	100.00	104.98	109.12	85.94	4.5
Acidobacteriales bacterium (uncultured)	0.00013	0.00081	153.39	107.67	100.00	90.64	99.01	94.22	82.52	100.00	109.74	123.68	81.12	4.3
Acidothermus	0.00004	0.00032	601.98	77.30	100.00	53.68	96.44	94.33	166.15	100.00	107.38	103.60	135.13	2.84
Acidovorax	0.00000	0.00002	10.19	70.67	100.00	104.24	81.23	30.92	225.56	100.00	157.56	112.08	174.86	3.44
Actinobacterium (uncultured)	0.00002	0.00020	107.69	84.84	100.00	86.09	82.07	66.93	104.11	100.00	95.61	98.13	80.72	4.16
Actinocorallia	0.00038	0.00182	109.39	67.73	100.00	89.82	84.67	47.12	70.81	100.00	61.93	115.05	45.20	3.03
Actinomadura	0.00081	0.00303	132.36	102.65	100.00	112.15	108.18	134.93	95.44	100.00	90.38	110.16	81.77	2.83
Actinomycetales bacterium (uncultured)	0.00000	0.00000	92.60	88.45	100.00	88.04	89.16	86.45	126.83	100.00	105.29	120.20	114.40	3.45
Actinomycetospora	0.01895	0.03391	120.39	73.13	100.00	50.66	114.91	164.54	105.89	100.00	96.57	151.39	87.83	2.4
Actinopolymorpha	0.03957	0.06041	194.17	85.05	100.00	84.37	85.99	141.39	194.33	100.00	186.53	173.14	147.65	2.44

Actinotalea	0.03516	0.05463	114.36	103.53	100.00	104.98	88.33	128.38	125.23	100.00	112.59	99.33	149.49	2.53
Adhaeribacter	0.00203	0.00625	91.91	112.51	100.00	155.66	143.63	157.60	101.09	100.00	104.94	94.85	134.69	3.72
Aeromicrobium	0.00395	0.01016	50.86	109.10	100.00	134.57	117.51	110.37	118.97	100.00	74.49	60.32	70.02	4.53
Allorhizobium, Neorhizobium, Pararhizobium, Rhizobium	0.00127	0.00440	54.71	92.20	100.00	98.74	128.31	130.56	113.26	100.00	96.99	80.85	86.97	4.46
Alsobacter	0.01812	0.03278	94.64	97.77	100.00	158.55	148.45	158.91	82.43	100.00	94.76	88.30	119.33	2.87
Altererythrobacter	0.00068	0.00265	30.53	63.27	100.00	68.14	70.50	53.18	107.33	100.00	74.18	76.71	95.92	3.63
Amaricoccus	0.00886	0.01862	78.51	86.32	100.00	113.28	124.33	178.45	110.29	100.00	101.48	110.60	128.11	3.44
Aminobacter	0.00000	0.00001	65.90	131.83	100.00	519.40	159.50	431.39	67.21	100.00	111.18	88.50	101.75	3.47

	P-values	FDR	t0	CFD_t1	CRD_t1	MYC_t1	NFB_t1	NFB+MYC_t1	CFD_t2	CRD_t2	MYC_t2	NFB_t2	NFB+MYC_t2	LDA score
Ammoniphilus	0.00219	0.00665	101.14	72.28	100.00	83.40	78.13	135.52	89.91	100.00	68.58	74.10	82.56	3.11
Amycolatopsis	0.00000	0.00000	39.38	144.84	100.00	102.99	42.24	27.70	329.52	100.00	142.34	165.89	206.54	3.63
Anaerobacterium	0.01310	0.02594	280.95	96.22	100.00	121.78	84.36	118.43	75.33	100.00	91.32	94.81	103.25	2.75
Anaerolinea	0.00142	0.00468	407.04	111.95	100.00	131.15	127.41	262.95	135.38	100.00	144.11	158.55	132.87	3.78
Anaeromyxobacter	0.00344	0.00929	163.43	96.18	100.00	88.31	110.55	140.58	100.44	100.00	121.16	110.85	105.73	3.58
Anaeromyxobacter dehalogenans	0.03063	0.04861	77.99	80.15	100.00	202.05	144.32	246.16	45.07	100.00	77.53	46.71	63.14	2.08
Aquaspirillum arcticum group	0.02385	0.04018	49.54	88.75	100.00	194.74	216.98	184.24	103.97	100.00	66.01	77.52	83.14	2.15
Aquicella	0.01029	0.02095	32.51	148.82	100.00	87.42	86.99	65.47	90.75	100.00	85.90	100.78	64.73	2.67
Aquipuribacter	0.00986	0.02031	74.71	153.88	100.00	154.72	140.71	157.36	82.77	100.00	91.47	85.29	94.20	2.28
Aquisphaera	0.02632	0.04322	164.33	83.09	100.00	77.51	82.63	109.36	121.71	100.00	148.84	136.49	97.13	2.82
Arenimonas	0.00034	0.00170	32.34	86.78	100.00	83.03	77.93	74.98	132.76	100.00	97.73	55.80	108.34	3.45
Aridibacter	0.00691	0.01510	75.77	130.40	100.00	125.76	133.43	121.13	102.26	100.00	85.11	96.76	116.90	3.18
Armatimonadetes bacterium (uncultured)	0.00480	0.01163	94.50	101.41	100.00	128.06	112.70	114.35	83.98	100.00	97.48	99.74	92.57	3.16
Armatimonas	0.00197	0.00614	9.27	56.45	100.00	89.48	80.96	57.50	195.24	100.00	193.63	103.03	221.16	2.02
Arthrobacter	0.00735	0.01597	112.42	123.42	100.00	146.73	134.34	144.65	116.43	100.00	121.97	99.68	179.33	5.42
Asanoa	0.02186	0.03769	83.62	40.09	100.00	52.68	57.39	101.30	136.53	100.00	174.92	150.47	124.21	2.25
Asticcacaulis	0.00003	0.00027	9.91	38.91	100.00	37.32	41.11	31.53	49.05	100.00	72.69	38.31	27.60	3.19

	P-values	FDR	t0	CFD_t1	CRD_t1	MYC_t1	NFB_t1	NFB+MYC_t1	CFD_t2	CRD_t2	MYC_t2	NFB_t2	NFB+MYC_t2	LDA score
Azohydromonas	0.00000	0.00000	68.77	107.09	100.00	121.47	110.86	126.90	99.57	100.00	84.70	84.29	99.53	3.46
Azospira	0.00000	0.00004	0.75	46.11	100.00	86.42	79.93	35.58	119.42	100.00	34.91	13.08	10.33	2.8
Azospirillum	0.00428	0.01084	39.96	81.77	100.00	73.33	252.00	169.09	47.90	100.00	46.84	72.97	56.99	2.63
Bacteroidetes bacterium (uncultured)	0.00342	0.00929	47.58	111.53	100.00	113.98	121.58	99.71	107.71	100.00	76.58	69.32	67.68	2.75
Bauldia	0.01958	0.03465	45.41	68.47	100.00	61.69	96.45	67.59	55.87	100.00	54.15	51.44	39.58	3.22
Bdellovibrio	0.00000	0.00001	34.37	91.83	100.00	104.69	103.10	105.64	80.88	100.00	71.11	71.34	103.11	3.02
Blastocatella	0.00278	0.00802	71.24	117.47	100.00	128.91	119.88	99.38	107.51	100.00	103.71	114.06	112.51	3.49
Blastococcus	0.00575	0.01326	95.19	103.31	100.00	130.20	126.48	145.56	91.67	100.00	87.54	93.57	109.95	4.14
Blastopirellula	0.00005	0.00041	104.99	108.13	100.00	100.63	96.81	84.91	85.84	100.00	113.88	125.83	84.91	3.12
Bosea	0.00137	0.00465	52.02	95.74	100.00	120.46	130.21	160.50	93.19	100.00	88.26	68.24	85.43	3.65
Bradyrhizobium	0.00245	0.00714	65.13	84.42	100.00	77.31	95.77	86.66	94.59	100.00	100.34	109.78	90.72	3.94
Brevibacillus	0.02128	0.03693	135.27	72.41	100.00	86.23	84.39	121.10	73.11	100.00	70.83	70.12	65.25	2.85
Brevifollis	0.00001	0.00008	153.78	77.81	100.00	67.74	44.03	123.41	59.78	100.00	77.50	49.65	57.82	2.3
Brevundimonas	0.00001	0.00016	68.83	79.73	100.00	109.22	78.01	83.64	161.83	100.00	68.11	59.62	149.92	2.78
Burkholderia Caballeronia Paraburkholderia	0.02886	0.04623	135.14	69.90	100.00	79.06	76.17	122.11	103.56	100.00	48.28	43.99	56.57	3.43
Burkholderiales bacterium Beta 02	0.00162	0.00509	26.72	160.48	100.00	92.80	95.39	78.97	129.46	100.00	75.21	63.45	80.13	2.27
Caenimonas	0.00039	0.00184	68.30	112.17	100.00	142.82	140.10	150.13	106.09	100.00	80.44	75.99	107.80	4.02
Caldicoprobacter	0.00498	0.01197	152.50	99.87	100.00	118.43	82.97	144.66	89.79	100.00	84.59	90.49	101.49	2.75
Caldilineaceae bacterium (uncultured)	0.03295	0.05183	76.53	77.90	100.00	73.01	78.01	88.34	104.73	100.00	98.42	108.72	94.12	2.92
Candidatus Alysiosphaera	0.02903	0.04636	100.96	99.45	100.00	117.96	128.61	166.89	119.34	100.00	115.43	128.77	119.17	3.87
Candidatus Chloroploca	0.02749	0.04487	53.98	75.16	100.00	92.94	122.46	144.63	170.68	100.00	187.07	157.52	151.98	2.86
Candidatus Nomurabacteria bacterium GW2011 GWE1 32 28	0.02283	0.03888	47.25	130.05	100.00	144.31	126.19	143.63	54.87	100.00	33.10	33.22	30.60	2.08
Candidatus Udaeobacter	0.00406	0.01038	109.53	106.71	100.00	65.74	73.98	47.24	86.73	100.00	100.63	119.91	77.33	4.18
Candidatus Xiphinematobacter	0.00222	0.00669	138.97	125.88	100.00	100.09	109.53	90.82	84.25	100.00	112.06	129.26	82.76	4.71
Caulobacter	0.00000	0.00001	22.77	36.92	100.00	40.25	40.20	31.60	106.89	100.00	75.95	59.29	80.42	3.63

	P-values	FDR	t0	CFD_t1	CRD_t1	MYC_t1	NFB_t1	NFB+MYC_t1	CFD_t2	CRD_t2	MYC_t2	NFB_t2	NFB+MYC_t2	LDA score
Cellulomonas	0.00006	0.00045	30.14	37.18	100.00	52.49	87.79	65.59	88.08	100.00	99.11	72.11	102.42	3.38
Cellvibrio	0.00008	0.00055	16.99	42.85	100.00	45.97	36.99	44.22	112.19	100.00	77.66	54.48	93.84	3.64
Chelativorans	0.00000	0.00000	52.17	60.86	100.00	3047.37	135.78	3075.22	119.82	100.00	1044.06	89.83	895.62	3.57
Chlamydomonas reinhardtii	0.01368	0.02679	14.39	116.97	100.00	167.57	59.00	232.01	49.97	100.00	50.89	53.91	102.40	2.27
Chloroflexi bacterium (uncultured)	0.00000	0.00000	107.74	92.88	100.00	104.54	101.09	117.79	111.95	100.00	103.35	105.75	107.03	4.4
Chloronema	0.00068	0.00265	18.54	45.76	100.00	118.50	107.21	94.50	84.62	100.00	205.82	160.60	150.30	3.05
Chryseobacterium	0.02952	0.04699	73.97	52.95	100.00	18.06	19.65	13.86	184.08	100.00	316.46	185.31	340.54	2.42
Chthoniobacter	0.03489	0.05439	82.43	121.01	100.00	127.02	125.30	114.22	90.41	100.00	94.90	89.39	91.19	4.1
Chthonobacter	0.00444	0.01097	46.53	89.67	100.00	164.05	161.38	127.04	77.39	100.00	59.97	44.91	66.94	2.68
Chthonomonas	0.00051	0.00222	68.39	90.47	100.00	103.05	96.40	108.11	90.63	100.00	94.61	109.22	118.02	2.95
Clostridia bacterium (uncultured)	0.04274	0.06468	183.55	77.84	100.00	107.53	92.46	121.81	191.26	100.00	116.85	207.71	200.74	2.35
Clostridium sensu stricto 1	0.00618	0.01394	173.84	106.83	100.00	82.09	95.22	150.07	149.83	100.00	181.72	169.90	141.34	3.27
Clostridium sensu stricto 12	0.01778	0.03250	124.58	95.78	100.00	87.98	107.69	181.79	72.80	100.00	99.51	112.05	83.38	2.83
Clostridium sensu stricto 13	0.01969	0.03465	114.08	105.82	100.00	80.86	88.59	141.75	87.10	100.00	92.07	117.76	102.17	3.06
Clostridium sensu stricto 8	0.00443	0.01097	149.78	96.58	100.00	96.58	93.17	151.08	98.58	100.00	108.38	116.86	106.18	2.94
Cnuella	0.00159	0.00502	37.61	116.93	100.00	146.52	94.49	138.52	82.21	100.00	68.89	80.00	102.21	2.65
Cohnella	0.00057	0.00237	130.90	79.58	100.00	96.22	99.04	144.54	85.38	100.00	101.68	107.90	101.44	3.61
Comamonas	0.00001	0.00011	51.41	101.92	100.00	103.15	105.25	105.40	115.82	100.00	90.47	87.00	91.54	3.1
Conexibacter	0.00036	0.00177	92.09	87.40	100.00	100.40	93.98	92.77	139.56	100.00	99.75	105.71	124.22	3.23
Conexibacteraceae bacterium (uncultured)	0.00043	0.00196	140.96	89.26	100.00	102.56	101.27	57.77	170.98	100.00	129.88	172.51	135.39	2.49
Croceicoccus	0.02866	0.04605	14.82	55.84	100.00	92.40	83.57	72.13	87.65	100.00	73.87	66.76	91.98	2.5
Cupriavidus	0.00001	0.00011	66.24	93.96	100.00	76.72	97.19	199.98	49.95	100.00	57.51	57.46	64.27	3.59
Dactylosporangium	0.00047	0.00210	129.82	78.50	100.00	79.11	85.14	152.38	139.17	100.00	110.38	150.20	110.83	2.82
Deinococcus	0.00000	0.00001	3.14	146.90	100.00	130.67	74.53	166.96	161.41	100.00	43.55	23.81	49.10	3.53
delta proteobacterium WX81	0.03074	0.04864	241.24	145.36	100.00	116.80	102.96	128.47	99.29	100.00	113.43	123.55	77.82	2.3
Deltaproteobacteria bacterium GWC2 55 46	0.00375	0.00988	388.70	111.34	100.00	126.65	126.41	276.91	153.81	100.00	120.28	159.59	119.58	2.5

	P-values	FDR	t0	CFD_t1	CRD_t1	MYC_t1	NFB_t1	NFB+MYC_t1	CFD_t2	CRD_t2	MYC_t2	NFB_t2	NFB+MYC_t2	LDA score
Desmochloris halophila	0.00047	0.00210	3.92	185.88	100.00	113.66	94.76	117.26	97.33	100.00	87.25	102.27	101.56	3.36
Desulfohalotomaculum	0.01215	0.02424	132.01	75.98	100.00	85.36	120.48	192.75	90.44	100.00	86.84	129.05	86.36	2.11
Desulfosporosinus	0.00586	0.01341	219.63	83.43	100.00	93.59	112.01	164.32	73.57	100.00	84.78	76.53	90.99	2.35
Devosia	0.00000	0.00007	33.24	73.55	100.00	73.46	83.08	67.99	96.89	100.00	69.35	62.34	62.85	4.27
Dokdonella	0.00003	0.00029	0.01	39.18	100.00	52.91	111.96	20.56	36.38	100.00	34.50	36.15	54.38	2.64
Domibacillus	0.00066	0.00260	132.40	121.95	100.00	96.76	97.64	113.16	153.39	100.00	136.24	117.66	218.68	3.49
Dongia	0.00993	0.02038	137.74	108.84	100.00	80.23	88.39	84.88	79.29	100.00	89.93	103.56	82.91	3.64
Dyadobacter	0.00011	0.00069	36.97	79.17	100.00	87.53	66.38	39.08	268.03	100.00	304.42	84.82	128.67	3.9
Ellin517	0.00066	0.00261	44.38	139.11	100.00	96.88	93.37	64.08	81.97	100.00	79.98	81.02	78.47	3.87
Ellin6055	0.00009	0.00063	59.88	117.31	100.00	118.20	111.14	97.79	111.79	100.00	85.64	83.59	114.67	4.05
Ellin6067	0.01801	0.03278	66.24	106.14	100.00	117.41	124.04	90.61	90.18	100.00	73.57	58.76	74.43	3.86
Ensifer	0.00000	0.00002	70.32	108.20	100.00	103.25	96.79	102.90	96.02	100.00	110.65	102.76	100.19	3.85
Enterobacter	0.00443	0.01097	153.01	770.40	100.00	104.21	81.13	108.78	579.18	100.00	112.79	102.14	531.70	4.18
Erythrobacter	0.00076	0.00286	1.92	53.16	100.00	217.36	52.05	35.55	153.36	100.00	75.06	101.69	188.01	3.1
Ettlia pseudoalveolaris	0.01813	0.03278	19.67	117.41	100.00	182.92	142.88	230.14	147.80	100.00	79.76	62.61	613.52	3.23
Ferrovibrio	0.02576	0.04266	35.13	114.94	100.00	119.20	127.15	112.87	95.39	100.00	56.81	87.76	64.90	2.47
Ferruginibacter	0.00557	0.01292	83.12	118.66	100.00	133.00	137.16	131.27	99.94	100.00	101.33	96.02	110.71	3.37
Fervidicella	0.00681	0.01505	237.37	164.35	100.00	154.22	71.47	382.67	35.56	100.00	69.15	142.87	111.35	2.44
Fictibacillus	0.00004	0.00035	164.37	93.17	100.00	99.15	95.76	131.34	51.59	100.00	51.59	65.02	69.98	3.77
Fimbriiglobus	0.00031	0.00162	77.43	104.05	100.00	92.71	100.91	85.04	90.23	100.00	106.69	109.58	90.63	3.86
Flavihumibacter	0.00853	0.01806	73.76	113.89	100.00	150.42	149.21	152.31	89.27	100.00	98.04	103.96	116.60	2.42
Flavisolibacter	0.00140	0.00468	74.26	118.20	100.00	137.15	128.42	122.03	90.27	100.00	82.50	80.09	92.69	3.86
Flavitalea	0.00000	0.00001	57.44	111.23	100.00	138.22	133.30	108.47	76.94	100.00	79.81	77.49	86.98	3.4
Flavobacterium	0.01213	0.02424	25.12	78.81	100.00	69.60	42.89	70.19	171.63	100.00	97.79	56.55	78.90	3.35
Frankineae bacterium (uncultured)	0.00831	0.01775	107.55	75.96	100.00	136.44	93.26	159.86	112.46	100.00	90.43	104.38	90.70	2.25
Gaiella	0.00000	0.00000	124.46	85.15	100.00	93.30	88.74	98.78	121.06	100.00	107.66	113.96	108.55	5

Galbitalea	0.01487	0.02869	78.74	78.69	100.00	74.97	90.72	83.08	103.23	100.00	61.88	59.84	71.61	3.34
Gemmata	0.00026	0.00142	91.47	107.93	100.00	82.52	85.29	71.31	89.72	100.00	101.29	106.95	86.36	4.5
Gemmatimonadales bacterium (uncultured)	0.03593	0.05566	142.58	117.21	100.00	73.15	81.05	68.42	87.24	100.00	104.03	120.18	93.27	3.34
Gemmatimonas	0.00026	0.00143	69.05	120.82	100.00	147.72	146.89	139.13	88.25	100.00	79.40	71.12	86.45	3.92
Gemmatirosa	0.01892	0.03391	62.49	103.51	100.00	115.14	93.11	115.69	142.74	100.00	111.86	75.01	150.58	2.06
	**P-values**	**FDR**	**t0**	**CFD_t1**	**CRD_t1**	**MYC_t1**	**NFB_t1**	**NFB+MYC_t1**	**CFD_t2**	**CRD_t2**	**MYC_t2**	**NFB_t2**	**NFB+MYC_t2**	**LDA score**
Geoalkalibacter	0.00200	0.00618	790.36	93.84	100.00	203.43	174.04	248.95	140.43	100.00	158.26	231.15	177.66	2.99
Glycomyces	0.00596	0.01356	58.81	102.80	100.00	45.57	68.58	50.06	386.43	100.00	149.15	125.17	151.77	2.57
Halobacillus	0.00000	0.00000	1.71	249.96	100.00	402.89	465.81	45.60	56.08	100.00	60.94	112.77	88.87	3.51
Hassallia	0.00000	0.00004	11.60	48.60	100.00	57.16	110.40	67.91	67.73	100.00	54.17	46.96	33.21	3.61
Herbinix	0.00233	0.00684	146.56	86.96	100.00	87.41	100.90	137.30	100.33	100.00	89.71	106.88	119.56	2.85
Herpetosiphon	0.01505	0.02893	57.25	92.67	100.00	122.11	95.74	85.47	142.47	100.00	97.32	88.27	119.02	3.86
Hirschia	0.00367	0.00982	22.09	69.04	100.00	78.98	130.83	83.36	33.24	100.00	36.91	27.33	29.52	3.72
Hungateiclostridium	0.03272	0.05161	177.93	91.11	100.00	153.31	122.06	181.76	77.45	100.00	60.05	79.85	185.00	2.01
Hyaloperonospora arabidopsidis	0.00001	0.00008	24.53	121.47	100.00	99.59	78.97	87.03	127.89	100.00	87.92	106.13	78.69	2.4
Hymenobacter	0.00542	0.01270	81.30	66.59	100.00	206.33	142.69	184.70	83.61	100.00	109.57	80.26	168.44	2.34
Iamia	0.01779	0.03250	93.82	90.34	100.00	90.01	90.93	74.58	130.07	100.00	86.38	88.74	87.25	3.64
Ideonella	0.00911	0.01899	11.41	40.47	100.00	44.26	55.34	31.86	83.72	100.00	98.91	73.19	78.23	3.06
Ilumatobacter	0.01706	0.03174	100.51	80.41	100.00	73.58	76.02	70.92	151.21	100.00	123.98	120.29	120.40	3.44
Inquilinus	0.00425	0.01080	36.74	83.90	100.00	56.54	61.71	134.02	26.52	100.00	46.42	35.35	37.87	2.71
Jahnella	0.00789	0.01691	83.38	40.44	100.00	71.96	156.15	82.40	10.11	100.00	94.24	8.56	24.17	2.87
Knoellia	0.00028	0.00150	116.06	104.83	100.00	111.90	116.78	126.70	115.52	100.00	100.43	100.06	122.67	4.3
Kribbella	0.01562	0.02987	121.54	86.10	100.00	85.64	88.80	90.42	119.75	100.00	106.90	104.67	99.43	2.91
Lacibacter	0.01648	0.03088	55.26	131.43	100.00	124.73	88.53	74.25	137.11	100.00	93.85	81.18	115.06	3.28
Lacunisphaera	0.00008	0.00054	28.31	59.08	100.00	71.22	73.24	54.85	63.63	100.00	37.38	43.03	29.24	3.27
Lapillicoccus	0.01963	0.03465	83.24	80.62	100.00	114.83	111.58	126.37	146.02	100.00	126.35	115.18	183.72	2.47
Lechevalieria	0.00000	0.00002	53.45	91.89	100.00	63.90	68.09	51.87	157.28	100.00	85.93	103.93	98.46	3.91

Legionella	0.00008	0.00054	28.88	75.48	100.00	76.10	83.78	82.12	88.60	100.00	96.66	83.53	78.44	3.07
Leptolyngbya EcFYyyy 00	0.00009	0.00063	1.88	52.24	100.00	185.28	204.39	168.82	76.89	100.00	216.66	226.99	149.87	4.61
Longimicrobium	0.00692	0.01510	39.64	115.63	100.00	162.88	144.96	160.35	47.11	100.00	108.88	77.10	71.76	2.98
Luedemannella	0.00003	0.00027	161.35	71.93	100.00	78.68	80.18	131.75	122.16	100.00	115.80	124.54	101.21	3.47

	**P-values**	**FDR**	**t0**	**CFD_t1**	**CRD_t1**	**MYC_t1**	**NFB_t1**	**NFB+MYC_t1**	**CFD_t2**	**CRD_t2**	**MYC_t2**	**NFB_t2**	**NFB+MYC_t2**	**LDA score**
Luteimonas	0.00146	0.00479	28.46	74.33	100.00	40.70	61.80	36.15	151.40	100.00	73.31	64.74	62.17	2.88
Luteolibacter	0.00907	0.01899	36.75	78.53	100.00	78.33	81.55	64.93	119.50	100.00	72.50	76.69	48.52	4.23
Lysinimonas	0.00915	0.01900	54.49	86.28	100.00	99.21	75.61	87.15	147.76	100.00	106.85	81.66	108.60	2.71
Lysobacter	0.00463	0.01133	77.25	108.82	100.00	124.87	107.29	105.70	116.43	100.00	81.36	74.20	99.11	3.9
Marisediminicola	0.01599	0.03026	80.63	56.83	100.00	130.20	110.57	171.46	98.77	100.00	117.59	72.77	146.36	2.18
Marmoricola	0.00516	0.01234	136.58	109.05	100.00	113.15	111.21	116.51	91.52	100.00	79.95	77.44	93.13	4.1
Massilia	0.01029	0.02095	105.31	105.14	100.00	139.74	133.55	159.33	96.04	100.00	87.28	67.44	120.86	4.26
Meiothermus	0.01882	0.03391	0.76	257.34	100.00	72.70	237.91	114.24	83.92	100.00	120.16	202.97	87.72	2.62
Mesorhizobium	0.00033	0.00168	34.77	65.79	100.00	75.75	90.96	78.33	79.24	100.00	75.81	75.84	79.08	4.14
Methylobacillus	0.00000	0.00000	5.38	93.05	100.00	103.05	28.30	41.26	342.82	100.00	170.96	79.15	180.94	3.42
Methyloceanibacter	0.02824	0.04566	147.54	101.20	100.00	88.94	87.24	69.99	84.87	100.00	120.88	113.23	77.73	2.21
Methylocella	0.00140	0.00468	30.06	58.14	100.00	87.85	116.33	84.68	85.72	100.00	84.49	85.77	116.62	2.38
Methylopila	0.01773	0.03250	23.30	30.27	100.00	54.03	46.79	34.44	18.90	100.00	54.92	24.46	61.84	2.66
Methylorosula	0.00343	0.00929	77.34	103.39	100.00	139.35	108.02	130.09	88.20	100.00	83.42	84.67	84.97	2.58
Methylotenera	0.00001	0.00008	28.88	96.79	100.00	110.35	84.59	77.59	276.32	100.00	132.97	91.93	305.08	3.82
Microbacterium	0.00156	0.00502	63.53	117.07	100.00	99.84	81.16	68.36	165.33	100.00	89.40	61.77	101.83	3.96
Micropepsis	0.00002	0.00024	0.44	45.32	100.00	36.72	88.06	42.96	28.29	100.00	39.18	25.48	12.13	2.86
Microvirga	0.00292	0.00823	102.66	115.92	100.00	152.21	146.16	151.77	78.26	100.00	78.81	83.85	90.45	4.58
Mitsuaria	0.00000	0.00002	2.22	54.64	100.00	35.42	58.93	28.41	458.67	100.00	574.76	234.44	125.09	4.11
Mucilaginibacter	0.00012	0.00075	128.95	84.91	100.00	106.21	111.10	122.41	67.69	100.00	42.64	64.10	26.47	2.77
Mycobacterium	0.00000	0.00004	104.05	84.17	100.00	90.21	93.05	92.86	85.18	100.00	78.58	85.94	78.60	3.78
Nakamurella	0.02125	0.03693	108.22	87.64	100.00	118.56	134.29	171.12	115.27	100.00	114.29	109.63	128.42	2.72

Nannocystis	0.01458	0.02833	61.56	79.39	100.00	132.68	166.94	105.79	81.28	100.00	63.21	83.42	60.39	2.55
Niastella	0.00004	0.00035	50.61	95.04	100.00	86.82	115.95	64.68	105.49	100.00	100.19	96.98	84.42	3.24
Nibribacter	0.00774	0.01672	120.70	147.16	100.00	223.21	184.33	182.70	157.48	100.00	91.19	67.05	110.41	2.72

	P-values	FDR	t0	CFD_t1	CRD_t1	MYC_t1	NFB_t1	NFB+MYC_t1	CFD_t2	CRD_t2	MYC_t2	NFB_t2	NFB+MYC_t2	LDA score
Nitratireductor	0.01276	0.02537	56.07	104.51	100.00	138.26	101.05	85.82	127.48	100.00	130.05	132.33	153.52	2.48
Nitrosomonas	0.00033	0.00168	23.25	124.00	100.00	132.04	165.36	150.81	74.88	100.00	121.19	102.89	95.57	2.94
Nitrosospira	0.03976	0.06052	51.71	89.21	100.00	104.22	156.70	143.81	87.85	100.00	132.13	113.75	93.61	3.09
Nocardioides	0.00014	0.00084	109.17	94.91	100.00	97.53	101.81	90.18	133.76	100.00	102.76	103.68	121.27	4.31
Nodosilinea PCC 7104	0.00011	0.00069	1.27	27.62	100.00	45.33	53.11	142.19	158.41	100.00	175.05	143.44	248.30	3.85
Nonomuraea	0.00531	0.01258	124.49	78.96	100.00	104.45	94.41	148.25	119.06	100.00	130.27	134.30	110.33	3.13
Nordella	0.00009	0.00063	88.84	115.48	100.00	88.58	98.28	77.71	81.70	100.00	95.49	108.24	82.93	3.96
Nostoc PCC 7524	0.01177	0.02368	42.21	94.18	100.00	67.56	56.46	84.67	145.11	100.00	320.22	154.37	220.48	2.51
Noviherbaspirillum	0.00380	0.00997	83.61	117.01	100.00	153.12	138.77	139.54	103.51	100.00	97.33	79.30	126.22	3.97
Novosphingobium	0.00157	0.00502	82.09	69.64	100.00	77.13	69.08	59.67	129.29	100.00	72.61	85.04	124.33	3.14
Ohtaekwangia	0.02306	0.03899	40.51	85.30	100.00	84.47	73.60	71.64	98.05	100.00	78.49	76.69	84.69	3.68
Oligoflexus	0.03917	0.05997	86.88	97.91	100.00	164.55	124.22	159.70	102.53	100.00	101.15	103.54	134.63	3.05
Oscillatoria SAG 1459 8	0.00208	0.00634	2.56	53.90	100.00	91.79	122.72	74.28	111.09	100.00	176.60	202.91	248.52	2.65
Oscillochloris	0.01618	0.03042	24.99	81.29	100.00	88.09	59.65	78.90	144.26	100.00	94.04	107.89	112.49	2.35
Paenarthrobacter	0.00150	0.00491	132.34	170.53	100.00	134.26	115.60	117.67	203.61	100.00	152.01	118.41	199.72	3.75
Paenisporosarcina	0.01173	0.02368	129.89	107.55	100.00	99.88	89.99	138.05	68.75	100.00	75.72	88.84	92.10	3.5
Panacagrimonas	0.00363	0.00975	21.53	101.32	100.00	129.13	95.29	68.88	164.54	100.00	149.42	78.67	110.21	2.8
Parasegetibacter	0.00005	0.00036	46.59	94.90	100.00	135.00	106.91	143.75	113.69	100.00	118.57	88.79	153.50	3.27
Parviterribacter	0.00672	0.01496	103.87	96.87	100.00	106.83	101.11	102.46	107.97	100.00	89.91	87.59	94.42	3.06
Paucibacter	0.00958	0.01982	49.43	139.78	100.00	128.94	102.81	117.20	97.74	100.00	85.07	110.51	110.19	2.71
Pedinomonas minor	0.02292	0.03888	73.77	98.41	100.00	186.87	181.53	189.17	111.97	100.00	113.93	157.40	141.15	2.52
Pedobacter	0.00003	0.00027	78.08	114.27	100.00	135.10	124.19	147.23	101.02	100.00	91.55	87.86	112.84	3.62
Pedococcus Phycicoccus	0.00101	0.00368	123.40	109.53	100.00	99.92	103.24	85.69	162.80	100.00	109.36	125.83	152.91	2.93

Pedomicrobium	0.00016	0.00090	134.73	96.26	100.00	90.72	98.24	95.56	86.15	100.00	102.56	110.27	78.61	3.78
Pelomonas	0.00023	0.00125	14.15	62.72	100.00	75.89	53.81	61.63	215.85	100.00	87.79	101.50	174.70	2.86
	**P-values**	**FDR**	**t0**	**CFD_t1**	**CRD_t1**	**MYC_t1**	**NFB_t1**	**NFB+MYC_t1**	**CFD_t2**	**CRD_t2**	**MYC_t2**	**NFB_t2**	**NFB+MYC_t2**	**LDA score**
Pelosinus	0.00030	0.00155	197.56	99.96	100.00	92.51	89.74	144.30	85.91	100.00	82.75	104.93	74.56	2.89
Peredibacter	0.00041	0.00193	60.59	117.28	100.00	125.12	131.72	124.28	105.57	100.00	91.41	78.01	90.45	3.04
Phenylobacterium	0.00000	0.00001	84.95	75.81	100.00	93.64	83.44	89.60	100.95	100.00	89.52	80.78	114.49	3.08
Pirellula	0.00055	0.00235	87.64	117.64	100.00	88.22	91.16	63.43	94.10	100.00	97.31	97.75	80.03	4.57
Piscinibacter	0.00093	0.00340	55.14	112.29	100.00	109.43	85.05	108.75	140.31	100.00	107.09	116.14	145.82	2.75
Planctomicrobium	0.00141	0.00468	65.40	122.28	100.00	84.68	75.41	52.38	123.23	100.00	93.16	90.94	71.46	2.9
Planctomycetaceae bacterium (uncultured)	0.00291	0.00823	67.72	108.92	100.00	98.12	95.17	57.91	98.81	100.00	209.03	181.13	95.23	2.05
Planctomycetales bacterium (uncultured)	0.00001	0.00015	54.84	82.79	100.00	84.01	85.91	49.26	84.43	100.00	85.51	99.10	68.67	3.11
Planctomycete (uncultured)	0.00005	0.00041	71.15	132.70	100.00	128.00	126.96	105.40	91.75	100.00	80.96	78.52	76.36	4.31
Planctomycete WY108	0.00543	0.01270	48.95	174.69	100.00	214.36	144.17	131.94	120.25	100.00	93.47	107.28	118.98	2.16
Planctopirus	0.00001	0.00013	17.93	77.78	100.00	58.17	104.83	63.50	63.60	100.00	54.61	52.90	53.24	3.4
Planifilum	0.02763	0.04495	137.18	49.23	100.00	79.06	68.51	113.09	82.14	100.00	111.93	84.94	102.65	2.96
Planoglabratella opercularis	0.00541	0.01270	20.28	105.39	100.00	136.72	160.05	128.03	116.56	100.00	124.64	138.84	149.62	2.56
Polyangiaceae bacterium (uncultured)	0.00321	0.00890	55.47	196.31	100.00	151.59	101.37	95.39	65.94	100.00	63.27	67.70	36.74	2.29
Polyangium brachysporum group	0.00000	0.00000	39.47	116.57	100.00	135.21	101.46	69.86	86.01	100.00	46.72	63.76	62.93	3.66
Polycyclovorans	0.02839	0.04576	55.46	78.16	100.00	101.07	74.91	100.27	119.18	100.00	127.02	73.60	40.06	3.39
Pontibacter	0.00072	0.00276	66.68	117.17	100.00	155.30	142.35	172.54	117.87	100.00	101.27	94.33	114.08	3.72
Promicromonospora	0.01565	0.02987	123.34	64.10	100.00	48.00	63.53	76.08	86.31	100.00	57.23	44.36	47.32	3.19
Prosthecomicrobium	0.00299	0.00839	61.96	152.76	100.00	154.58	150.54	160.92	114.03	100.00	60.44	74.82	99.33	2.46
Pseudarthrobacter	0.02186	0.03769	102.57	131.13	100.00	172.71	129.13	142.73	139.74	100.00	130.33	105.27	193.50	3.4
Pseudenhygromyxa	0.03489	0.05439	31.52	77.58	100.00	104.54	105.64	131.98	49.90	100.00	77.30	88.66	56.81	2.36
Pseudoduganella	0.00011	0.00069	17.40	121.29	100.00	200.23	88.62	49.94	124.62	100.00	119.58	78.71	56.80	3.5
Pseudoflavitalea	0.00776	0.01672	59.27	89.15	100.00	69.24	95.00	119.87	32.42	100.00	59.46	70.79	43.95	3.16
Pseudolabrys	0.00116	0.00412	41.66	61.28	100.00	44.77	79.27	45.11	90.60	100.00	83.44	78.08	71.18	3.96

	P-values	FDR	t0	CFD_t1	CRD_t1	MYC_t1	NFB_t1	NFB+MYC_t1	CFD_t2	CRD_t2	MYC_t2	NFB_t2	NFB+MYC_t2	LDA score
Pseudomuriella schumacherensis	0.00011	0.00069	9.95	109.78	100.00	116.64	110.14	105.29	60.84	100.00	117.72	189.98	176.49	3.45
Pseudonocardia	0.00687	0.01510	105.67	90.27	100.00	106.52	102.00	131.41	109.58	100.00	104.30	130.96	104.85	3.12
Pseudorhodoplanes	0.00166	0.00519	63.49	91.61	100.00	83.16	115.08	71.04	66.50	100.00	83.95	89.45	72.74	3.02
Pseudoxanthomonas	0.00079	0.00298	29.63	61.20	100.00	56.24	62.95	48.53	132.30	100.00	96.14	59.35	78.10	3.92
Qipengyuania	0.00000	0.00001	39.43	116.70	100.00	131.69	98.66	92.84	155.03	100.00	104.04	95.89	155.75	3.74
Ramlibacter	0.00027	0.00143	67.60	115.18	100.00	149.27	140.85	138.50	98.05	100.00	81.35	71.17	89.91	4.02
Reyranella	0.00109	0.00391	57.18	83.95	100.00	73.19	80.00	67.50	77.77	100.00	94.70	94.04	70.41	3.52
Rhizobacter	0.00054	0.00233	51.84	89.50	100.00	99.14	93.45	100.15	112.80	100.00	91.61	86.59	108.99	3.89
Rhodanobacter	0.00000	0.00000	0.33	31.79	100.00	30.97	70.29	43.82	33.48	100.00	32.63	29.11	23.08	3.55
Rhodobacter	0.00003	0.00029	30.18	66.39	100.00	123.97	119.68	117.41	167.67	100.00	113.28	116.05	182.43	3.37
Rhodococcus	0.02581	0.04266	242.05	156.02	100.00	108.03	138.33	131.74	97.22	100.00	56.05	65.49	89.39	2.88
Rhodocytophaga	0.01730	0.03195	57.71	112.16	100.00	124.53	121.04	140.31	101.90	100.00	120.75	92.20	117.51	3.17
Rhodoferax	0.00003	0.00029	29.85	59.06	100.00	62.05	102.08	86.94	92.10	100.00	81.06	66.96	89.19	2.85
Rhodopirellula	0.00523	0.01246	61.47	118.88	100.00	123.60	121.51	107.31	87.70	100.00	73.72	76.72	77.92	3.51
Rhodoplanes	0.00246	0.00714	85.95	107.72	100.00	81.97	93.18	70.72	94.72	100.00	110.25	116.78	84.46	3.66
Rhodopseudomonas	0.00001	0.00011	5.85	53.43	100.00	61.68	110.54	74.57	51.00	100.00	40.05	47.31	55.38	2.85
Roseisolibacter	0.00233	0.00684	58.98	72.58	100.00	102.52	87.00	111.67	93.56	100.00	85.22	62.68	90.02	2.67
Roseomonas	0.00651	0.01456	74.48	108.64	100.00	146.11	146.85	163.02	90.71	100.00	88.52	84.38	99.84	3.07
Rubellimicrobium	0.00020	0.00112	65.10	119.39	100.00	171.86	166.67	183.54	113.82	100.00	98.95	86.23	124.64	3.96
Rubrobacter	0.01717	0.03182	102.19	94.74	100.00	117.61	117.96	128.48	92.27	100.00	99.10	106.35	107.65	4.48
Rubrobacterales bacterium (uncultured)	0.00000	0.00000	127.08	89.23	100.00	82.87	85.86	79.50	121.13	100.00	99.84	114.48	102.34	4.35
Rubrobacteria bacterium (uncultured)	0.00000	0.00000	109.47	81.54	100.00	87.00	84.05	87.79	120.24	100.00	107.32	116.65	109.18	3.86
Ruminiclostridium	0.00036	0.00177	215.69	79.90	100.00	119.46	107.51	182.77	90.57	100.00	103.58	123.13	99.04	3.1
Saccharothrix	0.01654	0.03088	77.43	135.44	100.00	106.53	96.15	137.96	151.24	100.00	106.66	92.50	127.19	3
Salinispora	0.00156	0.00502	149.68	90.55	100.00	88.71	107.37	100.38	84.55	100.00	110.66	165.30	90.13	2.55
Sandaracinus	0.01947	0.03461	79.76	88.35	100.00	108.21	87.37	94.59	75.57	100.00	79.73	83.18	65.55	2.87

	P-values	FDR	t0	CFD_t1	CRD_t1	MYC_t1	NFB_t1	NFB+MYC_t1	CFD_t2	CRD_t2	MYC_t2	NFB_t2	NFB+MYC_t2	LDA score
Schlesneria	0.00001	0.00013	1.85	64.23	100.00	74.15	125.00	35.60	136.87	100.00	67.06	59.16	78.61	2.53
Segetibacter	0.00109	0.00391	131.43	164.49	100.00	160.08	164.99	133.63	126.07	100.00	97.40	109.97	144.88	2.76
Shimazuella	0.00369	0.00983	119.84	70.97	100.00	92.89	94.47	148.58	117.43	100.00	127.10	109.53	106.42	3.02
Shinella	0.00006	0.00047	10.00	63.21	100.00	47.57	41.76	32.00	180.22	100.00	139.36	98.72	88.63	3.67
Sideroxydans	0.00040	0.00187	1468.74	60.33	100.00	130.88	135.90	180.84	117.40	100.00	254.30	596.40	90.62	3.21
Simplicispira	0.00000	0.00001	2.21	38.09	100.00	33.72	76.34	42.26	34.95	100.00	40.10	21.02	17.23	2.86
Skermanella	0.02010	0.03512	97.66	91.42	100.00	124.06	146.40	189.17	101.19	100.00	103.40	121.28	126.36	4.42
Solibacillus	0.02247	0.03851	132.91	63.36	100.00	85.98	79.65	119.32	103.37	100.00	87.01	60.99	119.22	2.25
Solimonas	0.00006	0.00042	15.16	53.45	100.00	58.65	38.99	36.00	54.38	100.00	30.29	28.82	19.25	2.76
Solirubrobacter	0.00002	0.00020	117.00	90.83	100.00	98.51	97.28	101.39	106.98	100.00	99.97	101.39	116.94	3.98
Sorangium	0.00128	0.00440	111.76	54.97	100.00	66.03	124.28	53.62	74.59	100.00	59.82	43.68	60.98	3.1
Sphingoaurantiacus	0.00002	0.00024	75.77	102.00	100.00	140.95	123.50	148.19	109.97	100.00	79.63	85.73	139.31	3
Sphingobium	0.00000	0.00004	6.50	85.28	100.00	126.43	88.32	62.07	198.67	100.00	104.33	69.07	112.98	4.63
Sphingomonas	0.00001	0.00014	86.61	98.85	100.00	113.30	107.96	101.69	103.29	100.00	81.86	82.08	103.17	4.59
Sphingopyxis	0.00000	0.00000	13.19	52.79	100.00	54.95	54.82	33.90	226.83	100.00	101.10	88.39	101.31	3.72
Spirochaeta	0.00085	0.00314	1188.97	142.63	100.00	332.97	188.75	104.31	104.63	100.00	85.17	98.04	168.04	2.91
Sporosarcina	0.00060	0.00244	133.18	96.65	100.00	118.71	98.29	147.67	93.88	100.00	102.54	115.32	131.64	3.08
Stenotrophomonas	0.02444	0.04093	199.02	134.96	100.00	23.50	103.31	19.36	228.62	100.00	68.97	35.64	15.56	2.78
Steroidobacter	0.00120	0.00425	111.07	86.77	100.00	88.55	96.64	110.41	84.30	100.00	101.10	109.06	90.59	3.82
Streptomyces	0.00042	0.00194	71.96	98.50	100.00	76.03	79.34	81.44	114.70	100.00	83.80	95.92	101.38	4.12
Streptosporangium	0.02289	0.03888	89.70	68.18	100.00	64.35	109.29	122.91	54.42	100.00	70.47	72.77	82.54	2.49
Symbiobacterium	0.00616	0.01394	138.01	74.49	100.00	84.27	79.72	116.24	114.38	100.00	134.28	124.71	166.57	2.77
Synechococcus IR11	0.00009	0.00063	1.17	24.40	100.00	81.42	90.87	110.63	33.37	100.00	35.81	101.17	116.51	3.41
Syntrophobacter	0.02445	0.04093	408.53	165.94	100.00	141.38	126.33	190.93	98.82	100.00	94.84	114.59	109.18	2.77
Tahibacter	0.01475	0.02857	40.46	130.58	100.00	79.87	276.88	150.40	92.45	100.00	48.28	35.71	44.22	3.16

	P-values	FDR	t0	CFD_t1	CRD_t1	MYC_t1	NFB_t1	NFB+MYC_t1	CFD_t2	CRD_t2	MYC_t2	NFB_t2	NFB+MYC_t2	LDA score
Telmatocola	0.04084	0.06197	102.43	160.13	100.00	198.50	127.09	177.40	103.11	100.00	67.28	83.84	78.11	2.39
Tepidisphaera	0.01603	0.03026	66.91	132.08	100.00	155.33	143.77	107.34	93.59	100.00	89.10	68.97	71.14	3.21
Terribacillus	0.00621	0.01395	115.45	71.97	100.00	93.63	75.42	129.49	67.15	100.00	88.64	109.28	319.05	3.01
Terrimicrobium	0.00022	0.00122	31.54	61.12	100.00	61.30	88.98	89.05	129.92	100.00	97.33	95.52	73.67	2.82
Terrimonas	0.00018	0.00105	61.82	108.01	100.00	97.60	109.12	78.26	80.15	100.00	79.62	80.68	76.54	3.87
Thermoactinomyces	0.03773	0.05811	152.77	60.24	100.00	84.02	65.36	94.53	70.04	100.00	103.71	80.47	65.09	2.72
Thermoflavimicrobium	0.00439	0.01097	123.06	47.71	100.00	80.35	61.86	142.06	87.39	100.00	105.50	111.77	93.36	2.73
Truepera	0.00000	0.00000	59.33	149.29	100.00	156.25	98.88	142.63	72.80	100.00	85.78	57.10	100.76	3.5
Tumebacillus	0.00057	0.00238	111.77	50.94	100.00	60.02	68.78	112.49	125.25	100.00	160.19	147.87	120.88	3.76
Tychonema CCAP 1459 11B	0.01920	0.03424	30.18	110.87	100.00	70.93	148.18	187.18	147.59	100.00	265.50	518.14	341.60	4.18
Vampirovibrio	0.00033	0.00167	6.67	138.57	100.00	103.26	102.34	58.79	93.57	100.00	81.25	98.34	96.78	2.42
Variovorax	0.00056	0.00237	60.99	90.24	100.00	68.56	75.48	78.65	140.86	100.00	104.24	96.06	91.64	3.69
Verrucomicrobia bacterium (uncultured)	0.00010	0.00067	37.21	128.97	100.00	129.69	99.99	70.03	113.64	100.00	76.09	75.61	86.16	2.63
Verrucomicrobiales bacterium (uncultured)	0.00006	0.00042	33.78	92.42	100.00	95.41	105.24	84.33	160.08	100.00	117.16	132.74	87.96	2.54
Virgibacillus	0.00003	0.00027	23.82	234.26	100.00	400.03	193.54	41.86	119.56	100.00	123.75	184.54	107.02	3.39
Vischeria sp CAUP Q 202	0.00840	0.01788	22.96	73.69	100.00	92.99	53.07	157.81	127.89	100.00	150.93	68.68	184.88	2.54
Yonghaparkia	0.00000	0.00000	12.03	105.13	100.00	134.70	88.89	58.25	159.11	100.00	110.05	84.52	154.23	3.61
Zavarzinella	0.00465	0.01133	80.51	112.21	100.00	98.99	88.18	62.54	81.85	100.00	94.48	93.31	88.24	3.06

Table S5. Signature associated to each treatment. Data grouped by treatment. Percentual standardization of values vs. t0. Negative differences greater than 10% are highlighted in red, while positive differences greater than 10% are highlighted in green.

	P-values	FDR	t0	CFD	CRD	MYC	NFB	NFB+MYC	LDA score	Notes
Acidibacter	0.00135	0.00721	100.00	157.79	188.63	152.66	186.49	161.97	3.74	
Acidimicrobiia bacterium (uncultured)	0.00034	0.00310	100.00	53.83	55.46	49.88	52.60	53.77	4.02	
Acidobacteriales bacterium (uncultured)	0.00814	0.02477	100.00	70.29	75.25	77.50	85.03	65.31	4.17	
Acidothermus	0.00004	0.00110	100.00	12.70	12.09	8.49	11.93	12.94	2.83	
Acidovorax	0.00017	0.00217	100.00	525.75	569.95	610.70	487.47	290.51	3.18	
Actinocorallia	0.00037	0.00321	100.00	53.11	76.99	58.99	74.69	35.68	2.99	
Actinomadura	0.04147	0.08374	100.00	71.08	71.62	72.17	78.15	78.65	2.62	
Adhaeribacter	0.02146	0.05156	100.00	114.64	107.25	138.19	128.26	156.92	3.62	
Aeromicrobium	0.00062	0.00437	100.00	267.36	232.89	230.42	196.70	202.73	4.42	
Allorhizobium, Neorhizobium, Pararhizobium, Rhizobium	0.00061	0.00437	100.00	211.77	203.97	200.70	208.29	217.22	4.34	
Alsobacter	0.00583	0.01964	100.00	110.05	123.69	149.79	140.99	168.51	2.86	
Altererythrobacter	0.00026	0.00280	100.00	220.07	272.27	190.01	198.67	191.16	3.51	
Amaricoccus	0.00411	0.01502	100.00	117.27	120.17	128.48	141.66	186.01	3.27	
Aminobacter	0.00000	0.00009	100.00	193.20	214.51	532.21	243.71	468.37	3.27	
Ammoniphilus	0.00565	0.01921	100.00	76.02	94.24	71.26	71.82	103.99	2.77	
Amycolatopsis	0.00240	0.01076	100.00	1440.22	508.22	700.89	686.08	822.61	3.37	
Anaerobacterium	0.01125	0.03106	100.00	27.98	32.20	34.26	28.67	35.95	2.71	
Anaerolinea	0.00972	0.02788	100.00	23.74	19.67	26.38	27.35	42.11	3.76	
Anaeromyxobacter	0.00150	0.00749	100.00	54.13	55.19	57.00	61.09	69.02	3.54	
Anaeromyxobacter dehalogenans	0.01014	0.02859	100.00	117.99	211.89	243.13	161.55	251.13	1.93	
Aquicella	0.00069	0.00453	100.00	400.24	342.61	298.48	324.07	222.90	2.59	
Aquisphaera	0.00816	0.02477	100.00	48.55	49.54	52.36	51.23	51.84	2.73	
	**P-values**	**FDR**	**t0**	**CFD**	**CRD**	**MYC**	**NFB**	**NFB+MYC**	**LDA score**	**Notes**
Arenimonas	0.00036	0.00315	100.00	283.07	266.77	236.75	183.06	237.44	3.4	

Aridibacter	0.01146	0.03146	100.00	159.95	138.25	143.06	157.97	164.41	3.1	
Armatimonas	0.00325	0.01266	100.00	639.74	710.98	805.05	613.50	689.89	1.88	
Arthrobacter	0.00246	0.01081	100.00	118.28	98.92	131.74	114.02	161.97	5.2	
Asticcacaulis	0.00906	0.02657	100.00	293.05	701.84	328.65	283.00	213.54	3.01	
Aurantisolimonas	0.01847	0.04523	100.00	144.23	136.91	150.88	153.00	172.62	2.74	
Azohydromonas	0.00158	0.00781	100.00	239.37	234.91	228.99	217.31	253.70	3.29	
Azospira	0.00652	0.02145	100.00	4734.23	8060.76	5899.00	5515.57	2517.92	2.58	
Bacillus	0.03987	0.08147	100.00	106.08	108.48	97.97	90.69	144.34	4.92	
Bauldia	0.01432	0.03736	100.00	148.10	240.24	138.56	173.13	125.93	3.16	
Bdellovibrio	0.00114	0.00647	100.00	219.54	251.74	222.75	225.82	263.26	2.92	
Blastocatella	0.00211	0.00978	100.00	151.96	134.83	155.96	157.89	142.49	3.35	
Bosea	0.00039	0.00329	100.00	207.11	219.61	224.32	209.42	259.75	3.54	
Bradyrhizobium	0.00270	0.01138	100.00	141.82	158.18	142.53	162.90	140.39	3.84	
Brevifollis	0.00671	0.02164	100.00	31.03	42.10	28.48	19.07	45.67	2.25	
Burkholderia Caballeronia Paraburkholderia	0.02324	0.05508	100.00	62.61	72.49	45.58	43.79	65.25	3.34	
Burkholderiales bacterium Beta 02	0.00021	0.00248	100.00	549.87	379.90	316.74	300.81	302.25	2.23	
Caenimonas	0.00017	0.00217	100.00	187.18	172.24	182.56	177.82	216.67	4.01	
Caldicoprobacter	0.04294	0.08572	100.00	53.88	56.32	57.47	48.50	71.32	2.68	
Candidatus Accumulibacter	0.01659	0.04139	100.00	29.33	30.15	38.69	39.36	39.08	2.33	
Candidatus Chloroploca	0.01106	0.03082	100.00	184.76	160.09	214.97	219.70	236.49	2.77	
Candidatus Entotheonella	0.02561	0.05934	100.00	165.48	141.26	128.93	177.14	145.65	2.72	
Candidatus Saccharibacteria bacterium (uncultured)	0.04738	0.09386	100.00	218.05	130.50	172.89	138.10	192.85	3.23	

	P-values	FDR	t0	CFD	CRD	MYC	NFB	NFB+MYC	LDA score	Notes
Candidatus Udaeobacter	0.04927	0.09649	100.00	91.01	94.43	80.36	92.26	59.28	3.92	
Chelativorans	0.00000	0.00000	100.00	138.02	162.35	3466.73	189.88	3543.15	3.35	
Chlamydomonas reinhardtii	0.00279	0.01149	100.00	598.94	732.59	752.54	412.61	1200.63	2.13	

Chloroflexi bacterium (uncultured)	0.03765	0.07912	100.00	79.33	78.76	80.95	81.13	89.29	4.07	
Chloronema	0.00002	0.00075	100.00	279.35	453.96	702.83	585.09	531.78	3.01	
Chthonobacter	0.00855	0.02567	100.00	322.11	399.17	351.12	304.40	331.79	2.47	
Chthonomonas	0.00206	0.00964	100.00	119.67	132.17	129.92	134.99	148.75	2.87	
Clostridium sensu stricto 1	0.03619	0.07701	100.00	52.18	43.08	49.78	51.71	63.40	3.17	
Clostridium sensu stricto 8	0.02914	0.06518	100.00	56.47	57.97	58.51	59.83	76.53	2.86	
Cnuella	0.00138	0.00731	100.00	271.66	274.29	285.67	238.70	328.63	2.55	
Cohnella	0.00555	0.01919	100.00	52.83	64.47	62.80	66.18	81.87	3.46	
Comamonas	0.00277	0.01149	100.00	308.80	278.29	268.33	259.87	268.23	2.91	
Conexibacteraceae bacterium (uncultured)	0.00101	0.00613	100.00	57.24	50.43	54.68	61.73	40.75	2.42	
Croceicoccus	0.00671	0.02164	100.00	424.77	607.09	503.47	462.04	491.42	2.45	
Cupriavidus	0.00783	0.02447	100.00	190.08	314.05	200.41	210.44	304.29	3.35	
Dactylosporangium	0.03391	0.07334	100.00	61.18	60.75	54.62	66.19	83.34	2.61	
Deinococcus	0.00064	0.00437	100.00	49551.15	30841.57	15532.48	8153.31	17021.75	3.25	
delta proteobacterium WX81	0.01172	0.03202	100.00	57.47	48.26	55.91	55.36	48.06	2.25	
Deltaproteobacteria bacterium GWC2 55 46	0.00127	0.00702	100.00	27.19	21.23	25.94	29.61	45.63	2.47	
Desmochloris halophila	0.00010	0.00191	100.00	3385.70	2318.35	2323.58	2275.21	2554.72	3.21	
Desulfosporosinus	0.02631	0.06042	100.00	32.61	41.27	36.62	39.66	54.25	2.32	
Desulfuromonadaceae bacterium (uncultured)	0.04409	0.08767	100.00	44.53	43.90	42.18	40.94	42.73	2.09	
Desulfuromonadales bacterium (uncultured)	0.02821	0.06393	100.00	68.00	65.45	69.98	86.68	82.03	3.07	
Devosia	0.00000	0.00031	100.00	232.63	276.33	196.27	203.45	181.40	4.21	
	**P-values**	**FDR**	**t0**	**CFD**	**CRD**	**MYC**	**NFB**	**NFB+MYC**	**LDA score**	**Notes**
Dokdonella	0.00012	0.00194	#DIV/0!	#DIV/0!	#DIV/0!	#DIV/0!	#DIV/0!	#DIV/0!	2.53	
Dongia	0.03324	0.07219	100.00	73.75	79.49	68.61	76.81	66.62	3.54	
Dyadobacter	0.00071	0.00458	100.00	387.01	239.69	449.61	178.36	187.24	3.71	
Edaphochlorella mirabilis	0.03746	0.07912	100.00	601.26	712.79	319.24	298.75	311.17	2.71	
Ellin517	0.00016	0.00217	100.00	267.57	247.90	217.24	214.76	178.32	3.76	

	P-values	FDR	t0	CFD	CRD	MYC	NFB	NFB+MYC	LDA score	Notes
Ellin6055	0.01512	0.03866	100.00	171.55	149.33	152.25	147.82	157.14	3.91	
Ellin6067	0.01279	0.03459	100.00	191.63	199.13	179.75	166.28	160.42	3.69	
Ensifer	0.00103	0.00613	100.00	178.06	176.42	192.81	177.04	178.68	3.7	
Erythrobacter	0.00013	0.00206	100.00	3744.86	4146.48	6524.15	2921.39	3817.93	2.86	
Ettlia pseudoalveolaris	0.00226	0.01027	100.00	675.34	509.21	650.90	522.87	2149.46	3.06	
Euzebya	0.04205	0.08460	100.00	204.23	189.51	140.48	156.87	149.85	1.83	
Ferrovibrio	0.00285	0.01164	100.00	315.50	301.58	254.84	320.73	263.99	2.4	
Fictibacillus	0.00028	0.00290	100.00	46.94	66.47	47.99	52.57	65.18	3.69	
Fimbriiglobus	0.01002	0.02841	100.00	139.96	145.22	147.67	153.55	128.01	3.7	
Flavihumibacter	0.01403	0.03676	100.00	186.33	190.05	223.02	228.24	245.80	2.32	
Flavisolibacter	0.00964	0.02780	100.00	151.12	146.58	155.92	149.93	155.62	3.68	
Flavitalea	0.00005	0.00128	100.00	215.82	241.70	243.95	235.87	228.93	3.23	
Flavobacterium	0.00143	0.00737	100.00	348.73	310.85	245.91	148.59	227.92	3.27	
Gaiella	0.00123	0.00684	100.00	59.98	61.46	59.44	59.92	62.80	4.85	
Gemmatimonas	0.00022	0.00252	100.00	180.93	178.30	189.52	181.67	192.29	3.82	
Geoalkalibacter	0.00024	0.00268	100.00	11.34	10.17	18.51	19.90	22.58	2.98	
Green Bay ferromanganous micronodule bacterium MND4 (uncultured)	0.01637	0.04105	100.00	47.79	53.49	45.30	63.68	41.37	2.05	
Halobacillus	0.00341	0.01302	100.00	7498.85	3259.50	11216.43	14002.10	1630.88	3.22	
Herbinix	0.00653	0.02145	100.00	52.25	56.63	49.38	58.49	73.77	2.78	
	P-values	FDR	t0	CFD	CRD	MYC	NFB	NFB+MYC	LDA score	Notes
Herpetosiphon	0.01339	0.03583	100.00	183.72	159.48	174.43	147.30	160.51	3.72	
Hirschia	0.00047	0.00361	100.00	330.89	751.66	373.71	439.72	343.76	3.56	
Hungateiclostridium	0.01365	0.03634	100.00	46.35	54.88	57.36	55.69	100.63	1.9	
Hyaloperonospora arabidopsidis	0.00042	0.00339	100.00	823.22	653.95	611.61	638.67	531.57	2.24	
Hymenobacter	0.01716	0.04253	100.00	80.37	108.64	173.53	125.60	193.00	2.14	
Iamia	0.02128	0.05136	100.00	100.94	93.89	82.18	84.48	75.16	3.49	

	P-values	FDR	t0	CFD	CRD	MYC	NFB	NFB+MYC	LDA score	Notes
Ideonella	0.00064	0.00437	100.00	416.72	724.08	482.64	451.75	363.28	2.96	
Inquilinus	0.00465	0.01676	100.00	180.98	388.00	196.54	173.04	277.80	2.56	
Knoellia	0.02859	0.06422	100.00	86.09	78.51	82.94	85.75	98.04	4.05	
Kribbella	0.00474	0.01686	100.00	77.74	76.47	73.18	73.52	72.33	2.88	
Lacibacter	0.00473	0.01686	100.00	249.80	185.93	200.89	157.60	177.01	3.24	
Lautropia	0.01305	0.03508	100.00	191.16	169.79	183.23	215.49	223.99	2.46	
Lechevalieria	0.00109	0.00633	100.00	252.30	199.31	152.73	173.62	152.65	3.72	
Legionella	0.00111	0.00643	100.00	233.02	288.63	242.05	241.52	232.77	2.95	
Leptolyngbya EcFYyyy 00	0.00001	0.00055	100.00	2395.37	3967.85	7623.02	8405.69	6450.41	4.5	
Litorilinea	0.04870	0.09609	100.00	130.09	122.04	131.76	134.43	148.26	3.35	
Longimicrobium	0.00379	0.01424	100.00	272.24	394.41	503.33	389.70	394.80	2.9	
Luedemannella	0.00064	0.00437	100.00	44.62	49.27	45.34	47.61	59.33	3.41	
Luteimonas	0.00011	0.00194	100.00	334.49	310.36	172.05	195.75	147.24	2.76	
Luteolibacter	0.00033	0.00310	100.00	393.09	375.60	284.71	294.66	204.57	4.03	
Lysobacter	0.01745	0.04292	100.00	149.88	132.96	134.65	120.08	136.05	3.81	
Marmoricola	0.00385	0.01431	100.00	77.98	78.20	74.13	72.92	81.38	3.98	
Massilia	0.03121	0.06862	100.00	103.23	102.99	113.60	100.84	142.74	4	
Meiothermus	0.00309	0.01226	100.00	21118.16	11644.91	10979.05	25921.90	11949.56	2.5	
Mesorhizobium	0.00003	0.00095	100.00	203.47	281.17	212.76	234.98	221.27	4.13	
Methylobacillus	0.00008	0.00168	100.00	6340.32	2526.26	3806.42	1527.27	3273.55	3.18	
Methyloceanibacter	0.01474	0.03805	100.00	62.70	67.35	71.28	67.45	49.73	2.19	
Methylotenera	0.00002	0.00075	100.00	851.47	420.64	529.36	373.97	889.40	3.57	
Microbacterium	0.00265	0.01135	100.00	273.55	188.43	177.99	131.66	165.54	3.78	
Micropepsis	0.00018	0.00217	100.00	7972.94	21363.85	8075.31	12533.01	6083.81	2.84	
Microvirga	0.00245	0.01081	100.00	121.90	132.32	139.49	141.30	149.55	4.43	
Mitsuaria	0.00006	0.00145	100.00	8939.91	3937.08	11047.41	5269.27	2743.03	3.87	

	P-values	FDR	t0	CFD	CRD	MYC	NFB	NFB+MYC	LDA score	Notes
Mycobacterium	0.00128	0.00702	100.00	77.68	91.77	77.10	82.29	78.98	3.65	
Nakamurella	0.03916	0.08131	100.00	90.09	89.23	103.58	109.22	134.32	2.49	
Nannocystis	0.00698	0.02208	100.00	166.52	206.76	185.23	240.32	161.73	2.46	
Niastella	0.00012	0.00194	100.00	222.42	220.63	210.26	232.71	166.75	3.17	
Nitratireductor	0.00829	0.02501	100.00	179.34	156.75	208.81	179.52	180.26	2.35	
Nitrosomonas	0.00011	0.00194	100.00	417.19	416.04	524.84	562.42	516.46	2.82	
Nitrospira	0.02496	0.05835	100.00	109.65	118.90	128.44	132.40	111.57	4.01	
Nodosilinea PCC 7104	0.00001	0.00040	100.00	4131.96	5846.74	5253.08	4843.88	10355.46	3.82	
Nordella	0.01623	0.04090	100.00	125.66	130.54	122.17	135.69	105.32	3.65	
Noviherbaspirillum	0.00312	0.01227	100.00	151.22	138.30	166.95	145.23	182.53	3.9	
Ohtaekwangia	0.00348	0.01319	100.00	208.37	228.55	185.33	171.48	177.47	3.62	
Oscillatoria SAG 1459 8	0.00029	0.00290	100.00	2425.39	3188.72	4000.68	4903.90	4521.15	2.55	
Oscillochloris	0.00218	0.01001	100.00	381.63	351.88	316.76	283.12	328.62	2.27	
Paenarthrobacter	0.03870	0.08099	100.00	173.53	91.37	134.23	107.13	151.49	3.51	
Paenibacillus	0.03486	0.07476	100.00	82.59	88.89	82.36	85.02	113.37	4.14	
Paenisporosarcina	0.02545	0.05922	100.00	66.10	74.42	64.90	66.56	86.23	3.29	
Panacagrimonas	0.00140	0.00732	100.00	454.31	365.34	492.05	326.03	306.65	2.69	
Parasegetibacter	0.00001	0.00061	100.00	200.23	193.86	244.67	191.57	287.11	3.22	
	**P-values**	**FDR**	**t0**	**CFD**	**CRD**	**MYC**	**NFB**	**NFB+MYC**	**LDA score**	**Notes**
Paucibacter	0.00386	0.01431	100.00	249.77	212.05	222.36	226.54	240.75	2.62	
Pedococcus Phycicoccus	0.00988	0.02817	100.00	83.42	64.50	65.76	72.00	71.38	2.76	
Pelomonas	0.00078	0.00482	100.00	433.39	451.52	342.17	289.74	389.18	2.63	
Pelosinus	0.00331	0.01284	100.00	44.26	47.38	41.33	45.87	52.97	2.84	
Peredibacter	0.00887	0.02630	100.00	183.81	164.96	176.75	173.00	177.12	2.9	
Permianibacter	0.04910	0.09649	100.00	331.38	237.46	256.68	101.90	201.53	2.12	
Pirellula	0.00943	0.02735	100.00	134.74	128.91	121.57	122.25	93.70	4.33	
Piscinibacter	0.00304	0.01214	100.00	263.69	206.03	224.38	211.09	266.83	2.6	

Planctomicrobium	0.00693	0.02206	100.00	225.75	183.78	167.08	155.25	116.74	2.74	
Planctomycetales bacterium (uncultured)	0.00004	0.00110	100.00	164.97	197.15	168.15	183.36	117.69	3.01	
Planctomycete (uncultured)	0.00014	0.00210	100.00	199.46	186.03	183.93	180.11	162.46	4.19	
Planctomycete WY108	0.00177	0.00835	100.00	365.34	257.58	359.32	314.00	319.71	2.06	
Planctopirus	0.00001	0.00061	100.00	371.81	522.41	293.28	421.20	306.74	3.34	
Planoglabratella opercularis	0.00046	0.00360	100.00	451.26	410.79	532.97	622.62	561.38	2.44	
Polyangiaceae bacterium (uncultured)	0.01496	0.03843	100.00	409.92	443.47	365.97	330.59	215.81	2.05	
Polyangium brachysporum group	0.00007	0.00158	100.00	504.23	541.22	366.44	392.83	349.36	3.44	
Pontibacter	0.02821	0.06393	100.00	164.28	139.83	178.72	167.89	203.34	3.53	
prokaryote (uncultured)	0.00048	0.00361	100.00	41.79	42.79	46.33	49.23	51.45	3.31	
Promicromonospora	0.00609	0.02040	100.00	70.33	91.92	49.53	48.55	55.16	3.1	
Prosthecomicrobium	0.00149	0.00749	100.00	288.56	225.65	209.88	229.93	273.83	2.37	
proteobacterium (uncultured)	0.03905	0.08131	100.00	60.58	57.97	56.81	61.19	54.89	3.38	
Pseudarthrobacter	0.00689	0.02206	100.00	140.61	103.63	154.87	120.72	175.77	3.22	
Pseudoduganella	0.00000	0.00031	100.00	772.65	627.69	971.14	522.53	336.82	3.42	
Pseudoflavitalea	0.00752	0.02367	100.00	119.25	220.21	140.69	176.32	160.83	2.97	
Pseudomuriella schumacherensis	0.00002	0.00072	100.00	827.69	956.07	1116.56	1415.01	1329.46	3.36	

	P-values	FDR	t0	CFD	CRD	MYC	NFB	NFB+MYC	LDA score	Notes
Pseudorhodoplanes	0.00026	0.00280	100.00	133.98	171.75	144.39	173.80	123.59	2.96	
Pseudoxanthomonas	0.00026	0.00280	100.00	321.43	333.64	257.68	204.08	210.68	3.76	
Qipengyuania	0.00794	0.02467	100.00	238.59	185.24	220.71	181.15	208.75	3.51	
Ramlibacter	0.00015	0.00213	100.00	207.01	198.21	210.86	192.61	214.15	3.9	
Reyranella	0.00034	0.00310	100.00	144.20	178.47	151.68	155.56	123.12	3.5	
Rhizobacter	0.00006	0.00145	100.00	196.68	194.28	184.85	174.85	203.22	3.82	
Rhizorhapis	0.03420	0.07365	100.00	521.65	527.68	469.72	305.82	205.03	2.53	
Rhodanobacter	0.00032	0.00310	100.00	6182.61	19241.38	5791.37	11922.79	7624.38	3.34	

	P-values	FDR	t0	CFD	CRD	MYC	NFB	NFB+MYC	LDA score	Notes
Rhodobacter	0.00561	0.01921	100.00	212.11	226.58	265.30	268.97	305.63	3.19	
Rhodococcus	0.00295	0.01185	100.00	67.67	57.11	42.62	52.45	59.80	2.86	
Rhodocytophaga	0.00141	0.00732	100.00	196.57	184.19	226.34	194.81	236.19	3.15	
Rhodopirellula	0.00173	0.00822	100.00	191.05	188.92	178.43	181.38	171.11	3.4	
Rhodopseudomonas	0.00010	0.00190	100.00	767.28	1463.82	752.31	1233.40	974.78	2.73	
Roseomonas	0.00277	0.01149	100.00	149.99	152.08	171.76	170.26	194.25	2.97	
Rubellimicrobium	0.00012	0.00196	100.00	188.09	161.50	212.60	201.05	246.54	3.87	
Rubrobacterales bacterium (uncultured)	0.00040	0.00329	100.00	59.06	59.11	51.49	56.41	51.51	4.26	
Rubrobacteria bacterium (uncultured)	0.00927	0.02704	100.00	65.06	68.81	63.56	65.37	65.35	3.69	
Ruminiclostridium	0.00056	0.00413	100.00	35.56	41.99	46.63	48.08	61.00	3.09	
Salinispora	0.00566	0.01921	100.00	48.78	55.33	53.54	72.11	53.29	2.46	
Schlesneria	0.00287	0.01166	100.00	2607.86	3336.63	2323.99	3749.36	1463.13	2.27	
Shinella	0.00000	0.00027	100.00	696.22	710.83	535.63	417.05	346.96	3.5	
Sideroxydans	0.00429	0.01557	100.00	3.28	4.45	6.99	10.84	7.10	3.2	
Simplicispira	0.03159	0.06918	100.00	1024.65	2728.29	911.30	1824.57	1036.15	2.64	
Skermanella	0.01553	0.03951	100.00	96.47	100.28	113.41	134.47	158.86	4.21	
Solibacillus	0.00542	0.01887	100.00	54.20	67.00	57.46	47.88	79.91	2.16	
Sorangium	0.00668	0.02164	100.00	71.39	107.47	67.60	83.00	62.24	2.86	
Sphingoaurantiacus	0.01111	0.03082	100.00	130.06	123.06	135.28	130.42	177.29	2.87	
Sphingobium	0.00000	0.00031	100.00	2395.60	1644.64	1881.83	1283.87	1466.69	4.46	
Sphingopyxis	0.00006	0.00145	100.00	387.41	461.64	283.47	280.82	212.18	3.46	
Spirochaeta	0.00119	0.00674	100.00	10.41	8.42	16.90	12.07	11.47	2.91	
Sporichthya	0.03114	0.06862	100.00	260.90	247.75	232.39	214.39	240.03	2.34	
Sporosarcina	0.01384	0.03647	100.00	61.56	64.46	71.04	67.94	90.87	2.88	
Stenotrophomonas	0.00264	0.01135	100.00	112.49	59.49	30.80	38.21	10.21	2.62	

Streptomyces	0.00017	0.00217	100.00	149.50	140.16	112.48	122.92	128.24	4.02	
Symbiobacterium	0.02408	0.05658	100.00	44.57	51.60	50.51	48.05	67.71	2.68	
Synechococcus IR11	0.00001	0.00059	100.00	2708.54	9262.92	5150.79	8931.69	10541.35	3.37	
Syntrophobacter	0.00815	0.02477	100.00	32.74	24.82	28.88	29.88	37.11	2.77	
Tahibacter	0.00395	0.01455	100.00	391.75	372.76	220.43	431.14	296.03	2.91	
Tepidisphaera	0.01871	0.04559	100.00	188.74	170.94	199.77	173.80	148.66	3.09	
Terribacillus	0.00075	0.00474	100.00	55.94	80.20	72.73	72.98	173.80	2.81	
Terrimicrobium	0.00078	0.00482	100.00	316.61	327.65	266.27	302.58	265.75	2.65	
Terrimonas	0.00064	0.00437	100.00	189.43	208.22	181.83	191.00	160.76	3.71	
Tetradesmus obliquus	0.00069	0.00453	100.00	505.54	376.95	591.78	520.97	512.73	2.27	
Thermoactinomyces	0.02854	0.06422	100.00	34.30	53.56	48.70	38.15	44.49	2.68	
Thermoflavimicrobium	0.02207	0.05278	100.00	39.59	63.75	56.49	50.98	79.31	2.62	
Thermopolyspora	0.04261	0.08539	100.00	30.75	51.45	49.22	61.48	60.25	2.04	
Truepera	0.00069	0.00453	100.00	488.83	582.90	579.15	368.03	622.61	3.26	
Tumebacillus	0.04108	0.08329	100.00	40.15	58.60	48.75	51.27	67.08	3.67	
Vampirovibrio	0.00016	0.00217	100.00	2235.33	2028.60	1830.97	2024.79	1678.71	2.36	
Variovorax	0.00629	0.02092	100.00	196.60	169.04	149.25	145.50	144.25	3.52	
	**P-values**	**FDR**	**t0**	**CFD**	**CRD**	**MYC**	**NFB**	**NFB+MYC**	**LDA score**	**Notes**
Verrucomicrobia bacterium (uncultured)	0.03621	0.07701	100.00	261.89	212.32	225.91	193.30	161.26	2.44	
Verrucomicrobiales bacterium (uncultured)	0.00262	0.01135	100.00	492.90	370.47	409.81	451.05	320.49	2.35	
Vischeria sp CAUP Q 202	0.00036	0.00315	100.00	321.68	343.83	391.94	202.14	576.73	2.45	
Waddlia	0.03765	0.07912	100.00	90.60	77.89	67.47	55.91	57.57	2.48	
Yonghaparkia	0.00478	0.01689	100.00	584.56	508.41	631.45	447.87	385.15	3.33	

Table S6. Signature associated to each treatment. Data grouped by treatment. Percentual standardization of values vs. CFD. Negative differences greater than 10% are highlighted in red, while positive differences greater than 10% are highlighted in green.

	P-values	FDR	t0	CFD	CRD	MYC	NFB	NFB+MYC	LDA score
Acidibacter	0.00135	0.00721	63.38	100.00	119.55	96.75	118.19	102.65	3.74
Acidimicrobiia bacterium (uncultured)	0.00034	0.00310	185.77	100.00	103.04	92.66	97.71	99.89	4.02
Acidobacteriales bacterium (uncultured)	0.00814	0.02477	142.26	100.00	107.05	110.26	120.96	92.91	4.17
Acidothermus	0.00004	0.00110	787.27	100.00	95.15	66.85	93.90	101.91	2.83
Acidovorax	0.00017	0.00217	19.02	100.00	108.41	116.16	92.72	55.26	3.18
Actinocorallia	0.00037	0.00321	188.28	100.00	144.96	111.06	140.64	67.18	2.99
Actinomadura	0.04147	0.08374	140.69	100.00	100.76	101.53	109.95	110.64	2.62
Adhaeribacter	0.02146	0.05156	87.23	100.00	93.56	120.55	111.89	136.89	3.62
Aeromicrobium	0.00062	0.00437	37.40	100.00	87.11	86.18	73.57	75.83	4.42
Allorhizobium, Neorhizobium, Pararhizobium, Rhizobium	0.00061	0.00437	47.22	100.00	96.32	94.77	98.36	102.57	4.34
Alsobacter	0.00583	0.01964	90.86	100.00	112.38	136.11	128.11	153.11	2.86
Altererythrobacter	0.00026	0.00280	45.44	100.00	123.72	86.34	90.28	86.86	3.51
Amaricoccus	0.00411	0.01502	85.27	100.00	102.47	109.56	120.79	158.61	3.27
Aminobacter	0.00000	0.00009	51.76	100.00	111.03	275.47	126.14	242.42	3.27
Ammoniphilus	0.00565	0.01921	131.55	100.00	123.98	93.74	94.48	136.80	2.77
Amycolatopsis	0.00240	0.01076	6.94	100.00	35.29	48.67	47.64	57.12	3.37
Anaerobacterium	0.01125	0.03106	357.44	100.00	115.11	122.47	102.49	128.50	2.71
Anaerolinea	0.00972	0.02788	421.14	100.00	82.82	111.11	115.20	177.33	3.76
Anaeromyxobacter	0.00150	0.00749	184.74	100.00	101.96	105.31	112.85	127.50	3.54
Anaeromyxobacter dehalogenans	0.01014	0.02859	84.75	100.00	179.58	206.06	136.92	212.84	1.93
Aquicella	0.00069	0.00453	24.99	100.00	85.60	74.58	80.97	55.69	2.59
Aquisphaera	0.00816	0.02477	205.99	100.00	102.05	107.85	105.53	106.79	2.73
Arenimonas	0.00036	0.00315	35.33	100.00	94.24	83.63	64.67	83.88	3.4
Aridibacter	0.01146	0.03146	62.52	100.00	86.44	89.44	98.77	102.79	3.1
Armatimonas	0.00325	0.01266	15.63	100.00	111.14	125.84	95.90	107.84	1.88
Arthrobacter	0.00246	0.01081	84.54	100.00	83.63	111.38	96.40	136.94	5.2
Asticcacaulis	0.00906	0.02657	34.12	100.00	239.49	112.15	96.57	72.87	3.01
Aurantisolimonas	0.01847	0.04523	69.33	100.00	94.92	104.61	106.07	119.68	2.74
Azohydromonas	0.00158	0.00781	41.78	100.00	98.14	95.66	90.79	105.99	3.29
Azospira	0.00652	0.02145	2.11	100.00	170.27	124.60	116.50	53.19	2.58
Bacillus	0.03987	0.08147	94.27	100.00	102.26	92.35	85.49	136.06	4.92
Bauldia	0.01432	0.03736	67.52	100.00	162.22	93.56	116.90	85.03	3.16
Bdellovibrio	0.00114	0.00647	45.55	100.00	114.67	101.46	102.86	119.92	2.92
Blastocatella	0.00211	0.00978	65.81	100.00	88.73	102.63	103.90	93.77	3.35
Bosea	0.00039	0.00329	48.28	100.00	106.04	108.31	101.12	125.42	3.54

	P-values	FDR	t0	CFD	CRD	MYC	NFB	NFB+MYC	LDA score
Bradyrhizobium	0.00270	0.01138	70.51	100.00	111.54	100.50	114.86	98.99	3.84
Brevifollis	0.00671	0.02164	322.27	100.00	135.68	91.78	61.47	147.18	2.25
Burkholderia Caballeronia Paraburkholderia	0.02324	0.05508	159.71	100.00	115.77	72.79	69.95	104.22	3.34
Burkholderiales bacterium Beta 02	0.00021	0.00248	18.19	100.00	69.09	57.60	54.71	54.97	2.23
Caenimonas	0.00017	0.00217	53.42	100.00	92.02	97.53	95.00	115.75	4.01
Caldicoprobacter	0.04294	0.08572	185.60	100.00	104.54	106.67	90.02	132.37	2.68
Candidatus Accumulibacter	0.01659	0.04139	340.95	100.00	102.78	131.93	134.19	133.23	2.33
Candidatus Chloroploca	0.01106	0.03082	54.12	100.00	86.65	116.35	118.91	128.00	2.77
Candidatus Entotheonella	0.02561	0.05934	60.43	100.00	85.36	77.91	107.05	88.02	2.72
Candidatus Saccharibacteria bacterium (uncultured)	0.04738	0.09386	45.86	100.00	59.85	79.29	63.33	88.45	3.23
Candidatus Udaeobacter	0.04927	0.09649	109.87	100.00	103.75	88.30	101.37	65.14	3.92
Chelativorans	0.00000	0.00000	72.45	100.00	117.62	2511.76	137.57	2567.12	3.35
Chlamydomonas reinhardtii	0.00279	0.01149	16.70	100.00	122.31	125.64	68.89	200.46	2.13
Chloroflexi bacterium (uncultured)	0.03765	0.07912	126.06	100.00	99.29	102.05	102.27	112.57	4.07
Chloronema	0.00002	0.00075	35.80	100.00	162.51	251.60	209.45	190.37	3.01
Chthonobacter	0.00855	0.02567	31.04	100.00	123.92	109.01	94.50	103.00	2.47
Chthonomonas	0.00206	0.00964	83.56	100.00	110.44	108.56	112.80	124.29	2.87
Clostridium sensu stricto 1	0.03619	0.07701	191.66	100.00	82.56	95.42	99.11	121.51	3.17
Clostridium sensu stricto 8	0.02914	0.06518	177.07	100.00	102.64	103.60	105.93	135.52	2.86
Cnuella	0.00138	0.00731	36.81	100.00	100.97	105.16	87.87	120.97	2.55
Cohnella	0.00555	0.01919	189.29	100.00	122.03	118.87	125.27	154.96	3.46
Comamonas	0.00277	0.01149	32.38	100.00	90.12	86.90	84.16	86.86	2.91
Conexibacteraceae bacterium (uncultured)	0.00101	0.00613	174.70	100.00	88.10	95.53	107.84	71.19	2.42
Croceicoccus	0.00671	0.02164	23.54	100.00	142.92	118.53	108.77	115.69	2.45
Cupriavidus	0.00783	0.02447	52.61	100.00	165.22	105.43	110.71	160.08	3.35
Dactylosporangium	0.03391	0.07334	163.45	100.00	99.30	89.28	108.19	136.21	2.61
Deinococcus	0.00064	0.00437	0.20	100.00	62.24	31.35	16.45	34.35	3.25
delta proteobacterium WX81	0.01172	0.03202	174.00	100.00	83.98	97.29	96.33	83.62	2.25
Deltaproteobacteria bacterium GWC2 55 46	0.00127	0.00702	367.77	100.00	78.08	95.40	108.91	167.80	2.47
Desmochloris halophila	0.00010	0.00191	2.95	100.00	68.47	68.63	67.20	75.46	3.21
Desulfosporosinus	0.02631	0.06042	306.64	100.00	126.56	112.30	121.63	166.35	2.32
Desulfuromonadaceae bacterium (uncultured)	0.04409	0.08767	224.54	100.00	98.57	94.71	91.92	95.95	2.09
Desulfuromonadales bacterium (uncultured)	0.02821	0.06393	147.06	100.00	96.25	102.92	127.48	120.63	3.07
Devosia	0.00000	0.00031	42.99	100.00	118.78	84.37	87.46	77.98	4.21
Dokdonella	0.00012	0.00194	0.00	100.00	263.37	115.72	208.48	92.69	2.53
Dongia	0.03324	0.07219	135.59	100.00	107.78	93.03	104.15	90.32	3.54
Dyadobacter	0.00071	0.00458	25.84	100.00	61.93	116.18	46.09	48.38	3.71
Edaphochlorella mirabilis	0.03746	0.07912	16.63	100.00	118.55	53.10	49.69	51.75	2.71

	P-values	FDR	t0	CFD	CRD	MYC	NFB	NFB+MYC	LDA score
Ellin517	0.00016	0.00217	37.37	100.00	92.65	81.19	80.26	66.64	3.76
Ellin6055	0.01512	0.03866	58.29	100.00	87.05	88.75	86.17	91.60	3.91
Ellin6067	0.01279	0.03459	52.18	100.00	103.91	93.80	86.77	83.71	3.69
Ensifer	0.00103	0.00613	56.16	100.00	99.08	108.29	99.43	100.35	3.7
Erythrobacter	0.00013	0.00206	2.67	100.00	110.72	174.22	78.01	101.95	2.86
Ettlia pseudoalveolaris	0.00226	0.01027	14.81	100.00	75.40	96.38	77.42	318.28	3.06
Euzebya	0.04205	0.08460	48.97	100.00	92.79	68.79	76.81	73.37	1.83
Ferrovibrio	0.00285	0.01164	31.70	100.00	95.59	80.77	101.66	83.67	2.4
Fictibacillus	0.00028	0.00290	213.03	100.00	141.60	102.24	111.99	138.86	3.69
Fimbriiglobus	0.01002	0.02841	71.45	100.00	103.76	105.51	109.71	91.46	3.7
Flavihumibacter	0.01403	0.03676	53.67	100.00	101.99	119.69	122.49	131.92	2.32
Flavisolibacter	0.00964	0.02780	66.17	100.00	97.00	103.18	99.21	102.98	3.68
Flavitalea	0.00005	0.00128	46.33	100.00	111.99	113.03	109.29	106.08	3.23
Flavobacterium	0.00143	0.00737	28.68	100.00	89.14	70.52	42.61	65.36	3.27
Gaiella	0.00123	0.00684	166.72	100.00	102.47	99.09	99.89	104.69	4.85
Gemmatimonas	0.00022	0.00252	55.27	100.00	98.54	104.74	100.41	106.28	3.82
Geoalkalibacter	0.00024	0.00268	881.99	100.00	89.72	163.21	175.52	199.18	2.98
Green Bay ferromanganous micronodule bacterium MND4 (uncultured)	0.01637	0.04105	209.26	100.00	111.94	94.80	133.27	86.57	2.05
Halobacillus	0.00341	0.01302	1.33	100.00	43.47	149.58	186.72	21.75	3.22
Herbinix	0.00653	0.02145	191.37	100.00	108.38	94.50	111.93	141.17	2.78
Herpetosiphon	0.01339	0.03583	54.43	100.00	86.81	94.94	80.18	87.36	3.72
Hirschia	0.00047	0.00361	30.22	100.00	227.16	112.94	132.89	103.89	3.56
Hungateiclostridium	0.01365	0.03634	215.75	100.00	118.41	123.76	120.15	217.11	1.9
Hyaloperonospora arabidopsidis	0.00042	0.00339	12.15	100.00	79.44	74.29	77.58	64.57	2.24
Hymenobacter	0.01716	0.04253	124.42	100.00	135.18	215.91	156.27	240.13	2.14
Iamia	0.02128	0.05136	99.07	100.00	93.01	81.41	83.69	74.46	3.49
Ideonella	0.00064	0.00437	24.00	100.00	173.75	115.82	108.41	87.17	2.96
Inquilinus	0.00465	0.01676	55.26	100.00	214.39	108.60	95.62	153.50	2.56
Knoellia	0.02859	0.06422	116.16	100.00	91.20	96.35	99.61	113.89	4.05
Kribbella	0.00474	0.01686	128.64	100.00	98.37	94.13	94.57	93.05	2.88
Lacibacter	0.00473	0.01686	40.03	100.00	74.43	80.42	63.09	70.86	3.24
Lautropia	0.01305	0.03508	52.31	100.00	88.82	95.85	112.73	117.17	2.46
Lechevalieria	0.00109	0.00633	39.64	100.00	79.00	60.53	68.82	60.51	3.72
Legionella	0.00111	0.00643	42.91	100.00	123.86	103.88	103.65	99.89	2.95
Leptolyngbya EcFYyyy 00	0.00001	0.00055	4.17	100.00	165.65	318.24	350.91	269.29	4.5
Litorilinea	0.04870	0.09609	76.87	100.00	93.82	101.29	103.34	113.97	3.35
Longimicrobium	0.00379	0.01424	36.73	100.00	144.88	184.89	143.15	145.02	2.9
Luedemannella	0.00064	0.00437	224.11	100.00	110.42	101.60	106.70	132.96	3.41

	P-values	FDR	t0	CFD	CRD	MYC	NFB	NFB+MYC	LDA score
Luteimonas	0.00011	0.00194	29.90	100.00	92.79	51.44	58.52	44.02	2.76
Luteolibacter	0.00033	0.00310	25.44	100.00	95.55	72.43	74.96	52.04	4.03
Lysobacter	0.01745	0.04292	66.72	100.00	88.71	89.84	80.11	90.77	3.81
Marmoricola	0.00385	0.01431	128.24	100.00	100.27	95.06	93.50	104.36	3.98
Massilia	0.03121	0.06862	96.87	100.00	99.76	110.04	97.69	138.27	4
Meiothermus	0.00309	0.01226	0.47	100.00	55.14	51.99	122.75	56.58	2.5
Mesorhizobium	0.00003	0.00095	49.15	100.00	138.19	104.56	115.49	108.75	4.13
Methylobacillus	0.00008	0.00168	1.58	100.00	39.84	60.04	24.09	51.63	3.18
Methyloceanibacter	0.01474	0.03805	159.50	100.00	107.43	113.69	107.59	79.32	2.19
Methylotenera	0.00002	0.00075	11.74	100.00	49.40	62.17	43.92	104.45	3.57
Microbacterium	0.00265	0.01135	36.56	100.00	68.88	65.07	48.13	60.52	3.78
Micropepsis	0.00018	0.00217	1.25	100.00	267.95	101.28	157.19	76.31	2.84
Microvirga	0.00245	0.01081	82.03	100.00	108.55	114.43	115.91	122.68	4.43
Mitsuaria	0.00006	0.00145	1.12	100.00	44.04	123.57	58.94	30.68	3.87
Mycobacterium	0.00128	0.00702	128.73	100.00	118.13	99.25	105.92	101.67	3.65
Nakamurella	0.03916	0.08131	111.00	100.00	99.04	114.97	121.23	149.09	2.49
Nannocystis	0.00698	0.02208	60.05	100.00	124.16	111.23	144.32	97.12	2.46
Niastella	0.00012	0.00194	44.96	100.00	99.19	94.53	104.63	74.97	3.17
Nitratireductor	0.00829	0.02501	55.76	100.00	87.40	116.44	100.10	100.52	2.35
Nitrosomonas	0.00011	0.00194	23.97	100.00	99.72	125.80	134.81	123.80	2.82
Nitrospira	0.02496	0.05835	91.20	100.00	108.44	117.14	120.75	101.75	4.01
Nodosilinea PCC 7104	0.00001	0.00040	2.42	100.00	141.50	127.13	117.23	250.62	3.82
Nordella	0.01623	0.04090	79.58	100.00	103.88	97.22	107.98	83.81	3.65
Noviherbaspirillum	0.00312	0.01227	66.13	100.00	91.45	110.40	96.04	120.70	3.9
Ohtaekwangia	0.00348	0.01319	47.99	100.00	109.69	88.94	82.30	85.17	3.62
Oscillatoria SAG 1459 8	0.00029	0.00290	4.12	100.00	131.47	164.95	202.19	186.41	2.55
Oscillochloris	0.00218	0.01001	26.20	100.00	92.20	83.00	74.19	86.11	2.27
Paenarthrobacter	0.03870	0.08099	57.63	100.00	52.65	77.35	61.74	87.30	3.51
Paenibacillus	0.03486	0.07476	121.07	100.00	107.62	99.72	102.93	137.26	4.14
Paenisporosarcina	0.02545	0.05922	151.28	100.00	112.59	98.18	100.69	130.45	3.29
Panacagrimonas	0.00140	0.00732	22.01	100.00	80.42	108.31	71.76	67.50	2.69
Parasegetibacter	0.00001	0.00061	49.94	100.00	96.82	122.20	95.68	143.39	3.22
Paucibacter	0.00386	0.01431	40.04	100.00	84.90	89.02	90.70	96.39	2.62
Pedococcus Phycicoccus	0.00988	0.02817	119.88	100.00	77.32	78.83	86.32	85.57	2.76
Pelomonas	0.00078	0.00482	23.07	100.00	104.18	78.95	66.85	89.80	2.63
Pelosinus	0.00331	0.01284	225.94	100.00	107.05	93.38	103.63	119.69	2.84
Peredibacter	0.00887	0.02630	54.40	100.00	89.74	96.16	94.12	96.36	2.9
Permianibacter	0.04910	0.09649	30.18	100.00	71.66	77.46	30.75	60.82	2.12
Pirellula	0.00943	0.02735	74.22	100.00	95.68	90.23	90.73	69.54	4.33

	P-values	FDR	t0	CFD	CRD	MYC	NFB	NFB+MYC	LDA score
Piscinibacter	0.00304	0.01214	37.92	100.00	78.13	85.09	80.05	101.19	2.6
Planctomicrobium	0.00693	0.02206	44.30	100.00	81.41	74.01	68.77	51.71	2.74
Planctomycetales bacterium (uncultured)	0.00004	0.00110	60.62	100.00	119.51	101.93	111.15	71.34	3.01
Planctomycete (uncultured)	0.00014	0.00210	50.13	100.00	93.27	92.21	90.30	81.45	4.19
Planctomycete WY108	0.00177	0.00835	27.37	100.00	70.50	98.35	85.95	87.51	2.06
Planctopirus	0.00001	0.00061	26.90	100.00	140.51	78.88	113.29	82.50	3.34
Planoglabratella opercularis	0.00046	0.00360	22.16	100.00	91.03	118.11	137.97	124.40	2.44
Polyangiaceae bacterium (uncultured)	0.01496	0.03843	24.40	100.00	108.18	89.28	80.65	52.65	2.05
Polyangium brachysporum group	0.00007	0.00158	19.83	100.00	107.34	72.67	77.91	69.29	3.44
Pontibacter	0.02821	0.06393	60.87	100.00	85.11	108.79	102.20	123.78	3.53
prokaryote (uncultured)	0.00048	0.00361	239.28	100.00	102.40	110.85	117.81	123.11	3.31
Promicromonospora	0.00609	0.02040	142.18	100.00	130.69	70.42	69.03	78.43	3.1
Prosthecomicrobium	0.00149	0.00749	34.66	100.00	78.20	72.73	79.68	94.90	2.37
proteobacterium (uncultured)	0.03905	0.08131	165.06	100.00	95.69	93.77	101.00	90.60	3.38
Pseudarthrobacter	0.00689	0.02206	71.12	100.00	73.70	110.14	85.85	125.00	3.22
Pseudoduganella	0.00000	0.00031	12.94	100.00	81.24	125.69	67.63	43.59	3.42
Pseudoflavitalea	0.00752	0.02367	83.86	100.00	184.66	117.98	147.85	134.87	2.97
Pseudomuriella schumacherensis	0.00002	0.00072	12.08	100.00	115.51	134.90	170.96	160.62	3.36
Pseudorhodoplanes	0.00026	0.00280	74.64	100.00	128.18	107.77	129.72	92.24	2.96
Pseudoxanthomonas	0.00026	0.00280	31.11	100.00	103.80	80.17	63.49	65.55	3.76
Qipengyuania	0.00794	0.02467	41.91	100.00	77.64	92.51	75.93	87.49	3.51
Ramlibacter	0.00015	0.00213	48.31	100.00	95.75	101.86	93.04	103.45	3.9
Reyranella	0.00034	0.00310	69.35	100.00	123.76	105.19	107.88	85.38	3.5
Rhizobacter	0.00006	0.00145	50.84	100.00	98.78	93.98	88.90	103.32	3.82
Rhizorhapis	0.03420	0.07365	19.17	100.00	101.16	90.04	58.63	39.30	2.53
Rhodanobacter	0.00032	0.00310	1.62	100.00	311.22	93.67	192.84	123.32	3.34
Rhodobacter	0.00561	0.01921	47.15	100.00	106.82	125.08	126.81	144.09	3.19
Rhodococcus	0.00295	0.01185	147.77	100.00	84.40	62.99	77.51	88.37	2.86
Rhodocytophaga	0.00141	0.00732	50.87	100.00	93.70	115.14	99.10	120.15	3.15
Rhodopirellula	0.00173	0.00822	52.34	100.00	98.88	93.39	94.94	89.56	3.4
Rhodopseudomonas	0.00010	0.00190	13.03	100.00	190.78	98.05	160.75	127.04	2.73
Roseomonas	0.00277	0.01149	66.67	100.00	101.39	114.51	113.51	129.51	2.97
Rubellimicrobium	0.00012	0.00196	53.17	100.00	85.86	113.03	106.89	131.07	3.87
Rubrobacterales bacterium (uncultured)	0.00040	0.00329	169.33	100.00	100.10	87.18	95.53	87.23	4.26
Rubrobacteria bacterium (uncultured)	0.00927	0.02704	153.71	100.00	105.76	97.70	100.48	100.46	3.69
Ruminiclostridium	0.00056	0.00413	281.23	100.00	118.09	131.14	135.23	171.54	3.09
Salinispora	0.00566	0.01921	204.98	100.00	113.41	109.74	147.80	109.24	2.46
Schlesneria	0.00287	0.01166	3.83	100.00	127.95	89.11	143.77	56.10	2.27
Shinella	0.00000	0.00027	14.36	100.00	102.10	76.93	59.90	49.83	3.5

	P-values	FDR	t0	CFD	CRD	MYC	NFB	NFB+MYC	LDA score
Sideroxydans	0.00429	0.01557	3051.34	100.00	135.68	213.15	330.85	216.67	3.2
Simplicispira	0.03159	0.06918	9.76	100.00	266.26	88.94	178.07	101.12	2.64
Skermanella	0.01553	0.03951	103.66	100.00	103.95	117.56	139.40	164.68	4.21
Solibacillus	0.00542	0.01887	184.49	100.00	123.61	106.00	88.34	147.43	2.16
Sorangium	0.00668	0.02164	140.08	100.00	150.55	94.70	116.27	87.19	2.86
Sphingoaurantiacus	0.01111	0.03082	76.89	100.00	94.62	104.02	100.28	136.32	2.87
Sphingobium	0.00000	0.00031	4.17	100.00	68.65	78.55	53.59	61.22	4.46
Sphingopyxis	0.00006	0.00145	25.81	100.00	119.16	73.17	72.49	54.77	3.46
Spirochaeta	0.00119	0.00674	960.78	100.00	80.90	162.35	115.96	110.19	2.91
Sporichthya	0.03114	0.06862	38.33	100.00	94.96	89.07	82.17	92.00	2.34
Sporosarcina	0.01384	0.03647	162.45	100.00	104.71	115.40	110.37	147.62	2.88
Stenotrophomonas	0.00264	0.01135	88.90	100.00	52.89	27.38	33.96	9.08	2.62
Streptomyces	0.00017	0.00217	66.89	100.00	93.75	75.24	82.22	85.77	4.02
Symbiobacterium	0.02408	0.05658	224.38	100.00	115.78	113.34	107.82	151.93	2.68
Synechococcus IR11	0.00001	0.00059	3.69	100.00	341.99	190.17	329.76	389.19	3.37
Syntrophobacter	0.00815	0.02477	305.41	100.00	75.81	88.20	91.25	113.32	2.77
Tahibacter	0.00395	0.01455	25.53	100.00	95.15	56.27	110.06	75.57	2.91
Tepidisphaera	0.01871	0.04559	52.98	100.00	90.57	105.84	92.08	78.76	3.09
Terribacillus	0.00075	0.00474	178.75	100.00	143.36	130.01	130.46	310.67	2.81
Terrimicrobium	0.00078	0.00482	31.58	100.00	103.48	84.10	95.57	83.94	2.65
Terrimonas	0.00064	0.00437	52.79	100.00	109.92	95.99	100.83	84.87	3.71
Tetradesmus obliquus	0.00069	0.00453	19.78	100.00	74.56	117.06	103.05	101.42	2.27
Thermoactinomyces	0.02854	0.06422	291.52	100.00	156.12	141.98	111.22	129.71	2.68
Thermoflavimicrobium	0.02207	0.05278	252.59	100.00	161.03	142.69	128.76	200.32	2.62
Thermopolyspora	0.04261	0.08539	325.22	100.00	167.33	160.09	199.93	195.96	2.04
Truepera	0.00069	0.00453	20.46	100.00	119.24	118.48	75.29	127.37	3.26
Tumebacillus	0.04108	0.08329	249.07	100.00	145.95	121.42	127.70	167.07	3.67
Vampirovibrio	0.00016	0.00217	4.47	100.00	90.75	81.91	90.58	75.10	2.36
Variovorax	0.00629	0.02092	50.87	100.00	85.98	75.91	74.01	73.38	3.52
Verrucomicrobia bacterium (uncultured)	0.03621	0.07701	38.18	100.00	81.07	86.26	73.81	61.58	2.44
Verrucomicrobiales bacterium (uncultured)	0.00262	0.01135	20.29	100.00	75.16	83.14	91.51	65.02	2.35
Vischeria sp CAUP Q 202	0.00036	0.00315	31.09	100.00	106.89	121.84	62.84	179.28	2.45
Waddlia	0.03765	0.07912	110.38	100.00	85.97	74.47	61.71	63.55	2.48
Yonghaparkia	0.00478	0.01689	17.11	100.00	86.97	108.02	76.62	65.89	3.33

Table S7. Signature associated to each treatment. Data grouped by treatment. Percentual standardization of values vs. CRD. Negative differences greater than 10% are highlighted in red, while positive differences greater than 10% are highlighted in green.

	P-values	FDR	t0	CFD	CRD	MYC	NFB	NFB+MYC	LDA score
Acidibacter	0.00135	0.00721	53.01	83.65	100.00	80.93	98.86	85.86	3.74
Acidimicrobiia bacterium (uncultured)	0.00034	0.00310	180.30	97.05	100.00	89.93	94.83	96.95	4.02
Acidobacteriales bacterium (uncultured)	0.00814	0.02477	132.89	93.41	100.00	103.00	112.99	86.79	4.17
Acidothermus	0.00004	0.00110	827.36	105.09	100.00	70.25	98.68	107.10	2.83
Acidovorax	0.00017	0.00217	17.55	92.25	100.00	107.15	85.53	50.97	3.18
Actinocorallia	0.00037	0.00321	129.88	68.98	100.00	76.61	97.02	46.34	2.99
Actinomadura	0.04147	0.08374	139.62	99.24	100.00	100.77	109.12	109.81	2.62
Adhaeribacter	0.02146	0.05156	93.24	106.88	100.00	128.84	119.59	146.31	3.62
Aeromicrobium	0.00062	0.00437	42.94	114.80	100.00	98.94	84.46	87.05	4.42
Allorhizobium, Neorhizobium, Pararhizobium, Rhizobium	0.00061	0.00437	49.03	103.82	100.00	98.39	102.12	106.50	4.34
Alsobacter	0.00583	0.01964	80.85	88.98	100.00	121.11	113.99	136.24	2.86
Altererythrobacter	0.00026	0.00280	36.73	80.83	100.00	69.79	72.97	70.21	3.51
Amaricoccus	0.00411	0.01502	83.21	97.59	100.00	106.91	117.88	154.79	3.27
Aminobacter	0.00000	0.00009	46.62	90.07	100.00	248.10	113.61	218.34	3.27
Ammoniphilus	0.00565	0.01921	106.11	80.66	100.00	75.61	76.21	110.34	2.77
Amycolatopsis	0.00240	0.01076	19.68	283.38	100.00	137.91	134.99	161.86	3.37
Anaerobacterium	0.01125	0.03106	310.52	86.87	100.00	106.39	89.03	111.64	2.71
Anaerolinea	0.00972	0.02788	508.51	120.74	100.00	134.16	139.09	214.12	3.76
Anaeromyxobacter	0.00150	0.00749	181.19	98.08	100.00	103.29	110.69	125.05	3.54
Anaeromyxobacter dehalogenans	0.01014	0.02859	47.19	55.69	100.00	114.74	76.24	118.52	1.93
Aquicella	0.00069	0.00453	29.19	116.82	100.00	87.12	94.59	65.06	2.59
Aquisphaera	0.00816	0.02477	201.86	98.00	100.00	105.69	103.41	104.65	2.73
Arenimonas	0.00036	0.00315	37.49	106.11	100.00	88.75	68.62	89.01	3.4
Aridibacter	0.01146	0.03146	72.33	115.69	100.00	103.47	114.26	118.92	3.1
Armatimonas	0.00325	0.01266	14.07	89.98	100.00	113.23	86.29	97.03	1.88
Arthrobacter	0.00246	0.01081	101.09	119.57	100.00	133.18	115.26	163.74	5.2
Asticcacaulis	0.00906	0.02657	14.25	41.75	100.00	46.83	40.32	30.43	3.01
Aurantisolimonas	0.01847	0.04523	73.04	105.35	100.00	110.21	111.75	126.08	2.74
Azohydromonas	0.00158	0.00781	42.57	101.90	100.00	97.48	92.51	108.00	3.29
Azospira	0.00652	0.02145	1.24	58.73	100.00	73.18	68.42	31.24	2.58
Bacillus	0.03987	0.08147	92.18	97.79	100.00	90.31	83.60	133.05	4.92
Bauldia	0.01432	0.03736	41.62	61.65	100.00	57.67	72.07	52.42	3.16
Bdellovibrio	0.00114	0.00647	39.72	87.21	100.00	88.48	89.70	104.58	2.92
Blastocatella	0.00211	0.00978	74.17	112.70	100.00	115.67	117.10	105.68	3.35
Bosea	0.00039	0.00329	45.54	94.31	100.00	102.15	95.36	118.28	3.54

	P-values	FDR	t0	CFD	CRD	MYC	NFB	NFB+MYC	LDA score
Bradyrhizobium	0.00270	0.01138	63.22	89.66	100.00	90.10	102.98	88.75	3.84
Brevifollis	0.00671	0.02164	237.53	73.70	100.00	67.65	45.31	108.47	2.25
Burkholderia Caballeronia Paraburkholderia	0.02324	0.05508	137.96	86.38	100.00	62.88	60.42	90.02	3.34
Burkholderiales bacterium Beta 02	0.00021	0.00248	26.32	144.74	100.00	83.37	79.18	79.56	2.23
Caenimonas	0.00017	0.00217	58.06	108.67	100.00	105.99	103.24	125.79	4.01
Caldicoprobacter	0.04294	0.08572	177.55	95.66	100.00	102.04	86.12	126.62	2.68
Candidatus Accumulibacter	0.01659	0.04139	331.73	97.30	100.00	128.36	130.56	129.63	2.33
Candidatus Chloroploca	0.01106	0.03082	62.47	115.41	100.00	134.28	137.23	147.73	2.77
Candidatus Entotheonella	0.02561	0.05934	70.79	117.15	100.00	91.27	125.40	103.11	2.72
Candidatus Saccharibacteria bacterium (uncultured)	0.04738	0.09386	76.63	167.09	100.00	132.49	105.83	147.79	3.23
Candidatus Udaeobacter	0.04927	0.09649	105.90	96.39	100.00	85.11	97.71	62.78	3.92
Chelativorans	0.00000	0.00000	61.60	85.02	100.00	2135.41	116.96	2182.48	3.35
Chlamydomonas reinhardtii	0.00279	0.01149	13.65	81.76	100.00	102.72	56.32	163.89	2.13
Chloroflexi bacterium (uncultured)	0.03765	0.07912	126.96	100.71	100.00	102.78	103.00	113.37	4.07
Chloronema	0.00002	0.00075	22.03	61.54	100.00	154.82	128.89	117.14	3.01
Chthonobacter	0.00855	0.02567	25.05	80.70	100.00	87.96	76.26	83.12	2.47
Chthonomonas	0.00206	0.00964	75.66	90.55	100.00	98.30	102.13	112.54	2.87
Clostridium sensu stricto 1	0.03619	0.07701	232.14	121.12	100.00	115.57	120.04	147.17	3.17
Clostridium sensu stricto 8	0.02914	0.06518	172.52	97.43	100.00	100.93	103.21	132.04	2.86
Cnuella	0.00138	0.00731	36.46	99.04	100.00	104.15	87.03	119.81	2.55
Cohnella	0.00555	0.01919	155.11	81.94	100.00	97.41	102.65	126.98	3.46
Comamonas	0.00277	0.01149	35.93	110.96	100.00	96.42	93.38	96.39	2.91
Conexibacteraceae bacterium (uncultured)	0.00101	0.00613	198.29	113.50	100.00	108.43	122.40	80.80	2.42
Croceicoccus	0.00671	0.02164	16.47	69.97	100.00	82.93	76.11	80.95	2.45
Cupriavidus	0.00783	0.02447	31.84	60.53	100.00	63.81	67.01	96.89	3.35
Dactylosporangium	0.03391	0.07334	164.60	100.70	100.00	89.90	108.95	137.17	2.61
Deinococcus	0.00064	0.00437	0.32	160.66	100.00	50.36	26.44	55.19	3.25
delta proteobacterium WX81	0.01172	0.03202	207.19	119.08	100.00	115.85	114.71	99.57	2.25
Deltaproteobacteria bacterium GWC2 55 46	0.00127	0.00702	471.04	128.08	100.00	122.19	139.49	214.91	2.47
Desmochloris halophila	0.00010	0.00191	4.31	146.04	100.00	100.23	98.14	110.20	3.21
Desulfosporosinus	0.02631	0.06042	242.29	79.01	100.00	88.73	96.10	131.44	2.32
Desulfuromonadaceae bacterium (uncultured)	0.04409	0.08767	227.80	101.45	100.00	96.08	93.25	97.34	2.09
Desulfuromonadales bacterium (uncultured)	0.02821	0.06393	152.80	103.90	100.00	106.93	132.45	125.34	3.07
Devosia	0.00000	0.00031	36.19	84.19	100.00	71.03	73.63	65.65	4.21
Dokdonella	0.00012	0.00194	0.00	37.97	100.00	43.94	79.16	35.19	2.53

	P-values	FDR	t0	CFD	CRD	MYC	NFB	NFB+MYC	LDA score
Dongia	0.03324	0.07219	125.80	92.78	100.00	86.31	96.63	83.80	3.54
Dyadobacter	0.00071	0.00458	41.72	161.46	100.00	187.58	74.42	78.12	3.71
Edaphochlorella mirabilis	0.03746	0.07912	14.03	84.35	100.00	44.79	41.91	43.65	2.71
Ellin517	0.00016	0.00217	40.34	107.93	100.00	87.63	86.63	71.93	3.76
Ellin6055	0.01512	0.03866	66.97	114.88	100.00	101.96	98.99	105.23	3.91
Ellin6067	0.01279	0.03459	50.22	96.23	100.00	90.27	83.51	80.56	3.69
Ensifer	0.00103	0.00613	56.68	100.93	100.00	109.29	100.35	101.28	3.7
Erythrobacter	0.00013	0.00206	2.41	90.31	100.00	157.34	70.45	92.08	2.86
Ettlia pseudoalveolaris	0.00226	0.01027	19.64	132.63	100.00	127.83	102.68	422.12	3.06
Euzebya	0.04205	0.08460	52.77	107.77	100.00	74.13	82.78	79.07	1.83
Ferrovibrio	0.00285	0.01164	33.16	104.62	100.00	84.50	106.35	87.54	2.4
Fictibacillus	0.00028	0.00290	150.44	70.62	100.00	72.20	79.08	98.06	3.69
Fimbriiglobus	0.01002	0.02841	68.86	96.38	100.00	101.68	105.73	88.15	3.7
Flavihumibacter	0.01403	0.03676	52.62	98.05	100.00	117.35	120.10	129.34	2.32
Flavisolibacter	0.00964	0.02780	68.22	103.10	100.00	106.37	102.29	106.17	3.68
Flavitalea	0.00005	0.00128	41.37	89.29	100.00	100.93	97.59	94.72	3.23
Flavobacterium	0.00143	0.00737	32.17	112.19	100.00	79.11	47.80	73.32	3.27
Gaiella	0.00123	0.00684	162.70	97.59	100.00	96.70	97.48	102.17	4.85
Gemmatimonas	0.00022	0.00252	56.09	101.48	100.00	106.29	101.89	107.85	3.82
Geoalkalibacter	0.00024	0.00268	983.00	111.45	100.00	181.90	195.63	221.99	2.98
Green Bay ferromanganous micronodule bacterium MND4 (uncultured)	0.01637	0.04105	186.94	89.33	100.00	84.69	119.05	77.34	2.05
Halobacillus	0.00341	0.01302	3.07	230.06	100.00	344.11	429.58	50.03	3.22
Herbinix	0.00653	0.02145	176.57	92.26	100.00	87.19	103.27	130.25	2.78
Herpetosiphon	0.01339	0.03583	62.70	115.20	100.00	109.37	92.36	100.64	3.72
Hirschia	0.00047	0.00361	13.30	44.02	100.00	49.72	58.50	45.73	3.56
Hungateiclostridium	0.01365	0.03634	182.20	84.45	100.00	104.52	101.47	183.35	1.9
Hyaloperonospora arabidopsidis	0.00042	0.00339	15.29	125.89	100.00	93.53	97.66	81.29	2.24
Hymenobacter	0.01716	0.04253	92.04	73.98	100.00	159.72	115.60	177.64	2.14
Iamia	0.02128	0.05136	106.51	107.51	100.00	87.53	89.98	80.05	3.49
Ideonella	0.00064	0.00437	13.81	57.55	100.00	66.66	62.39	50.17	2.96
Inquilinus	0.00465	0.01676	25.77	46.64	100.00	50.65	44.60	71.60	2.56
Knoellia	0.02859	0.06422	127.38	109.65	100.00	105.65	109.23	124.89	4.05
Kribbella	0.00474	0.01686	130.76	101.65	100.00	95.69	96.13	94.59	2.88
Lacibacter	0.00473	0.01686	53.78	134.35	100.00	108.04	84.76	95.20	3.24
Lautropia	0.01305	0.03508	58.90	112.59	100.00	107.92	126.92	131.93	2.46
Lechevalieria	0.00109	0.00633	50.17	126.59	100.00	76.63	87.11	76.59	3.72
Legionella	0.00111	0.00643	34.65	80.74	100.00	83.86	83.68	80.65	2.95
Leptolyngbya EcFYyyy 00	0.00001	0.00055	2.52	60.37	100.00	192.12	211.85	162.57	4.5

	P-values	FDR	t0	CFD	CRD	MYC	NFB	NFB+MYC	LDA score
Litorilinea	0.04870	0.09609	81.94	106.59	100.00	107.96	110.15	121.48	3.35
Longimicrobium	0.00379	0.01424	25.35	69.02	100.00	127.61	98.81	100.10	2.9
Luedemannella	0.00064	0.00437	202.97	90.57	100.00	92.02	96.64	120.42	3.41
Luteimonas	0.00011	0.00194	32.22	107.77	100.00	55.44	63.07	47.44	2.76
Luteolibacter	0.00033	0.00310	26.62	104.66	100.00	75.80	78.45	54.46	4.03
Lysobacter	0.01745	0.04292	75.21	112.72	100.00	101.27	90.31	102.32	3.81
Marmoricola	0.00385	0.01431	127.88	99.73	100.00	94.80	93.25	104.07	3.98
Massilia	0.03121	0.06862	97.10	100.24	100.00	110.31	97.92	138.60	4
Meiothermus	0.00309	0.01226	0.86	181.35	100.00	94.28	222.60	102.62	2.5
Mesorhizobium	0.00003	0.00095	35.57	72.36	100.00	75.67	83.57	78.70	4.13
Methylobacillus	0.00008	0.00168	3.96	250.98	100.00	150.67	60.46	129.58	3.18
Methyloceanibacter	0.01474	0.03805	148.47	93.08	100.00	105.83	100.15	73.83	2.19
Methylotenera	0.00002	0.00075	23.77	202.42	100.00	125.85	88.91	211.44	3.57
Microbacterium	0.00265	0.01135	53.07	145.17	100.00	94.46	69.87	87.85	3.78
Micropepsis	0.00018	0.00217	0.47	37.32	100.00	37.80	58.66	28.48	2.84
Microvirga	0.00245	0.01081	75.57	92.13	100.00	105.42	106.78	113.02	4.43
Mitsuaria	0.00006	0.00145	2.54	227.07	100.00	280.60	133.84	69.67	3.87
Mycobacterium	0.00128	0.00702	108.97	84.65	100.00	84.02	89.67	86.06	3.65
Nakamurella	0.03916	0.08131	112.07	100.97	100.00	116.08	122.41	150.54	2.49
Nannocystis	0.00698	0.02208	48.37	80.54	100.00	89.59	116.23	78.22	2.46
Niastella	0.00012	0.00194	45.33	100.81	100.00	95.30	105.48	75.58	3.17
Nitratireductor	0.00829	0.02501	63.80	114.41	100.00	133.22	114.53	115.00	2.35
Nitrosomonas	0.00011	0.00194	24.04	100.28	100.00	126.15	135.19	124.14	2.82
Nitrospira	0.02496	0.05835	84.10	92.22	100.00	108.03	111.35	93.84	4.01
Nodosilinea PCC 7104	0.00001	0.00040	1.71	70.67	100.00	89.85	82.85	177.11	3.82
Nordella	0.01623	0.04090	76.60	96.26	100.00	93.59	103.95	80.68	3.65
Noviherbaspirillum	0.00312	0.01227	72.31	109.34	100.00	120.72	105.01	131.98	3.9
Ohtaekwangia	0.00348	0.01319	43.75	91.17	100.00	81.09	75.03	77.65	3.62
Oscillatoria SAG 1459 8	0.00029	0.00290	3.14	76.06	100.00	125.46	153.79	141.79	2.55
Oscillochloris	0.00218	0.01001	28.42	108.46	100.00	90.02	80.46	93.39	2.27
Paenarthrobacter	0.03870	0.08099	109.44	189.92	100.00	146.91	117.25	165.80	3.51
Paenibacillus	0.03486	0.07476	112.50	92.92	100.00	92.65	95.64	127.53	4.14
Paenisporosarcina	0.02545	0.05922	134.37	88.82	100.00	87.21	89.44	115.87	3.29
Panacagrimonas	0.00140	0.00732	27.37	124.35	100.00	134.68	89.24	83.93	2.69
Parasegetibacter	0.00001	0.00061	51.58	103.28	100.00	126.21	98.82	148.10	3.22
Paucibacter	0.00386	0.01431	47.16	117.79	100.00	104.86	106.83	113.53	2.62
Pedococcus Phycicoccus	0.00988	0.02817	155.05	129.34	100.00	101.96	111.64	110.68	2.76
Pelomonas	0.00078	0.00482	22.15	95.98	100.00	75.78	64.17	86.19	2.63
Pelosinus	0.00331	0.01284	211.07	93.42	100.00	87.23	96.81	111.81	2.84

	P-values	FDR	t0	CFD	CRD	MYC	NFB	NFB+MYC	LDA score
Peredibacter	0.00887	0.02630	60.62	111.43	100.00	107.15	104.88	107.37	2.9
Permianibacter	0.04910	0.09649	42.11	139.55	100.00	108.10	42.91	84.87	2.12
Pirellula	0.00943	0.02735	77.57	104.52	100.00	94.30	94.83	72.69	4.33
Piscinibacter	0.00304	0.01214	48.54	127.99	100.00	108.91	102.46	129.51	2.6
Planctomicrobium	0.00693	0.02206	54.41	122.84	100.00	90.92	84.48	63.52	2.74
Planctomycetales bacterium (uncultured)	0.00004	0.00110	50.72	83.67	100.00	85.29	93.00	59.69	3.01
Planctomycete (uncultured)	0.00014	0.00210	53.75	107.22	100.00	98.87	96.82	87.33	4.19
Planctomycete WY108	0.00177	0.00835	38.82	141.84	100.00	139.50	121.91	124.12	2.06
Planctopirus	0.00001	0.00061	19.14	71.17	100.00	56.14	80.63	58.72	3.34
Planoglabratella opercularis	0.00046	0.00360	24.34	109.85	100.00	129.74	151.57	136.66	2.44
Polyangiaceae bacterium (uncultured)	0.01496	0.03843	22.55	92.43	100.00	82.53	74.55	48.66	2.05
Polyangium brachysporum group	0.00007	0.00158	18.48	93.17	100.00	67.71	72.58	64.55	3.44
Pontibacter	0.02821	0.06393	71.52	117.49	100.00	127.82	120.07	145.43	3.53
prokaryote (uncultured)	0.00048	0.00361	233.67	97.66	100.00	108.26	115.05	120.22	3.31
Promicromonospora	0.00609	0.02040	108.79	76.51	100.00	53.88	52.82	60.01	3.1
Prosthecomicrobium	0.00149	0.00749	44.32	127.88	100.00	93.01	101.90	121.35	2.37
proteobacterium (uncultured)	0.03905	0.08131	172.50	104.51	100.00	97.99	105.55	94.68	3.38
Pseudarthrobacter	0.00689	0.02206	96.50	135.69	100.00	149.45	116.49	169.62	3.22
Pseudoduganella	0.00000	0.00031	15.93	123.09	100.00	154.72	83.25	53.66	3.42
Pseudoflavitalea	0.00752	0.02367	45.41	54.15	100.00	63.89	80.07	73.03	2.97
Pseudomuriella schumacherensis	0.00002	0.00072	10.46	86.57	100.00	116.79	148.00	139.05	3.36
Pseudorhodoplanes	0.00026	0.00280	58.23	78.01	100.00	84.07	101.20	71.96	2.96
Pseudoxanthomonas	0.00026	0.00280	29.97	96.34	100.00	77.23	61.17	63.15	3.76
Qipengyuania	0.00794	0.02467	53.98	128.80	100.00	119.15	97.79	112.69	3.51
Ramlibacter	0.00015	0.00213	50.45	104.44	100.00	106.38	97.17	108.04	3.9
Reyranella	0.00034	0.00310	56.03	80.80	100.00	84.99	87.17	68.99	3.5
Rhizobacter	0.00006	0.00145	51.47	101.23	100.00	95.14	89.99	104.60	3.82
Rhizorhapis	0.03420	0.07365	18.95	98.86	100.00	89.02	57.96	38.85	2.53
Rhodanobacter	0.00032	0.00310	0.52	32.13	100.00	30.10	61.96	39.62	3.34
Rhodobacter	0.00561	0.01921	44.13	93.61	100.00	117.09	118.71	134.89	3.19
Rhodococcus	0.00295	0.01185	175.09	118.49	100.00	74.63	91.84	104.71	2.86
Rhodocytophaga	0.00141	0.00732	54.29	106.73	100.00	122.89	105.77	128.23	3.15
Rhodopirellula	0.00173	0.00822	52.93	101.13	100.00	94.45	96.01	90.58	3.4
Rhodopseudomonas	0.00010	0.00190	6.83	52.42	100.00	51.39	84.26	66.59	2.73
Roseomonas	0.00277	0.01149	65.76	98.63	100.00	112.94	111.96	127.73	2.97
Rubellimicrobium	0.00012	0.00196	61.92	116.47	100.00	131.65	124.49	152.66	3.87
Rubrobacterales bacterium (uncultured)	0.00040	0.00329	169.16	99.90	100.00	87.09	95.43	87.14	4.26
Rubrobacteria bacterium (uncultured)	0.00927	0.02704	145.33	94.55	100.00	92.38	95.01	94.98	3.69
Ruminiclostridium	0.00056	0.00413	238.15	84.68	100.00	111.05	114.51	145.27	3.09

	P-values	FDR	t0	CFD	CRD	MYC	NFB	NFB+MYC	LDA score
Salinispora	0.00566	0.01921	180.74	88.17	100.00	96.76	130.32	96.32	2.46
Schlesneria	0.00287	0.01166	3.00	78.16	100.00	69.65	112.37	43.85	2.27
Shinella	0.00000	0.00027	14.07	97.94	100.00	75.35	58.67	48.81	3.5
Sideroxydans	0.00429	0.01557	2248.91	73.70	100.00	157.10	243.85	159.69	3.2
Simplicispira	0.03159	0.06918	3.67	37.56	100.00	33.40	66.88	37.98	2.64
Skermanella	0.01553	0.03951	99.72	96.20	100.00	113.10	134.10	158.42	4.21
Solibacillus	0.00542	0.01887	149.26	80.90	100.00	85.76	71.47	119.28	2.16
Sorangium	0.00668	0.02164	93.05	66.42	100.00	62.90	77.23	57.91	2.86
Sphingoaurantiacus	0.01111	0.03082	81.26	105.69	100.00	109.93	105.98	144.07	2.87
Sphingobium	0.00000	0.00031	6.08	145.66	100.00	114.42	78.06	89.18	4.46
Sphingopyxis	0.00006	0.00145	21.66	83.92	100.00	61.41	60.83	45.96	3.46
Spirochaeta	0.00119	0.00674	1187.60	123.61	100.00	200.68	143.33	136.21	2.91
Sporichthya	0.03114	0.06862	40.36	105.31	100.00	93.80	86.54	96.88	2.34
Sporosarcina	0.01384	0.03647	155.14	95.50	100.00	110.21	105.40	140.98	2.88
Stenotrophomonas	0.00264	0.01135	168.08	189.07	100.00	51.76	64.22	17.17	2.62
Streptomyces	0.00017	0.00217	71.35	106.67	100.00	80.25	87.70	91.49	4.02
Symbiobacterium	0.02408	0.05658	193.80	86.37	100.00	97.89	93.12	131.23	2.68
Synechococcus IR11	0.00001	0.00059	1.08	29.24	100.00	55.61	96.42	113.80	3.37
Syntrophobacter	0.00815	0.02477	402.88	131.92	100.00	116.35	120.38	149.49	2.77
Tahibacter	0.00395	0.01455	26.83	105.09	100.00	59.13	115.66	79.42	2.91
Tepidisphaera	0.01871	0.04559	58.50	110.41	100.00	116.87	101.67	86.97	3.09
Terribacillus	0.00075	0.00474	124.68	69.75	100.00	90.68	91.00	216.70	2.81
Terrimicrobium	0.00078	0.00482	30.52	96.63	100.00	81.27	92.35	81.11	2.65
Terrimonas	0.00064	0.00437	48.03	90.98	100.00	87.33	91.73	77.21	3.71
Tetradesmus obliquus	0.00069	0.00453	26.53	134.11	100.00	156.99	138.21	136.02	2.27
Thermoactinomyces	0.02854	0.06422	186.72	64.05	100.00	90.94	71.24	83.08	2.68
Thermoflavimicrobium	0.02207	0.05278	156.86	62.10	100.00	88.61	79.97	124.40	2.62
Thermopolyspora	0.04261	0.08539	194.36	59.76	100.00	95.67	119.48	117.11	2.04
Truepera	0.00069	0.00453	17.16	83.86	100.00	99.36	63.14	106.81	3.26
Tumebacillus	0.04108	0.08329	170.66	68.52	100.00	83.19	87.50	114.47	3.67
Vampirovibrio	0.00016	0.00217	4.93	110.19	100.00	90.26	99.81	82.75	2.36
Variovorax	0.00629	0.02092	59.16	116.30	100.00	88.29	86.08	85.34	3.52
Verrucomicrobia bacterium (uncultured)	0.03621	0.07701	47.10	123.35	100.00	106.41	91.04	75.95	2.44
Verrucomicrobiales bacterium (uncultured)	0.00262	0.01135	26.99	133.05	100.00	110.62	121.75	86.51	2.35
Vischeria sp CAUP Q 202	0.00036	0.00315	29.08	93.56	100.00	113.99	58.79	167.73	2.45
Waddlia	0.03765	0.07912	128.39	116.32	100.00	86.62	71.78	73.92	2.48
Yonghaparkia	0.00478	0.01689	19.67	114.98	100.00	124.20	88.09	75.76	3.33
